# The impact of the digital economy on food system resilience: Evidence from China

**DOI:** 10.1371/journal.pone.0311689

**Published:** 2024-10-24

**Authors:** Zheng Zhu, Zhen Wang, Song Yu, Zhaomei Tang, Bin Liu

**Affiliations:** 1 Jiangxi Daily, Nanchang, Jiangxi, China; 2 School of Economics & Management, Jiangxi Agricultural University, Nanchang, Jiangxi, China; 3 School of Economics, Sichuan Agricultural University, Chengdu, Sichuan, China; 4 YuZhang Normal University, Nanchang, Jiangxi, China; Gebze Teknik Universitesi, TÜRKIYE

## Abstract

Despite the widespread influence of the burgeoning digital economy on agricultural productivity in recent years, China’s food system confronts numerous challenges. Notably, research exploring the digital economy’s impact on food system resilience remains scarce, and the pivotal role of industrial agglomeration in this context remains unclear. Therefore, this article is based on the panel data of 30 provinces in China from 2011 to 2021, this paper empirically examines the direct effect, the mechanism of action, and the spatial spillover effect of the resilience of a digital economy-enabled food system using a double fixed-effects model, mediated-effects model, and spatial econometric model. The results show the following: (1) The resilience of China’s urban food system shows obvious spatial differences, but the overall trend in improvement requires attention. (2) The development of a digital economy has a facilitating effect on the level of resilience of the food system, and industrial agglomeration induces an intermediary effect. (3) The digital economy has a significant positive spatial spillover effect on the resilience of the food system; i.e., the digital economy can improve the resilience of the food system in the region and the neighboring regions. Accordingly, policy recommendations have been put forward to improve infrastructure construction and promote the development of digital villages; strengthen the construction of industrial agglomerations and promote the enhancement of the quality and efficiency of industries; and promote the development of regional linkages and build a solid foundation for food security.

## 1. Introduction

Food security is “the most important thing in the country”, and its major strategic role has a bearing on economic development and social stability; it also serves as an important foundation for national security. Since 2015, China has achieved sustained growth in total grain output, reaching a new level of 650 million tons. By 2023, it was reached over 690 million tons, despite possessing less than 10% of the world’s arable land to feed nearly 20% of the world’s population. This is a remarkable feat and provides a powerful answer to the question of “who will feed China” [1]. Ensuring China’s food security remains an urgent priority despite continuous improvements in the country’s comprehensive food production efficiency. This is due to multiple constraints, such as domestic shortages of arable land, water, and other resources, and international geopolitical conflicts and a global economic slowdown [2]. The “2023 State of Food Security and Nutrition in the World” report by the United Nations highlights the need to enhance resilience to tackle food crises. Similarly, “Central Document No. 1 in 2023” emphasizes the importance of strengthening agriculture as a means of building a resilient industrial sector and a strong agricultural nation. Promoting the construction of a resilient food system is a path toward resisting and quickly recovering from risky shocks, shifting to a new growth path, achieving adaptive food development, and guaranteeing a balance between food production and supply while eliminating the root causes of food insecurity [3]. However, the food industry is often associated with low-quality and inefficient production processes, making it challenging to enhance system resilience solely through its own efforts. In recent years, the digital economy has gradually penetrated all areas of the national economy and social development through cloud computing, big data, artificial intelligence, and other core high and new technologies. Its deep integration with agriculture provides new opportunities and challenges for enhancing the resilience of the food system. Empowering the food system with the digital economy will become an important direction for the future development of agriculture [4]. Furthermore, expediting the growth of the digital economy can strengthen the resilience of the food system. It is important to note that industrial agglomeration plays a crucial role in this process [5]. By leveraging the economic scale’s externality, the specialization of the division of labor, and knowledge spillover, industrial agglomeration can enhance the efficiency of food production, improve the industry’s competitiveness and risk management capabilities, and ultimately bolster the resilience of food systems [6]. Therefore, conducting an in-depth investigation into the impact and internal mechanisms of the digital economy on the resilience of the food system is of great theoretical and practical significance in guaranteeing national food security and comprehensively promoting rural revitalization.

There are three main types of literature related to the research theme of this paper. The first category is the construction of a food system resilience indicator system. Early scholars believe that food system resilience is mainly the resistance ability after coping with unexpected shocks (such as natural disasters, financial, and political crises) [7]. Some scholars have also proposed that food system resilience indicates the risk adaptive capacity that can systematically anticipate and withstand pressure [8]. Hao et al, on the basis of the three dimensions of resistance, adaptive capacity, and transformative capacity, constructed an index of China’s food system resilience [9]. Other scholars have also studied food production resilience [10], cropland system protection resilience [11], and food security resilience [12], providing important references for this paper. The second category focuses on the study of the digital economy and its impact on food system resilience. Some scholars argue that digital village construction [9], digital financial inclusion [13], and digital finance [14] are key factors in enhancing the resilience of the food system. Others believe that the digital economy is the core element in empowering the high-quality development of the food industry [15], improving the resilience of the food supply chain [16], and increasing food security capacity [17]. This suggests that it could be a significant opportunity for fostering the growth of the digital economy and, in turn, strengthening the resilience of the food system. The third category studies the role of industrial agglomeration paths. One view is that the digital economy can mobilize resources, break through spatial and temporal limitations, and promote the optimization and integration of the food industry chain, thereby weakening the role of industrial agglomeration [18]. Another view is that the food industry has a higher degree of external risk and relies more on the knowledge spillover effect of the digital economy and the function of the rapid exchange of information to improve risk management and decision-making efficiency. The digital economy exacerbates the “siphon effect,” where a city’s competitive advantages, including digital technologies and information flow, draw resources like businesses, technologies, and knowledge from surrounding areas, resulting in their concentration within the city and leading to the formation of a network system to jointly protect against risks. To improve risk management and decision-making efficiency, operators in the food industry, along with technology and knowledge experts, will be gathered in a specific region to form a network system to jointly resist risks. This promotes industrial agglomeration in the digital economy [19].

The literature review shows that previous studies are a valuable reference for this paper. However, there are still some shortcomings that need to be addressed: Notably, while China has placed significant emphasis on the development of the digital economy and achieved notable successes, the supporting and enabling role of the digital economy in promoting high-quality industrial development has become increasingly evident. However, research examining the resilience of the digital economy in relation to the food system remains scarce. Additionally, there is a paucity of studies that have delved into the intrinsic linkages among the three domains, specifically including the dimension of industrial agglomeration, thereby neglecting to comprehensively explore their interconnectedness. Therefore, this paper presents a theoretical analytical framework for exploring the resilience of the current food system in the context of the digital economy, industrial agglomeration, and spatial and temporal characteristics. The framework examines the impact effects, internal mechanisms, and spatial spillover effects of the food system’s resilience. By analyzing these elements and proposing countermeasures, this paper aims to provide a scientific basis for ensuring China’s food security.

## 2. Theoretical analysis and research hypothesis

### 2.1. Direct effects of the digital economy on food system resilience

The theory of network economics posits that the data network is a productive tool and that market behavior, information acquisition, decision making, and trading are all closely linked to the data network. This is particularly true in the digital economy era, where the trend toward networked food production and operational activities is increasingly apparent. The integration of substitution effects enhances the resilience of the food system in the digital economy. This is achieved through networking, high-permeability effects, and sustainable practices, as well as information synergy, innovation, and creation effects. (1) It is important to consider the integration of substitution effects in this context. Urbanization has led to a loss of agricultural labor, resulting in the abandonment of arable land and a double predicament comprising a “shortage of resources” and “idle resources”. The digital economy, with data as the core factor of production, differs from the traditional labor force and resource factors. By replacing inefficient production factors, it increases factor inputs and promotes the effective use of resources. Additionally, the deep penetration and integration of the digital economy and the food industry can effectively empower the digital transformation and upgrading of the food industry. This improves the value of food industry factors and enhances the resistance of the food system [20]. (2) Synergy of information: The digital economy is characterized by networking through the Internet to realize the wide dissemination of information and data mining. It enables the effective management of “data islands” and overcomes data barriers, eliminating information asymmetry and externality problems. This allows food production and operation to utilize their own resources, achieving cross-regional and even cross-industry synergistic configurations and thus promoting decentralization in food production, circulation, sales, and services. This comprehensive extension of the food industry’s chain optimizes the allocation of resources and enhances the adaptability of the food system [21]. (3) Innovation and creation effect: The development of the digital economy has opened a new industrial revolution round, reinventing not only the old organizational structure but also giving rise to new business models. This has promoted the transformation of the employment structure, improved the ecology of the employment market, and created a large number of new jobs for the food system, resulting in a significant creation effect [22]. The digital economy will also create diverse consumer demands and encourage constant reform and innovation in production and operations to adapt to market changes. This will inject new momentum for the transformation of the food system [23]. Therefore, we formulate the following hypothesis:

H1: Digital economic development can empower the enhancement of food system resilience.

### 2.2. Transmission mechanisms of the digital economy to enhance food system resilience

The theory of industrial agglomeration suggests that sharing resources, such as labor, capital, and information, can reduce production costs and promote the formation of industrial agglomerations [24]. The digital economy facilitates universal resource sharing through modern information networks, which contributes to the development of industrial agglomerations. First of all, China’s grain industry agglomeration is still facing problems such as insufficient technological innovation ability, weak market competitiveness, small-scale agglomeration, etc. In the context of the digital economy, high permeability facilitates the efficient flow and precise configuration of resource elements. This enables the effective convergence of upstream and downstream industrial chains, as well as production, supply, and marketing, leading to efficient operations. It also helps enhance the stability and competitiveness of the industrial supply chain, attracting more investment and production operators to enter the field. This provides a key driving force for the agglomeration of the grain industry [19]. Secondly, the digital transformation of infrastructure and the innovation and creation effects of digital technology can broaden the financing, information, and technology acquisition channels of producers and operators. This effectively bridges the industrial development bias caused by differences in resource endowment, environmental regulations, and the level of informatization among regions, thus promoting the development of industrial agglomeration [25].

However, theories such as Marshall-Arrow-Romer (MAR) externality, Porter externality, and Jacob externality highlight the positive impact of industrial agglomeration on economic resilience. MAR externality emphasizes the benefits of industry-specialized agglomeration, while Porter externality and Jacob externality focus on industry-related diversification [26]. This is also applicable to the resilience of the food system. Firstly, industrial agglomeration is led by infrastructure construction, which cannot be separated from facilities such as information systems, logistics and transportation, payment and settlement systems, and a healthy and relaxed development environment [14]. Secondly, industrial agglomeration can promote the construction of an information-sharing platform. This platform can facilitate mutual exchanges and information circulation among production operators in the agglomeration area. For example, it can provide information on market trends and competitor dynamics. This can help production operators better cope with market risks and competitive pressures. Additionally, it can help them adapt to market changes more quickly, thus improving the risk-adaptive capacity of the entire industry. Thirdly, due to the agglomeration economy, operators in the same or similar food production areas become more concentrated, leading to increased competition among producers. This drives continuous self-innovation and the improvement of service quality to enhance competitiveness. Industrial agglomeration provides a specialized external environment for relevant production operators in the region to optimize the allocation of innovation resources; moreover, it stimulates the dynamics of change for the resilience of the food system through the competitive effect [27]. Therefore, the hypothesis proposed is as follows:

H2: Industrial agglomeration contributes to enhancing the resilience of the food system enabled by the digital economy.

### 2.3. Spatial spillover effects of the digital economy on food system resilience

The theory of new economic geography suggests that knowledge and technology spillovers are directional and scope-specific. As a result, economic activities will eventually be evenly distributed across space [28]. Therefore, the impact of the digital economy on the resilience of the food system is not limited to a single city or region but spreads spatially and affects neighboring regions, resulting in a spatial effect [29]. The digital economy relies on the high permeability and network connectivity of digital platforms, driven by information and communication technologies. It reduces information asymmetry, breaks through regional constraints, and boosts the free flow of resource elements between regions. This leads to the Pareto-optimal state of the cross-regional realization of factors such as land, capital, labor, and technology. Simultaneously, the digital economy facilitates the production and operation of online platforms for the food industry, enabling the development of a “dual-track drive” mechanism that links online and offline operations. This reduces the difficulty and cost of accessing information for the main production and operation bodies, enhancing production efficiency and cultivating the resilience of the food system to cope with potential impacts [30]. Moreover, particularly in the field of food production, neighboring regions share similar climate, water, and resource endowments. This results in a stronger role for inter-market and product competition, as well as substitution effects. Technical exchanges and access to information are also more convenient. To achieve a higher competitive position, it is important to actively absorb good practices from neighboring regions and promote synergistic development between different regions. This will improve the quality of food and enhance the resilience of the food system. Therefore, the following hypothesis is formulated:

H3: Digital economy-enabled food system resilience has a positive spatial spillover effect.

## 3. Model construction, variable selection, and data sources

### 3.1. Model construction

#### 3.1.1. Benchmark regression model

In order to verify the impact effect of digital economy-empowering food system resilience, the following benchmark regression model is constructed:

$${\text{FSR}}_{\text{it}}=\alpha_{0}+{\text{cDig}}_{\text{it}}+\beta_{0}\sum {\text{Control}}_{\text{it}}+\mu_{\text{i}}+\pi_{\text{t}}+\varepsilon_{\text{it}}$$ (1)

Eq (1) above shows the relationship between *FSR*_*it*_ and *Dig*_*it*_, representing the resilience of the food system and the digital economy level of province i at time t, respectively. The set of control variables is denoted as ∑*Control*_*it*_, while *α*_0_ is a constant term. The regression coefficients of the corresponding variables are represented by *c* and *β*_0_, respectively. The province and year fixed effects are denoted by *μ*_*i*_ and *π*_*t*_, respectively, and *ε*_*it*_ represents the random error term.

#### 3.1.2. Mediation effects model

To further explore the role mechanism played by industrial agglomeration in the resilience impact of the digital economy-enabled food system, the following mediating effect model is constructed by drawing on the relevant research of Jiang Boat (2022) [31]:

$${\text{Agg}}_{\text{it}}=\alpha_{1}+{\text{aDig}}_{\text{it}}+\beta_{1}\sum {\text{Control}}_{\text{it}}{+\mu }_{\text{i}}{+\pi }_{\text{t}}+\varepsilon_{\text{it}}$$ (2)

$${\text{FSR}}_{\text{it}}=\alpha_{2}+\text{c}\prime {\text{Dig}}_{\text{it}}+{\text{bAgg}}_{\text{it}}+\beta_{2}\sum {\text{Control}}_{\text{it}}{+\mu }_{\text{i}}{+\pi }_{\text{t}}+\varepsilon_{\text{it}}$$ (3)

Eqs (2) and (3) above use the term Agg_it_ to represent the degree of industrial agglomeration in province i at time t. The set of control variables is denoted as ∑*Control*_*it*_. The regression coefficients for the corresponding variables are denoted by a, c’, and b. The remaining equations are consistent with Eq (1).

#### 3.1.3. Spatial measurement models

1. Spatial autocorrelation: Spatial autocorrelation is commonly measured using the global Moran index I. This index ranges from -1 to 1, with values greater than 0 indicating a positive correlation, meaning high—high or low—low neighboring, and values close to 0 indicating no spatial autocorrelation. The formula for Moran index I is as follows:
$$I=\frac{\sum_{i=1}^{n}\sum_{j=1}^{n}W_{ij}\left(x_{i}-\bar{x}\right)\left(x_{j}-\bar{x}\right)}{s^{2}\sum_{i=1}^{n}\sum_{j=1}^{n}W_{ij}}$$ (4)
  In the above Eq (4), $S^{2}=\frac{\sum_{i=1}^{n}{\left(x_{i}-\bar{x}\right)}^{2}}{n}$ is the sample variance, *W*_*ij*_ is the spatial weight matrix, and $\sum_{i=1}^{n}\sum_{j=1}^{n}W_{ij}$ is the sum of all spatial weights; in this paper, we utilize spatial proximity matrix; *x*_*i*_ and *x*_*j*_ denote the values of the indicators of provinces i and j, respectively.
2. Spatial Durbin model: In order to explore the spatial spillover effects of the digital economy and the resilience of the food system, the spatial Durbin model is established as follows:
$${\text{FSR}}_{\text{it}}=\theta_{0}+\lambda W{\text{FSR}}_{\text{it}}+\delta_{1}DIg_{it}+\theta_{1}WDIg_{it}+\delta_{2}\sum {\text{Control}}_{\text{it}}+\theta_{2}W\sum {\text{Control}}_{\text{it}}+\mu_{\text{i}}+\pi_{\text{t}}+\varepsilon_{\text{it}}$$ (5)
  In Eq (5), *λ* is the spatial regression coefficient of the resilience of the food system; *W*(·) is the spatial lag variable; δ and θ are the regression coefficients of each variable; the rest is consistent with Eq (1).

### 3.2. Variable selection

#### 3.2.1. Dependent variable: Food system resilience (FSR)

Based on the theoretical analysis and relevant literature [9, 13, 14, 32], we selected 17 indicators to construct the resilience index for the food system. The system is constructed from three dimensions: resistance, adaptability, and changeability (refer to Table 1). To avoid measurement bias caused by inconsistent indicators, this paper uses the “entropy method,” a statistical technique that standardizes indicators by assigning weights based on the information content or variability of each indicator, to standardize indicators and measure food system resilience objectively by assigning weights to each indicator. This is then integrated into a composite index, drawing on the research of Zhu Xi’an et al. [33].

**Table 1:** Index system of food system resilience.

Primary index	Secondary index	Index calculation method and unit	Property	Weight
Capacity for resist	Cultivated land per capita	Cultivated land area/number of persons employed in the primary sector (ha/person)	+	0.102
	Labor force level	Number of people employed in primary industry/total employment (%)	+	0.037
	Level of fixed asset investment	Investment in agricultural fixed assets / Gross regional product (%)	+	0.054
	Grain production per capita	Total grain production/total number of people in the province (kg/person)	+	0.082
	Food production price index	Food production prices of the current/previous year (previous year = 100)	-	0.016
	Grain production level	Grain production/grain sown area (t/ha)	+	0.022
Capacity for capacity	Intensity of pesticide use	Pesticide use / area sown with crops (t/ha)	-	0.001
	Degree of disaster	Crop damage area / crop sown area (%)	-	0.005
	Fertilizer use intensity	Amount of agricultural fertilizer applied (pure) / area sown with crops (t/ha)	-	0.016
	Intensity of agricultural film use	Agricultural plastic film use/crop sown area (t/ha)	-	0.008
	Replanting index	Grain sown area/cultivated area (%)	+	0.028
	Agricultural value add index	Agricultural value added in the current/previous year (previous year = 100)	+	0.005
	Per capita rural electricity consumption	Rural electricity consumption/rural population (10 thousand* kW·h/person)	+	0.297
Capacity for change	Degree of mechanization	Total power of agricultural machinery/area sown with crops (10*kW)/ha	+	0.047
	Level of agricultural development	Gross agricultural output value / gross regional product (%)	+	0.052
	Level of Expenditure on Agricultural Research	Local fiscal expenditure on science and technology* gross agricultural output value/gross regional product (CNY billion)	+	0.088
	Level of agricultural technicians	Persons obtaining national vocational qualification certificates in the grain industry/number of employees in primary industry (%)	+	0.138

#### 3.2.2. Independent variable: Level of digital economy (Dig)

The literature on constructing a digital economy index system primarily focuses on digital economy carriers, industrial digitization, and digital industrialization [34–36]. This paper selects 19 indicators for measurement (Table 2). The steps for measuring the comprehensive index of the digital economy level using the entropy value method are consistent with those for measuring the food system resilience index.

**Table 2:** Index system of the digital economy.

Primary index	Secondary index	Index calculation method and unit	Property	Weight
Carrier of digital economy	Cell phone penetration	Number of cell phone subscribers per 100 people (households/100 people)	+	0.011
	Internet penetration	Number of Internet users as a proportion of resident population (%)	+	0.011
	Breadth of information transmission	Density of fiber-optic cable lines (km/km2)	+	0.060
	Signal coverage breadth	Density of cell phone base stations (pcs/km2)	+	0.077
	Internet broadband infrastructure	Density of Internet broadband access ports (pcs/km2)	+	0.081
	Strength of investment in digital services	Per capita fixed asset investment in information transmission, computer services and software industry (CNY/person)	+	0.027
Digital industrialization	Development level of post and telecommunications industry	Total telecommunication business per capita (CNY/person)	+	0.050
		Total postal services per capita (CNY/person)	+	0.074
		Volume of express delivery (ten thousand pieces)	+	0.114
	Development level of electronic information manufacturing industry	Revenue of electronic information manufacturing industry (CNY)	+	0.094
		Number of enterprises in electronic information manufacturing industry (nos.)	+	0.092
	Software and information technology service industry	Software business revenue (CNY)	+	0.090
		Number of employees in information transmission, software and information technology services (ten thousand)	+	0.054
Digitalization of industries	Development level of enterprise digitization	Number of websites owned by enterprises (number)	+	0.044
		Share of enterprises with e-commerce transaction activities (%)	+	0.016
		E-commerce transaction volume (CNY billion)	+	0.069
	Development level of digital inclusive finance	Digital finance coverage breadth index	+	0.013
		Depth of digital finance usage index	+	0.012
		Digital finance digitization degree	+	0.010

#### 3.2.3. Mediating variable: Food industry agglomeration (Agg)

Drawing on Han Haibin et al. (2023) and Yang Xiuyu et al. (2023) [37, 38], the degree of food industry agglomeration is measured via locational entropy, which overcomes the influence of regional scale differences and other effects, and it measures the degree of agricultural agglomeration more accurately with the following formula:

$$Agg_{it}=\left(A_{it}⁄G_{it}\right)/\left(A_{t}⁄G_{t}\right)$$ (6)

Eq (6) defines *Agg*_*it*_ as the degree of food industry agglomeration in province i at time t, which is proportional to its value. *A*_*it*_ and *G*_*it*_ represent the total output value of agriculture, forestry, livestock, and fishery, and the gross regional product of region i in year t, respectively. *A*_*t*_ and *G*_*t*_ represent the total output value of agriculture, forestry, livestock, and fishery and the gross domestic product of the entire country in year t, respectively.

#### 3.2.4. Control variables

To ensure the resilience of the food system, the following control variables are primarily considered to control the impact of other variables:

1. The level of informatization is expressed by the ratio of the total amount of post and telecommunication services to GDP.
2. Employment density is represented by the ratio of the number of employees in the primary industry to the area of administrative divisions.
3. Foreign direct investment is expressed as the ratio of foreign direct investment to GDP after multiplying the current USD/CNY exchange rate.
4. The level of development in the technology market can be measured using the ratio of technology market turnover to GDP.
5. The degree of industrialization can be measured using the ratio of the industrial added value to GDP.
6. The degree of openness to the outside world can be measured using the ratio of the value of imports and exports of goods, multiplied by the USD/CNY exchange rate of the current year, to GDP.

### 3.3. Data sources

Considering the availability of data, this paper uses 30 provinces in China (except Tibet, Hong Kong, Macao, and Taiwan) from 2011 to 2021 as the research object, and the data are mainly obtained from the China Rural Statistical Yearbook, China Statistical Yearbook, EPS database, and statistical yearbooks and statistical bulletins of the corresponding years in each province and city. Linear interpolation is used to add some missing values. The descriptive statistics of each variable are shown in Table 3.

**Table 3:** Descriptive statistics of variables.

Variable	Symbol	Mean	SD	Min	Max
Resilience of food system	FSR	0.195	0.058	0.114	0.463
Digital economy	Dig	0.119	0.108	0.010	0.647
Industrial agglomeration	Agg	1.185	0.623	0.049	3.467
Informatization level	Inf	0.060	0.055	0.014	0.290
Employment density	Emp	0.026	0.039	0.000	0.217
Foreign direct investment	For	0.019	0.015	0.000	0.080
Technology market development level	Tec	0.016	0.029	0.000	0.175
Degree of industrialization	Ind	0.321	0.082	0.101	0.556
Degree of opening up	Open	0.265	0.291	0.008	1.548

## 4. Results analysis and discussion

### 4.1. Spatial and temporal characterization of food system resilience

**Fig 1:**
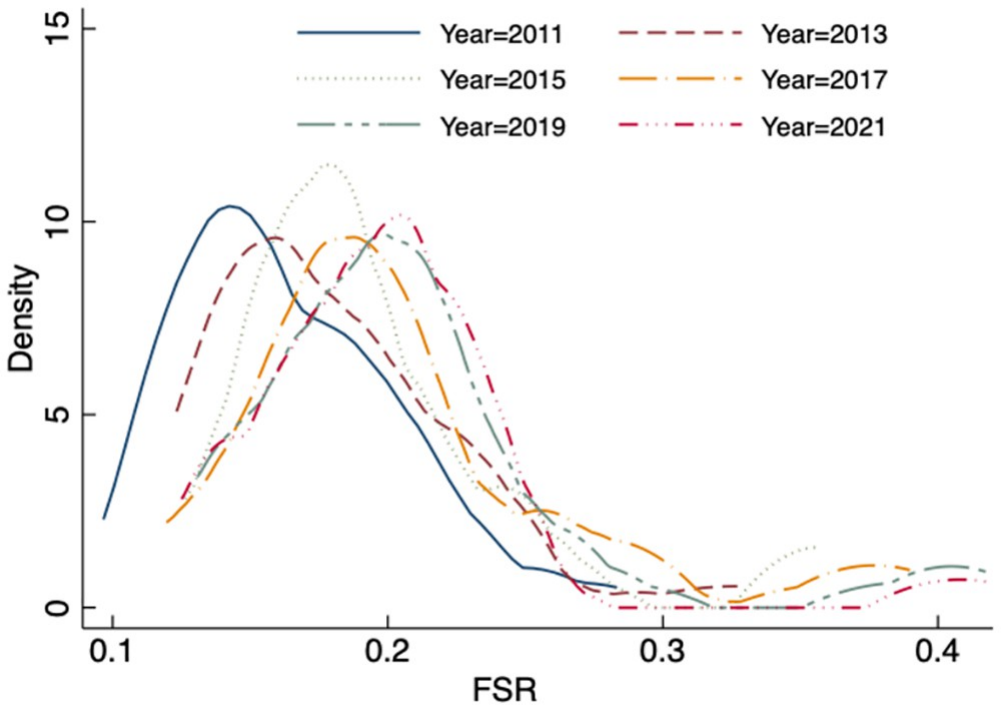
Spatial and temporal characteristics of the resilience of China’s food system.

This paper examines the spatial and temporal characteristics of China’s food system resilience by estimating the kernel density map through plotting. Fig 1 shows that the center of kernel density moves to the right each year, indicating an improvement in the resilience of the food system and effective food security guarantee. The distribution curves of each year indicate that the resilience of the food system in most regions of China is still at a middle to lower level. The curves also show a unipolar phenomenon during the examination period. These findings suggest that the food system in China needs improvement. Additionally, the curves for each year exhibit a clear “right trailing” pattern, suggesting that the resilience of the food system during the study period is unipolar. Overall, China’s food system has significantly improved in recent years. However, there are still substantial variations in the resilience levels across different regions, with a larger proportion of regions having lower resilience levels. Therefore, there is a need to strengthen the resilience level of the food system.

### 4.2. Analysis of baseline regression results

Prior to conducting empirical analyses, the variables were assessed for multicollinearity. The results indicate that Max-VIF is 3.39, which is significantly lower than the threshold of 10, thus indicating the absence of multicollinearity. To circumvent the peril of "pseudo-regression" arising from non-stationary data, an IPS unit root test was administered, ensuring that all selected variables exhibited stationarity at a significance level exceeding 10%. Given the panel data nature of the variables, we subsequently assessed the suitability of mixed regression, random effects, and fixed effects models. The F-test decisively favored fixed effects over mixed regression, with Prob > F = 0.0000. Furthermore, the Hausman test indicated a preference for the fixed effects model over the random effects model, as evidenced by Prob > chi2 = 0.0299. Consequently, this study adopts the bidirectional fixed effects model for regression analysis.

The impact of the digital economy on the resilience of the food system was analyzed using the baseline regression model. The results are presented in Table 4, with columns (1) to (4) showing the results of no fixing, province fixing, year fixing, and province and year fixing after the introduction of the control variables, respectively. The results indicate that the regression coefficients of the digital economy are all positive and pass the significance test at the 10% level when the province and year effects are fixed. This suggests that the digital economy significantly enhances the level of food system resilience. Possible reasons for this include the use of digital technologies such as big data and intelligent control to achieve precision planting, intelligent fertilizer application, and automated irrigation. This enhances food resistance through precision and intelligent agriculture. It promotes the rational use of natural resources, such as land and water, and supports sustainable food production. Additionally, it enhances the adaptive capacity of the food system. The promotion of digital transformation in the food industry chain encourages food operating entities to accelerate technological progress, improve technological efficiency, and ultimately enhance the resilience of the food system [14].

**Table 4:** Baseline regression result.

Variable	(1) FSR	(2) FSR	(3) FSR	(4) FSR
Dig	0.164*** (0.028)	0.102**(0.047)	0.121***(0.038)	0.111*(0.063)
Inf	0.003 (0.056)	0.077**(0.032)	-0.147(0.115)	0.255***(0.084)
Emp	1.012*** (0.108)	2.326***(0.641)	0.987***(0.119)	2.384***(0.637)
For	-0.336*(0.184)	-0.431***(0.131)	-0.362*(0.192)	-0.462***(0.140)
Tec	-0.240***(0.091)	0.444**(0.193)	-0.275***(0.092)	0.462**(0.192)
Ind	-0.081*(0.045)	-0.092**(0.044)	-0.070(0.044)	-0.068(0.050(
Open	-0.112***(0.012)	-0.022(0.024)	-0.096***(0.017)	-0.016(0.025)
Year	No	No	Yes	Yes
province	No	Yes	No	Yes
Sample	330	330	330	330
R^2^	0.293	0.863	0.302	0.872

Note: Robust standard error in brackets: * * *, * *, and * indicate significant at 1%, 5%, and 10% levels, respectively. The same below.

When controlling for province and year effects, the level of informatization showed a significant positive impact on the resilience of the food system. This may be due to its ability to facilitate information sharing, reduce information asymmetry, improve coordination within the food system, enhance its ability to cope with external shocks, and ultimately increase its resilience [39]. Additionally, the level of employment density also had a significant positive impact on the resilience of the food system at the 1% level. The resilience of the food system was found to have a statistically significant positive effect at the 1% level in areas with high employment density. This may be due to the fact that such areas are conducive to the better management of food producers and operators, improve the quality and skill levels of food producers and operators, and have a better ability to cope with external shocks and risks in the food industry [40]. Foreign direct investment was found to have a statistically significant negative effect on the resilience of the food system at the 1% level. Foreign direct investment may lead to increased market competition for domestic food production operators, resulting in the use of agricultural chemicals and other means of production to enhance market competitiveness. This may not be conducive to the stabilization of food system resilience [41]. Statistical analyses show that technological market development has a positive and significant impact on food system resilience at the 5% level [42]. This may be due to the fact that technological market development enables food production operators to quickly adopt new technologies, modes, and business models, which in turn improves production efficiency, product quality, and added value in the food industry. As a result, the resilience of the food system is enhanced.

### 4.3. Robustness test

In order to verify the reliability of the findings, a robustness test was conducted (Table 5).

**Table 5:** Robustness test.

Variable	(1) FSR	(2) FSR	(3) FSR
Dig	0.214* (0.120)	0.127* (0.069)	— —
Dig#	— —	— —	0.181** (0.084)
Control variable	Yes	Yes	Yes
Year	Yes	Yes	Yes
Province	Yes	Yes	Yes
Sample	120	270	330
R^2^	0.959	0.886	0.865

#### 4.3.1. Adjustment of the sample period

The sample period is adjusted to 2018–2021 and 2011–2019, respectively. The reasons are as follows: In 2017, the digital economy was written in the government work report for the first time, and the development of the digital economy opened a new chapter; this policy factor may affect research conclusions, and only the sample after the policy’s release is retained for analyses. The regression results are shown in column (1). In 2020, there was a large impact of the New Crown Epidemic on both the development of the digital economy and food production, and in order to exclude the impact of this major environmental change, only the samples before 2020 were analyzed. The regression results are shown in column (2). Columns (1) and (2) show that after adjusting for the sample period separately, the digital economy still passes a positive significance test at the 10% level for food system resilience.

#### 4.3.2. Replacement of core explanatory variables

The entropy value method was used in the previous section to measure the composite index, and the CRITIC and entropy value methods are both objective weighting methods. The "CRITIC" method, in simple terms, is a multi-criteria decision analysis tool that assigns weights to indicators based on both the contrast strength (how well the indicators discriminate among evaluation units) and the conflict among criteria (how much correlation exists between indicators), thus providing an objective evaluation framework. Therefore, the CRITIC method avoids the problem of weighting when dealing with the comprehensive evaluation of multiple indicators, but it also takes into account the effect of correlations on the weight of indicators and eliminates the effect of correlation indicators on the weight during weighting [43]. Using this method to re-measure the level of digital economy development and define the new variable as “Dig#”, the regression results are shown in column (3) of Table 5. The results show that the estimation of the digital economy with respect to the resilience of food systems is still significant after using the CRITIC method to measure the digital economy. This indicates that the development of the digital economy increases the level of resilience of the food system.

### 4.4. Endogeneity test

In general, regions that exhibit robust resilience within their food systems generally possess more advanced digital infrastructure, suggesting that digital economy development confers a "first-mover advantage." This implies a potential bidirectional causality between the digital economy and food system resilience, making it challenging to conclusively ascertain whether the latter’s enhancement stems solely from the former’s progression. Consequently, this study employs the panel instrumental variable approach to address the issue of endogeneity. Therefore, in this study, based on the experience of Huang Qunhui et al. [44] and the availability of data, the number of landline phones at the end of 1990 (in 10,000 households) in each region is selected as the instrumental variable, and a regression test is conducted using the two-stage least squares method.

The instrumental variable selection in this study is grounded on several pivotal considerations: Firstly, the ubiquitous adoption of Internet technology forms the cornerstone for digital economy growth. Tracing back, the advent of Internet technology can be attributed to the widespread use of fixed-line telephones. Regions boasting high penetration rates of fixed-line telephones also exhibit a relatively advanced digital economy, suggesting a strong correlation between digital economic progress and fixed-line penetration. Specifically, the digital economy’s core momentum stems from Internet advancements, which can be viewed as an extension of fixed-line telephony’s proliferation. This implies that the prevalence of fixed-line telephones constitutes a vital aspect of digital economy infrastructure, fulfilling the relevance criterion. Conversely, in the era of rapid communication technology evolution, traditional landlines contribute minimally to the resilience of contemporary food systems, thereby meeting the exclusivity requirement [45]. It is worth noting that the number of landline telephones at the end of 1990 comprises cross-sectional data, while the sample in this paper comprises balanced panel data. Therefore, based on Nunn and Qian [46], the cross-multiplier term between the number of Internet access instances in the previous year and the number of landline telephones at the end of 1990 is constructed as an instrumental variable (IV) for the digital economy index of the current period. The estimation results are presented in Table 6. The results in column (1) indicate that the instrumental variable has a significant positive effect on the digital economy, and the results in column (2) show that after applying the two-stage least squares method to mitigate the endogeneity problem, the digital economy still passes the positive significance test on the resilience of the food system and is significant at the 5% level, which is basically the same as the results of the benchmark regression. In addition, the results of the weak instrumental variable test show that the F-statistic is 241.435, which is significantly greater than the critical value of 10; therefore, there is no weak instrumental variable problem. Accordingly, this indicates that the development of the digital economy determines the improvement of the resilience of the food system, which verifies hypothesis H1.

**Table 6:** Results of the endogeneity test.

Variable	Instrumental variable method	
	(1) Dig	(2) FSR
IV	6.38e-08*** (4.12e-09)	— —
Dig	— —	0.103** (0.051)
Control variable	Yes	Yes
Year	Yes	Yes
Province	Yes	Yes
F-value		241.435
Sample	330	330
R^2^	0.955	0.895

## 4.5. Mechanism of action test

To further test the mediating effect of food industry agglomeration on the impact of the digital economy on food system resilience, the mediating effect model constructed in the previous section is used for validation (Table 7). As observed in Table 7, in column (1), the digital economy on food industry agglomeration passed the positive significance test at the 1% level, indicating that the development of the digital economy promotes the food industry’s agglomeration level. In column (2), after the introduction of the digital economy variable, both the digital economy and food industry agglomeration on food system resilience passed the positive significance test at the 1% level, indicating that food industry agglomeration enhances the level of food system resilience. Accordingly, this indicates that the digital economy can enhance the resilience level of the food system by promoting the degree of food industry agglomeration. Food industry agglomeration plays a mediating effect on the impact of the digital economy on the resilience of the food system.

**Table 7:** Results of the endogeneity test.

Variable	(1) Agg	(2) FSR
Dig	1.912***(0.474)	0.235***(0.066)
Agg		0.065***(0.008)
Control variable	Yes	Yes
Year	Yes	Yes
province	Yes	Yes
Sample	330	330
R2	0.955	0.895

In order to test the reliability of the above mediated effect results, the self-sampling test method (bootstrap) was used to conduct a robustness test of the above results, and the number of bootstrap replicate samples was set at 500. The results are shown in Table 8. The results show that the coefficients of the indirect and direct effects are positively significant at the 1% level, and the 95% confidence interval does not include 0, which is basically consistent with the above conclusion. The mediating effect of the agglomeration of the food industry is again verified. Hypothesis H2 is therefore confirmed.

**Table 8:** Tests the mediation effect based on the bootstrap method.

Influence type	Coefficient	SD	P-value	95% confidence interval	
Indirect effect	0.068	0.019	0.000	0.036	0.115
Direct effect	0.232	0.036	0.003	0.174	0.316

### 4.6. Analysis of spatial spillover effects

#### 4.6.1. Spatial autocorrelation test

Before carrying out the spatial econometric model, this paper adopts the global Moran’s index (Moran’s *I*) to calculate the spatial correlation between digital economy and food system resilience in each region under the spatial proximity matrix, and the results are shown in Table 9. The results show that Moran’s index of digital economy and food system resilience for each year passed the positive significance test, indicating that there is an obvious positive spatial correlation between the digital economy and food system resilience in our region during the study period.

**Table 9:** Spatial autocorrelation test.

Year	Digital economy		Food system resilience	
	Moran’s I	Z-value	Moran’s I	Z-value
2011	0.261**	2.488	0.463***	4.081
2012	0.300***	2.813	0.441***	3.951
2013	0.266**	2.536	0.408***	3.781
2014	0.262**	2.503	0.433***	4.072
2015	0.281***	2.660	0.299***	2.890
2016	0.272**	2.592	0.268**	2.583
2017	0.251**	2.421	0.240**	2.345
2018	0.227**	2.240	0.153*	1.695
2019	0.216**	2.160	0.269***	2.699
2020	0.217**	2.162	0.233**	2.415
2021	0.223**	2.221	0.163*	1.835

**Fig 2:**
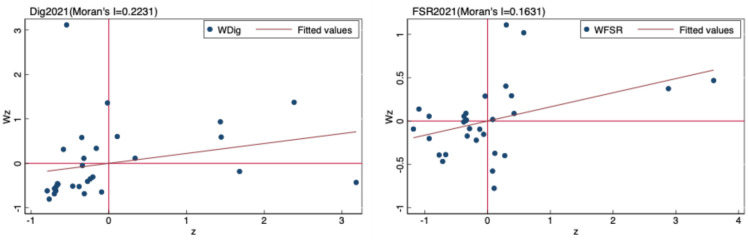
Moran’s *I* scatter plot of the digital economy and food system resilience in 2021.

We then plotted a Moran scatterplot of the digital economy and food system resilience in 2021 using Stata16 software (Fig 2). The results show that most areas are distributed in the high—high and low—low quadrants, indicating the spatial clustering of the digital economy and food system resilience and initially validating the spillover effect between the two.

#### 4.6.2. Spatial spillover effects

Before conducting empirical analyses, it is necessary to determine which spatial econometric model to select, and the results of the LM, LR, and Hausman tests show that the spatial Durbin model is superior to the spatial lag model and the spatial error model (Table 10). The Hausman test value is 18.49 (*P* = 0.0299), indicating that the fixed effect model should be chosen.

**Table 10:** Test results of spatial econometric model.

Model	LM	Robust LM	Wald	LR
SLM	5.270*	2.992*	7.542**	14.687**
SEM	33.397***	32.118***	8.100**	16.212***

In order to compare the test results, the regression results of the spatial Durbin model (SDM) and the spatial autoregressive model (SAR) were reported simultaneously, following Yi Enwen (2023) [45] (Table 11). The results show that the effect of the digital economy on local food system resilience (direct effect), the effect of the local digital economy on food system resilience in neighboring regions (indirect effect), and the total effect (the sum of direct and indirect effects) all passed the positive significance test. On the one hand, the development of the digital economy can promote the digital transformation of the local food industry chain. It can improve the efficiency and resilience of all aspects of food production, circulation, and consumption; for example, the digital economy can help food production operators more accurately manage arable land and optimize the structure of grain cultivation, etc. Moreover, the optimization of logistics can be promoted. On the other hand, the development of the local digital economy can attract the inflow of talent and resources from neighboring regions and promote the exchange and sharing of digital technology, the construction of digital infrastructure, and the development of digital industrialization in neighboring regions, which in turn enhance the resilience of the food system in neighboring regions. Therefore, hypothesis H3 is supported.

**Table 11:** Test results of the spatial spillover effect.

Variable	SDM	SAR
Digital economy	0.371***(0.037)	0.381***(0.035)
W×Dig	0.060*(0.044)	
rho	0.177**(0.083)	0.211***(0.076)
sigma2_e	0.000***(0.000)	0.000***(0.000)
Direct effect	0.378***(0.039)	0.387***(0.037)
Indirect effect	0.151**(0.071)	0.103**(0.046)
Total effect	0.530***(0.058)	0.491***(0.065)
Control variable	Yes	Yes
Year	Yes	Yes
Province	Yes	Yes
Sample	330	330
Within R^2^	0.366	0.458

## 5. Conclusions and policy recommendations

### 5.1. Conclusions

This study takes the panel data of 30 provinces in China from 2011 to 2021 as a sample; constructs an index system of China’s digital economy and food system resilience; draws a kernel density map to analyze the spatial and temporal differences in the characteristics of China’s food system resilience; uses the double fixed-effects model to test the impact effect of the digital economy on food system resilience; and applies the mediating effects model to clarify the transmission mechanisms of the digital economy on food systems through the industry. Using the mediating effects model, we clarified the transmission mechanism of agglomeration in the digital economy with respect to food system resilience and used the spatial measurement model to explore the spatial spillover effect of the digital economy on food system resilience. The results of the study show that, firstly, China’s current food system resilience level in the time series shows a stable growth trend, but with respect to the spatial difference, it is obvious that the level of food system resilience still needs to be strengthened as the region’s low levels accounted for a large proportion of the narrowing of regional differences. Second, the digital economy plays an important role in promoting the resilience of the food system, and this conclusion is still valid after considering the endogeneity and robustness test. Therefore, in the process of promoting the resilience of the food system driven by the digital economy, the “digital dividend” effect plays an important role, which provides empirical evidence for ensuring China’s food security and comprehensively promoting the rural revitalization strategy. Third, promoting industrial agglomeration is an inherent mechanism of the digital economy to enhance the resilience of the food system. Driven by the digital economy, resource elements in the agglomeration area will be revitalized, and production capacities can be released to promote the digital transformation of the food industry, which in turn further enhances the resilience of the food system. Fourth, there is a positive spatial spillover effect: i.e., the development of the digital economy at the provincial and national food system resilience levels has an important role in promoting the resilience of the food system in neighboring provinces and cities; simultaneously, there is a significant positive impact on the resilience of the food system.

### 5.2. Policy recommendations

Firstly, we propose enhancing infrastructure construction and fostering digital village development. To bridge the gap in rural digital infrastructure, prioritize support for the digital transformation of mobile communication base stations and terminals. Expedite the deployment of efficient, high-speed mobile networks, particularly 5G coverage in remote rural areas. Further explore 5G applications to refine data perception, collection, filtering, and transmission, establishing a digital information highway for the grain industry and solidifying the digital economy’s foundation. Additionally, elevate farmers’ digital literacy and IT application skills, advocating the principle of "integrating information technology with agricultural machinery and agronomy for smart agriculture advancement." Strengthen ideologies by enhancing awareness of smart agriculture and practical food production skills, conducting digital agriculture training, fostering a mindset for food quality development, cultivating digital agricultural literacy, and accelerating new vocational digital agriculture education.

Secondly, reinforce industrial agglomeration to boost food quality and efficiency. Industrial agglomeration is vital for fostering resilience in the digital economy-driven food system. Improve market-oriented development to attract upstream and downstream clusters. Foster technological innovation through large-scale, region-specific projects, and increase R&D investments and talent development to encourage agricultural professionals’ grassroots engagement. Create a favorable, market-driven, and legal international business environment. Avoid blindly pursuing foreign investments, focusing instead on transitioning from agricultural quantity to quality. Multi-faceted efforts will elevate industrial development’s quality and efficiency, gradually enhancing food system resilience.

Lastly, promote regionally integrated development to secure food safety. Recognizing the interconnectedness of regional food system resilience, tailor food development strategies to local conditions. Emphasize enhancing specialty food quality, building local brands, and innovating business models. Strengthen inter-city food industry cooperation, unifying the national market, and transcending local barriers. Facilitate effective "hardware" and "software" integration across regions, promoting platform development, industrial integration, public services, and talent exchanges within administrative zones.

### 5.3. Main limitations

This paper is dedicated to exploring the relationship between the digital economy and food system resilience, and empirical methods have been used to carry out useful explorations. However, due to datum limitations and the author’s lack of experience, there is still room for improvement and exploration. Firstly, although the scientificity and completeness of the indicators for measuring the digital economy and food system resilience have been taken into account as much as possible, the complexity of food production activities and the availability of data inevitably lead to the omission of variables, which in turn imparts a certain degree of bias in the analysis’s results. Secondly, although the scale of the study has a certain degree of depth and the data of the study are relatively fine, gaps still exist compared to studies carried out at the county or township scale; further in-depth research will be carried out with respect to these factors.

## Supporting information

S1 Data The processed data. (XLSX)

S2 Data Data before processing. (XLSX)
