# Supplementary material for: The impact of the digital economy on food system resilience: Evidence from China
Source: PLoS One. 2024 Oct 24;19(10):e0311689. doi: 10.1371/journal.pone.0311689 (PMC11500886; doi:10.1371/journal.pone.0311689)
Supplement: Attachment — Submitted filename: PONE-D-24-27750.pdf [file pone.0311689.s003.pdf]

PLOS ONE

The Impact of the Digital Economy on Food System Resilience: Evidence from China

--Manuscript Draft--

**Manuscript Number:**	PONE-D-24-27750
**Article Type:**	Research Article
**Full Title:**	The Impact of the Digital Economy on Food System Resilience: Evidence from China
**Short Title:**	The Impact of the Digital Economy on Food System Resilience
**Corresponding Author:**	Zhen Wang<br>Sichuan Agricultural University<br>ChengDu, CHINA
**Keywords:**	food systems resilience; digital economy; industrial agglomeration; mediating effect; spatial spillover effect
**Abstract:**	Promoting a digital economy to empower food system resilience is particularly important for food security. Based on the panel data of 30 provinces in China from 2011 to 2021, this paper empirically examines the direct effect, the mechanism of action, and the spatial spillover effect of the resilience of a digital economy-enabled food system using a double fixed-effects model, mediated-effects model, and spatial econometric model. The results show the following: (1) The resilience of China's urban food system shows obvious spatial differences, but the overall trend in improvement requires attention. (2) The development of a digital economy has a facilitating effect on the level of resilience of the food system, and industrial agglomeration induces an intermediary effect. (3) The digital economy has a significant positive spatial spillover effect on the resilience of the food system; i.e., the digital economy can improve the resilience of the food system in the region and the neighboring regions. Accordingly, policy recommendations have been put forward to improve infrastructure construction and promote the development of digital villages; strengthen the construction of industrial agglomerations and promote the enhancement of the quality and efficiency of industries; and promote the development of regional linkages and build a solid foundation for food security.
**Order of Authors:**	Zhu Zheng
	Zhen Wang
	Bin Liu
**Additional Information:**	
**Question**	**Response**
**Financial Disclosure**<br><br>Enter a financial disclosure statement that describes the sources of funding for the work included in this submission. Review the submission guidelines for detailed requirements. View published research articles from *PLOS ONE* for specific examples.<br><br>This statement is required for submission and **will appear in the published article** if the submission is accepted. Please make sure it is accurate.	Funding: This research was funded by the National Natural Science Foundation of China (grant no. 71563016), Key Research Base Project of Humanities and Social Sciences in colleges and uni-versities of Jiangxi Province (JD21080), the Jiangxi Postgraduate Innovation Special Fund Project (YC2022-s415), and the Jiangxi Agricultural University School of Economics and Management 2022 Postgraduate Innovation Special Fund Project (grant no. JG2022008).

Powered by Editorial Manager® and ProduXion Manager® from Aries Systems Corporation

**Unfunded studies**<br>Enter: *The author(s) received no specific funding for this work.*<br><br>**Funded studies**<br>Enter a statement with the following details:<br>• Initials of the authors who received each award<br>• Grant numbers awarded to each author<br>• The full name of each funder<br>• URL of each funder website<br>• Did the sponsors or funders play any role in the study design, data collection and analysis, decision to publish, or preparation of the manuscript?<br>• **NO** - Include this sentence at the end of your statement: *The funders had no role in study design, data collection and analysis, decision to publish, or preparation of the manuscript.*<br>• **YES** - Specify the role(s) played.<br><br>* typeset	
**Competing Interests**<br><br>Use the instructions below to enter a competing interest statement for this submission. On behalf of all authors, disclose any competing interests that could be perceived to bias this work—acknowledging all financial support and any other relevant financial or non-financial competing interests.<br><br>This statement is required for submission and **will appear in the published article** if the submission is accepted. Please make sure it is accurate and that any funding sources listed in your Funding Information later in the submission form are also declared in your Financial Disclosure statement.<br><br>View published research articles from *PLOS ONE* for specific examples.	The authors have declared that no competing interests exist.

**NO authors have competing interests**<br><br>Enter: *The authors have declared that no competing interests exist.*<br><br>**Authors with competing interests**<br><br>Enter competing interest details beginning with this statement:<br><br>*I have read the journal's policy and the authors of this manuscript have the following competing interests: [insert competing interests here]*<br><br>* typeset	
**Ethics Statement**<br><br>Enter an ethics statement for this submission. This statement is required if the study involved:<br><br>• Human participants<br>• Human specimens or tissue<br>• Vertebrate animals or cephalopods<br>• Vertebrate embryos or tissues<br>• Field research<br><br>Write "N/A" if the submission does not require an ethics statement.<br><br>General guidance is provided below. Consult the submission guidelines for detailed instructions. **Make sure that all information entered here is included in the Methods section of the manuscript.**	N/A

**Format for specific study types**<br><br>**Human Subject Research (involving human participants and/or tissue)**<br>• Give the name of the institutional review board or ethics committee that approved the study<br>• Include the approval number and/or a statement indicating approval of this research<br>• Indicate the form of consent obtained (written/oral) or the reason that consent was not obtained (e.g. the data were analyzed anonymously)<br><br>**Animal Research (involving vertebrate animals, embryos or tissues)**<br>• Provide the name of the Institutional Animal Care and Use Committee (IACUC) or other relevant ethics board that reviewed the study protocol, and indicate whether they approved this research or granted a formal waiver of ethical approval<br>• Include an approval number if one was obtained<br>• If the study involved *non-human primates*, add *additional details* about animal welfare and steps taken to ameliorate suffering<br>• If anesthesia, euthanasia, or any kind of animal sacrifice is part of the study, include briefly which substances and/or methods were applied<br><br>**Field Research**<br><br>Include the following details if this study involves the collection of plant, animal, or other materials from a natural setting:<br>• Field permit number<br>• Name of the institution or relevant body that granted permission	
**Data Availability**<br><br>Authors are required to make all data underlying the findings described fully available, without restriction, and from the time of publication. PLOS allows rare exceptions to address legal and ethical concerns. See the PLOS Data Policy and FAQ for detailed information.	Yes - all data are fully available without restriction

A Data Availability Statement describing where the data can be found is required at submission. Your answers to this question constitute the Data Availability Statement and **will be published in the article**, if accepted.<br><br>**Important:** Stating 'data available on request from the author' is not sufficient. If your data are only available upon request, select 'No' for the first question and explain your exceptional situation in the text box.<br><br>Do the authors confirm that all data underlying the findings described in their manuscript are fully available without restriction?	
**Describe where the data may be found in full sentences. If you are copying our sample text, replace any instances of XXX with the appropriate details.**<br><br>• If the data are **held or will be held in a public repository**, include URLs, accession numbers or DOIs. If this information will only be available after acceptance, indicate this by ticking the box below. For example: *All XXX files are available from the XXX database (accession number(s) XXX, XXX.).*<br>• If the data are all contained **within the manuscript and/or Supporting Information files**, enter the following: *All relevant data are within the manuscript and its Supporting Information files.*<br>• If neither of these applies but you are able to provide **details of access elsewhere**, with or without limitations, please do so. For example:<br><br>*Data cannot be shared publicly because of [XXX]. Data are available from the XXX Institutional Data Access / Ethics Committee (contact via XXX) for researchers who meet the criteria for access to confidential data.*<br><br>*The data underlying the results presented in the study are available from (include the name of the third party*	If you need any data, you can contact any of the authors to request it.

Powered by Editorial Manager® and ProduXion Manager® from Aries Systems Corporation

and contact information or URL).<br>• This text is appropriate if the data are owned by a third party and authors do not have permission to share the data.<br><br>* typeset	
Additional data availability information:	Tick here if your circumstances are not covered by the questions above and you need the journal's help to make your data available.

Manuscript Click here to access/download;Manuscript;Manuscript.pdf

Article

The Impact of the Digital Economy on Food System Resilience: Evidence from China

Zhu Zheng[1,2], Wang Zhen [3]*, Liu Bin [2]*

[1] Jiangxi Daily, Nanchang 330006, China; 13970864503@163.com
[2] School of Economics & Management, Jiangxi Agricultural University, Nanchang 330045, China;
[3] School of Economics, Sichuan Agricultural University, Chengdu 611130, China;
* Correspondence: 18870478773@163.com(Z.W.); liubin@jxau.edu.cn (B.L.)

Abstract: Promoting a digital economy to empower food system resilience is particularly important for food security. Based on the panel data of 30 provinces in China from 2011 to 2021, this paper empirically examines the direct effect, the mechanism of action, and the spatial spillover effect of the resilience of a digital economy-enabled food system using a double fixed-effects model, mediated-effects model, and spatial econometric model. The results show the following: (1) The resilience of China's urban food system shows obvious spatial differences, but the overall trend in improvement requires attention. (2) The development of a digital economy has a facilitating effect on the level of resilience of the food system, and industrial agglomeration induces an intermediary effect. (3) The digital economy has a significant positive spatial spillover effect on the resilience of the food system; i.e., the digital economy can improve the resilience of the food system in the region and the neighboring regions. Accordingly, policy recommendations have been put forward to improve infrastructure construction and promote the development of digital villages; strengthen the construction of industrial agglomerations and promote the enhancement of the quality and efficiency of industries; and promote the development of regional linkages and build a solid foundation for food security.

Keywords: food systems resilience; digital economy; industrial agglomeration; mediating effect; spatial spillover effect

1. Introduction

Food security is "the most important thing in the country", and its major strategic role has a bearing on economic development and social stability; it also serves as an important foundation for national security. Since 2015, China has achieved sustained growth in total grain output, reaching a new level of 650 million tons. [illegible]2023, it is projected to reach over 690 million tons, despite possessing less than 10% of the world's arable land to feed nearly 20% of the world's population. This is a remarkable feat and provides a powerful answer to the question of "who will feed China" [1]. Ensuring China's food security remains an urgent priority despite continuous improvements in the country's comprehensive food production efficiency. This is due to multiple constraints, such as domestic shortages of arable land, water, and other resources, and international geopolitical conflicts and a global economic slowdown [2]. The "2023 State of Food Security and Nutrition in the World" report by the United Nations highlights the need to enhance resilience to tackle food crises. Similarly, "Central Document No. 1 in 2023" emphasizes the importance of strengthening agriculture as a means of building a resilient industrial sector and a strong agricultural nation. Promoting the construction of a resilient food system is a path toward resisting and quickly recovering from risky shocks, shifting to a new growth path, achieving adaptive food development, and guaranteeing a balance between food production and supply while eliminating the root causes of food insecurity [3]. However, the food

industry is often associated with low-quality and inefficient production processes, making it challenging to enhance system resilience solely through its own efforts. In recent years, the digital economy has gradually penetrated all areas of the national economy and social development through cloud computing, big data, artificial intelligence, and other core high and new technologies. Its deep integration with agriculture provides new opportunities and challenges for enhancing the resilience of the food system. Empowering the food system with the digital economy will become an important direction for the future development of agriculture [4]. Furthermore, expediting the growth of the digital economy can strengthen the resilience of the food system. It is important to note that industrial agglomeration plays a crucial role in this process [5]. By leveraging the economic scale's externality, the specialization of the division of labor, and knowledge spillover, industrial agglomeration can enhance the efficiency of food production, improve the industry’s competitiveness and risk management capabilities, and ultimately bolster the resilience of food systems [6]. Therefore, conducting an in-depth investigation into the impact and internal mechanisms of the digital economy on the resilience of the food system is of great theoretical and practical significance in guaranteeing national food security and comprehensively promoting rural revitalization.

There are three main types of literature related to the research theme of this paper. The first category is the construction of a food system resilience indicator system. Early scholars believe that food system resilience is mainly the resistance ability after coping with unexpected shocks (such as natural disasters, financial, and political crises) [7]. Some scholars have also proposed that food system resilience indicates the risk adaptive capacity that can systematically anticipate and withstand pressure [8]. Hao Aimin et al. (2022), on the basis of the three dimensions of resistance, adaptive capacity, and transformative capacity, constructed an index of China's food system resilience [9]. Other scholars have also studied food production resilience [10], cropland system protection resilience [11], and food security resilience [12], providing important references for this paper. The second category focuses on the study of the digital economy and its impact on food system resilience. Some scholars argue that digital village construction [9], digital financial inclusion [13], and digital finance [14] are key factors in enhancing the resilience of the food system. Others believe that the digital economy is the core element in empowering the high-quality development of the food industry [15], improving the resilience of the food supply chain [16], and increasing food security capacity [17]. This suggests that it could be a significant opportunity for fostering the growth of the digital economy and, in turn, strengthening the resilience of the food system. The third category studies the role of industrial agglomeration paths. One view is that the digital economy can mobilize resources, break through spatial and temporal limitations, and promote the optimization and integration of the food industry chain, thereby weakening the role of industrial agglomeration [18]. Another view is that the food industry has a higher degree of external risk and relies more on the knowledge spillover effect of the digital economy and the function of the rapid exchange of information to improve risk management and decision-making efficiency. The digital economy exacerbates the “siphon effect” of the city on resources, leading to the formation of a network system to jointly protect against risks. To improve risk management and decision-making efficiency, operators in the food industry, along with technology and knowledge experts, will be gathered in a specific region to form a network system to jointly resist risks. This promotes industrial agglomeration in the digital economy [19].

The literature review shows that previous studies are a valuable reference for this paper. However, there are still some shortcomings that need to be addressed: The previous literature on food security has primarily focused on theoretical and logical analysis, with few empirical studies from the perspective of food system resilience. Additionally, while China has achieved remarkable results in the development of the digital economy, its role in supporting and empowering high-quality industry development is becoming more prominent, and there is a lack of research on the resilience of the digital economy

and the food system. Furthermore, few studies have explored the intrinsic connection between the three, including industrial agglomeration. Therefore, this paper presents a theoretical analytical framework for exploring the resilience of the current food system in the context of the digital economy, industrial agglomeration, and spatial and temporal characteristics. The framework examines the impact effects, internal mechanisms, and spatial spillover effects of the food system's resilience. By analyzing these elements and proposing countermeasures, this paper aims to provide a scientific basis for ensuring China's food security.

2. Theoretical Analysis and Research Hypothesis

2.1. Direct Effects of the Digital Economy on Food System Resilience

The theory of network economics posits that the data network is a productive tool and that market behavior, information acquisition, decision making, and trading are all closely linked to the data network. This is particularly true in the digital economy era, where the trend toward networked food production and operational activities is increasingly apparent. The integration of substitution effects enhances the resilience of the food system in the digital economy. T[illegible]ntegration of substitution effects enhances the resilience of the food system in the digital economy. This is achieved through networking, high-permeability effects, and sustainable practices, as well as information synergy, innovation, and creation effects. (1) It is important to consider the integration of substitution effects in this context. Urbanization has led to a loss of agricultural labor, resulting in the abandonment of arable land and a double predicament comprising a "shortage of resources" and "idle resources". The digital economy, with data as the core factor of production, differs from the traditional labor force and resource factors. Unlike traditional [illegible]r and other resource factors, the digital economy takes data as the core production factor. By replacing inefficient production factors, it increases factor inputs and promotes the effective use of resources. Additionally, the deep penetration and integration of the digital economy and the food industry can effectively empower the digital transformation and upgrading of the food industry. This improves the value of food industry factors and enhances the resistance of the food system [20]. (2) Synergy of information: The digital economy is characterized by networking through the Internet to realize the wide dissemination of information and data mining. It enables the effective management of "data islands" and overcomes data barriers, eliminating information asymmetry and externality problems. This allows food production and operation to utilize their own resources, achieving cross-regional and even cross-industry synergistic configurations and thus promoting decentralization in food production, circulation, sales, and services. This comprehensive extension of the food industry's chain optimizes the allocation of resources and enhances the adaptability of the food system [21]. (3) Innovation and creation effect: The development of the digital economy has opened a new industrial revolution round, reinventing not only the old organizational structure but also giving rise to new business models. This has promoted the transformation of the employment structure, improved the ecology of the employment market, and created a large number of new jobs for the food system, resulting in a significant creation effect [22]. The digital economy will also create diverse consumer demands and encourage constant reform and innovation in production and operations to adapt to market changes. This will inject new momentum for the transformation of the food system [23]. Therefore, we formulate the following hypothesis:

H1: Digital economic development can empower the enhancement of food system resilience.

2.2. Transmission Mechanisms of the Digital Economy to Enhance Food System Resilience

The theory of industrial agglomeration suggests that sharing resources, such as labor, capital, and information, can reduce production costs and promote the formation of

industrial agglomerations [24]. The digital economy facilitates universal resource sharing through modern information networks, which contributes to the development of industrial agglomerations. First of all, China's grain industry agglomeration is still facing problems such as insufficient technological innovation ability, weak market competitiveness, small-scale agglomeration, etc. In the context of the digital economy, high permeability facilitates the efficient flow and precise configuration of resource elements. This enables the effective convergence of upstream and downstream industrial chains, as well as production, supply, and marketing, leading to efficient operations. It also helps enhance the stability and competitiveness of the industrial supply chain, attracting more investment and production operators to enter the field. This provides a key driving force for the agglomeration of the grain industry [19]. Secondly, the digital transformation of infrastructure and the innovation and creation effects of digital technology can broaden the financing, information, and technology acquisition channels of producers and operators. This effectively bridges the industrial development bias caused by differences in resource endowment, environmental regulations, and the level of informatization among regions, thus promoting the development of industrial agglomeration [25].

However, theories such as MAR externality, Porter externality, and Jacob externality highlight the positive impact of industrial agglomeration on economic resilience. MAR externality emphasizes the benefits of industry-specialized agglomeration, while Porter externality and Jacob externality focus on industry-related diversification [26]. This is also applicable to the resilience of the food system. Firstly, industrial agglomeration is led by infrastructure construction, which cannot be separated from facilities such as information systems, logistics and transportation, payment and settlement systems, and a healthy and relaxed development environment [14]. Secondly, industrial agglomeration can promote the construction of an information-sharing platform. This platform can facilitate mutual exchanges and information circulation among production operators in the agglomeration area. For example, it can provide information on market trends and competitor dynamics. This can help production operators better cope with market risks and competitive pressures. Additionally, it can help them adapt to market changes more quickly, thus improving the risk-adaptive capacity of the entire industry. Thirdly, due to the agglomeration economy, operators in the same or similar food production areas become more concentrated, leading to increased competition among producers. This drives continuous self-innovation and the improvement of service quality to enhance competitiveness. Industrial agglomeration provides a specialized external environment for relevant production operators in the region to optimize the allocation of innovation resources; moreover, it stimulates the dynamics of change for the resilience of the food system through the competitive effect [27]. Therefore, the hypothesis proposed is as follows:

H2: Industrial agglomeration contributes to enhancing the resilience of the food system enabled by the digital economy.

2.3. Spatial Spillover Effects of the Digital Economy on Food System Resilience

The theory of new economic geography suggests that knowledge and technology spillovers are directional and scope-specific. As a result, economic activities will eventually be evenly distributed across space [28]. Therefore, the impact of the digital economy on the resilience of the food system is not limited to a single city or region but spreads spatially and affects neighboring regions, resulting in a spatial impact effect [29]. The digital economy relies on the high permeability and network connectivity of digital platforms, driven by information and communication technologies. It reduces information asymmetry, breaks through regional constraints, and boosts the free flow of resource elements between regions. This leads to the Pareto-optimal state of the cross-regional realization of factors such as land, capital, labor, and technology. Simultaneously, the digital economy facilitates the production and operation of online platforms for the food industry, enabling the development of a "dual-track drive" mechanism that links online and offline operations. This reduces the difficulty and cost of accessing information for the main production

and operation bodies, enhancing production efficiency and cultivating the resilience of the food system to cope with potential impacts [30]. Moreover, particularly in the field of food production, neighboring regions share similar climate, water, and resource endowments. This results in a stronger role for inter-market and product competition, as well as substitution effects. Technical exchanges and access to information are also more convenient. To achieve a higher competitive position, it is important to actively absorb good practices from neighboring regions and promote synergistic development between different regions. This will improve the quality of food and enhance the resilience of the food system. Therefore, the following hypothesis is formulated:

H3: Digital economy-enabled food system resilience has a positive spatial spillover effect.

3. Model Construction, Variable Selection, and Data Sources

3.1. Model Construction

3.1.1. Benchmark regression model

In order to verify the impact effect of digital economy-empowering food system resilience, the following benchmark regression model is constructed:

$$\mathrm{FSR_{it}} = \alpha_0 + \mathrm{cDig_{it}} + \beta_0 \sum \mathrm{Control_{it}} + \mu_i + \pi_t + \varepsilon_{it} \quad (1)$$

Equation (1) above shows the relationship between FSR_{it} and Dig_{it}, representing the resilience of the food system and the digital economy level of province i at time t, respectively. The set of control variables is denoted as $\sum Control_{it}$, while α_0 is a constant term. The correlation coefficients of the corresponding variables are represented by c and β_0, respectively. The province and year fixed effects are denoted by μ_i and π_t, respectively, and ε_{it} represents the random error term.

3.1.2. Mediation effects model

To further explore the role mechanism played by industrial agglomeration in the resilience impact of the digital economy-enabled food system, the following mediating effect model is constructed by drawing on the relevant research of Jiang Boat (2022) [31]:

$$\mathrm{Dig_{it}} = \alpha_1 + \mathrm{aAgg_{it}} + \beta_1 \sum \mathrm{Control_{it}} + \mu_i + \pi_t + \varepsilon_{it} \quad (2)$$

$$\mathrm{FSR_{it}} = \alpha_2 + \mathrm{c'Dig_{it}} + \mathrm{bAgg_{it}} + \beta_2 \sum \mathrm{Control_{it}} + \mu_i + \pi_t + \varepsilon_{it} \quad (3)$$

Equations (2) and (3) above use the term $\mathrm{Agg_{it}}$ to represent the degree of industrial agglomeration in province i at time t. The correlation coefficients for the corresponding variables are denoted by a, c′, and b. The remaining equations are consistent with equation (1).

3.1.3. Spatial measurement models

(1) Spatial autocorrelation: Spatial autocorrelation is commonly measured using the global Moran index I. This index ranges from -1 to 1, with values greater than 0 indicating a positive correlation, meaning high–high or low–low neighboring, and values close to 0 indicating no spatial autocorrelation. The formula for Moran index I is as follows:

$$I = \frac{\sum_{i=1}^{n}\sum_{j=1}^{n} W_{ij}(x_i - \overline{x})(x_j - \overline{x})}{s^2 \sum_{i=1}^{n}\sum_{j=1}^{n} W_{ij}} \quad (4)$$

In the above equation (4), $S^2 = \frac{\sum_{i=1}^{n}(x_i-\overline{x})^2}{n}$ is the sample variance, W_{ij} is the spatial weight matrix, and $\sum_{i=1}^{n}\sum_{j=1}^{n} W_{ij}$ is the sum of all spatial weights; in this paper, we utilize spatial proximity matrix; x_i and $x_j$denote the values of the indicators of provinces i and j, respectively.

(2) Spatial Durbin model: In order to explore the spatial spillover effects of the digital economy and the resilience of the food system, the spatial Durbin model is established as follows:

$$
\begin{aligned}
FSR_{it} &= \theta_0 + \lambda W FSR_{it} + \delta_1 DIg_{it} + \theta_1 WDIg_{it} \\
&+ \delta_2 \sum Control_{it} + \theta_2 W \sum Control_{it} + \mu_i + \pi_t + \varepsilon_{it}
\end{aligned} \quad (5)
$$

In Eq. (5), λ is the spatial correlation coefficient of the resilience of the food system; $W(\bullet)$ is the spatial lag variable; δ and θ are the coefficients of each variable; the rest is consistent with Eq. (1).

3.2. Variable Selection

3.2.1. Explanatory variable: food system resilience (FSR)

Based on the theoretical analysis and relevant literature [9,13,14,32], we selected 17 indicators to construct the resilience index system for the food system. The system is constructed from three dimensions: resistance, adaptability, and changeability (refer to Table 1). To avoid measurement bias caused by inconsistent indicators, this paper uses the entropy method to standardize indicators and measure food system resilience objectively by assigning weights to each indicator. This is then integrated into a composite index, drawing on the research of Zhu Xi'an et al. (2015) [33].

Table 1. Index system of food system resilience.

Primary index	Secondary index	Index calculation method and unit	Property	Weight
Capacity for resist	Cultivated land per capita	Cultivated land area/number of persons employed in the primary sector (ha/person)	+	0.102
	Labor force level	Number of people employed in primary industry/total employment (%)	+	0.037
	Level of fixed asset investment	Investment in agricultural fixed assets / Gross regional product (%)	+	0.054
	Grain production per capita	Total grain production/total number of people in the province (kg/person)	+	0.082
	Food production price index	Price of grain production in the current/base period (%)	-	0.016
	Grain production level	Grain production/grain sown area (t/ha)	+	0.022
Capacity for capacity	Intensity of pesticide use	Pesticide use / area sown with crops (t/ha)	-	0.001
	Degree of disaster	Crop damage area / crop sown area (%)	-	0.005
	Fertilizer use intensity	Amount of agricultural fertilizer applied (pure) / area sown with crops (t/ha)	-	0.016
	Intensity of agricultural film use	Agricultural plastic film use/crop sown area (t/ha)	-	0.008
	Replanting index	Grain sown area/cultivated area (%)	+	0.028
	Agricultural value add index	Agricultural value add index (%)	+	0.005
	Per capita rural electricity consumption,	Rural electricity consumption/rural population (10 thousand* kW·h/person)	+	0.297
Capacity for change	Degree of mechanization	Total power of agricultural machinery/area sown with crops (10*kW)/ha	+	0.047
	Level of agricultural development	Gross agricultural output value / gross regional product (%)	+	0.052
	Level of Expenditure on Agricultural Research	Local fiscal expenditure on science and technology* gross agricultural output value/gross regional product (CNY billion)	+	0.088
	Level of agricultural technicians	Persons obtaining national vocational qualification certificates in the grain industry/number of employees in primary industry (%)	+	0.138

3.2.2. Core explanatory variable: level of digital economy (Dig)

The literature on constructing a digital economy index system primarily focuses on digital economy carriers, industrial digitization, and digital industrialization [34–36]. This paper selects 19 indicators for measurement (Table 2). The steps for measuring the

comprehensive index of the digital economy level using the entropy value method are consistent with those for measuring the food system resilience index.

Table 2. Index system of the digital economy.

Primary index	Secondary index	Index calculation method and unit	Property	Weight
Carrier of digital economy	Cell phone penetration	Number of cell phone subscribers per 100 people (households/100 people)	+	0.011
	Internet penetration	Number of Internet users as a proportion of resident population (%)	+	0.011
	Breadth of information transmission	Density of fiber-optic cable lines (km/km2)	+	0.060
	Signal coverage breadth	Density of cell phone base stations (pcs/km2)	+	0.077
	Internet broadband infrastructure	Density of Internet broadband access ports (pcs/km2)	+	0.081
	Strength of investment in digital services	Per capita fixed asset investment in information transmission, computer services and software industry (CNY/person)	+	0.027
Digital industrialization	Development level of post and telecommunications industry	Total telecommunication business per capita (CNY/person)	+	0.050
		Total postal services per capita (CNY/person)	+	0.074
		Volume of express delivery (ten thousand pieces)	+	0.114
	Development level of electronic information manufacturing industry	Revenue of electronic information manufacturing industry (CNY)	+	0.094
		Number of enterprises in electronic information manufacturing industry (nos.)	+	0.092
	Software and information technology service industry	Software business revenue (CNY)	+	0.090
		Number of employees in information transmission, software and information technology services (ten thousand)	+	0.054
Digitalization of industries	Development level of enterprise digitization	Number of websites owned by enterprises (number)	+	0.044
		Share of enterprises with e-commerce transaction activities (%)	+	0.016
		E-commerce transaction volume (CNY billion)	+	0.069
	Development level of digital inclusive finance	Digital finance coverage breadth index	+	0.013
		Depth of digital finance usage index	+	0.012
		Digital finance digitization degree	+	0.010

3.2.3. Mediating variable: food industry agglomeration (Agg)

Drawing on Han Haibin et al. (2023) and Yang Xiuyu et al. (2023) [37,38], the degree of food industry agglomeration is measured via locational entropy, which overcomes the influence of regional scale differences and other effects, and it measures the degree of agricultural agglomeration more accurately with the following formula:

$$Agg_{it} = (A_{it} \,/\, G_{it})/(A_t \,/\, G_t) \quad (4)$$

Equation (3) defines Agg_{it} as the degree of food industry agglomeration in province i at time t, which is proportional to its value. A_{it} and G_{it} represent the total output value of agriculture, forestry, livestock, and fishery, and the gross regional product of region i in year t, respectively. A_t and G_t represent the total output value of agriculture, forestry, livestock, and fishery and the gross domestic product of the entire country in year t, respectively.

3.2.4. Control variable

To ensure the resilience of the food system, the following control variables are primarily considered to control the impact of other variables:

1) The level of informatization is expressed by the ratio of the total amount of post and telecommunication services to GDP.
2) Employment density is represented by the ratio of the number of employees in the primary industry to the area of administrative divisions.

3) Foreign direct investment is expressed as the ratio of foreign direct investment to GDP after multiplying the current USD/CNY exchange rate.
4) The level of development in the technology market can be measured using the ratio of technology market turnover to GDP.
5) The degree of industrialization can be measured using the ratio of the industrial added value to GDP.
6) The degree of openness to the outside world can be measured using the ratio of the value of imports and exports of goods, multiplied by the USD/CNY exchange rate of the current year, to GDP.

3.3. Data sources

Considering the availability of data, this paper uses 30 provinces in China (except Tibet, Hong Kong, Macao, and Taiwan) from 2011 to 2021 as the research object, and the data are mainly obtained from the China Rural Statistical Yearbook, China Statistical Yearbook, EPS database, and statistical yearbooks and statistical bulletins of the corresponding years in each province and city. Linear interpolation is used to add some missing values. The descriptive statistics of each variable are shown in Table 3.

Table 3. Descriptive statistics of variables.

Variable	Symbol	Mean	SD	Min	Max
Resilience of food system	FSR	0.195	0.058	0.114	0.463
Digital economy	Dig	0.119	0.108	0.010	0.647
Industrial agglomeration	Agg	1.185	0.623	0.049	3.467
Informatization level	Inf	0.060	0.055	0.014	0.290
Employment density	Emp	0.026	0.039	0.000	0.217
Foreign direct investment	For	0.019	0.015	0.000	0.080
Technology market development level	Tec	0.016	0.029	0.000	0.175
Degree of industrialization	Ind	0.321	0.082	0.101	0.556
Degree of opening up	Open	0.265	0.291	0.008	1.548

4. Results Analysis and Discussion

4.1. Spatial and temporal characterization of food system resilience

This paper examines the spatial and temporal characteristics of China's food system resilience by estimating the kernel density map through plotting. Figure 1 shows that the center of kernel density moves to the right each year, indicating an improvement in the resilience of the food system and effective food security guarantee. The distribution curves of each year indicate that the resilience of the food system in most regions of China is still at a middle to lower level. The curves also show a unipolar phenomenon during the examination period. These findings suggest that the food system in China needs improvement. Additionally, the curves for each year exhibit a clear "right trailing" pattern, suggesting that the resilience of the food system during the study period is unipolar. Overall, China's food system has significantly improved in recent years. However, there are still substantial variations in the resilience levels across different regions, with a larger proportion of regions having lower resilience levels. Therefore, there is a need to strengthen the resilience level of the food system.

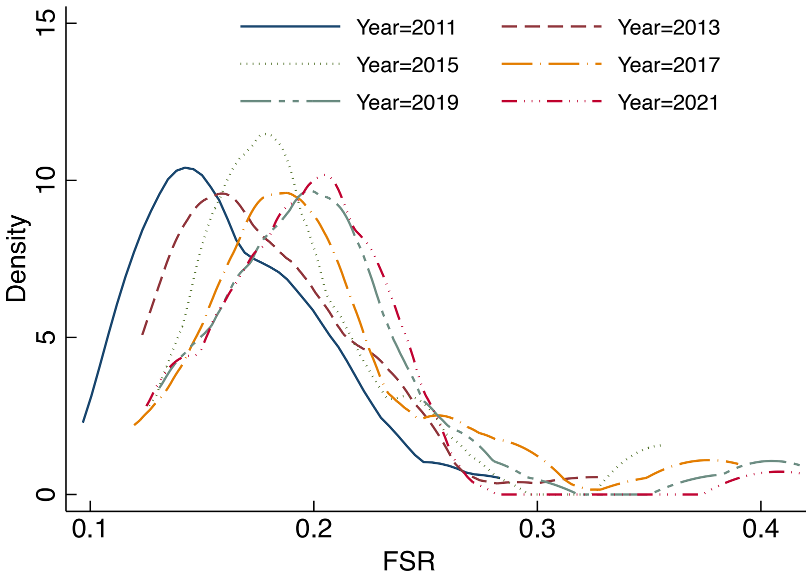

Figure 1. Spatial and temporal characteristics of the resilience of China's food system.

4.2. Analysis of baseline regression results

Prior to conducting empirical analyses, the variables were assessed for multicollinearity. The results indicate that Max-VIF is 3.39, which is significantly lower than the threshold of 10, thus indicating the absence of multicollinearity. The impact of the digital economy on the resilience of the food system was analyzed using the baseline regression model. The results are presented in Table 4, with columns (1) to (4) showing the results of no fixing, province fixing, year fixing, and province and year fixing after the introduction of the control variables, respectively. The results indicate that the regression coefficients of the digital economy are all positive and pass the significance test at the 10% level when the province and year effects are fixed. This suggests that the digital economy significantly enhances the level of food system resilience. Possible reasons for this include the use of digital technologies such as big data and intelligent control to achieve precision planting, intelligent fertilizer application, and automated irrigation. This enhances food resistance through precision and intelligent agriculture. It promotes the rational use of natural resources, such as land and water, and supports sustainable food production. Additionally, it enhances the adaptive capacity of the food system. The promotion of digital transformation in the food industry chain encourages food operating entities to accelerate technological progress, improve technological efficiency, and ultimately enhance the resilience of the food system [14].

When controlling for province and year effects, the level of informatization showed a significant positive impact on the resilience of the food system. This may be due to its ability to facilitate information sharing, reduce information asymmetry, improve coordination within the food system, enhance its ability to cope with external shocks, and ultimately increase its resilience [39]. Additionally, the level of employment density also had a significant positive impact on the resilience of the food system at the 1% level. The resilience of the food system was found to have a statistically significant positive effect at the 1% level in areas with high employment density. This may be due to the fact that such areas are conducive to the better management of food producers and operators, improve the quality and skill levels of food producers and operators, and have a better ability to cope with external shocks and risks in the food industry [40]. Foreign direct investment was found to have a statistically significant negative effect on the resilience of the food system at the 1% level. Foreign direct investment may lead to increased market competition for domestic food production operators, resulting in the use of agricultural chemicals and other means of production to enhance market competitiveness. This may not be

conducive to the stabilization of food system resilience [41]. Statistical analyses show that technological market development has a positive and significant impact on food system resilience at the 5% level [42]. This may be due to the fact that technological market development enables food production operators to quickly adopt new technologies, modes, and business models, which in turn improves production efficiency, product quality, and added value in the food industry. As a result, the resilience of the food system is enhanced.

Table 4. Descriptive statistics of variables.

Variable	(1) FSR	(2) FSR	(3) FSR	(4) FSR
Dig	0.164***（0.028）	0.102**(0.047)	0.121***(0.038)	0.111*(0.063)
Inf	0.003（0.056）	0.077**(0.032)	-0.147(0.115)	0.255***(0.084)
Emp	1.012***（0.108）	2.326***(0.641)	0.987***(0.119)	2.384***(0.637)
For	-0.336*(0.184)	-0.431***(0.131)	-0.362*(0.192)	-0.462***(0.140)
Tec	-0.240***（0.091）	0.444**(0.193)	-0.275***(0.092)	0.462**(0.192)
Ind	-0.081*(0.045)	-0.092**(0.044)	-0.070(0.044)	-0.068(0.050(
Open	-0.112***(0.012)	-0.022(0.024)	-0.096***(0.017)	-0.016(0.025)
Year	No	No	Yes	Yes
province	No	Yes	No	Yes
Sample	330	330	330	330
R^2	0.293	0.863	0.302	0.872

Note: Robust standard error in brackets: * * *, * *, and * indicate significant at 1%, 5%, and 10% levels, respectively. The same below.

4.3. Robustness Test

In order to verify the reliability of the findings, a robustness test was conducted (Table 5).

4.3.1. Adjustment of the sample period

The sample period is adjusted to 2018-2021 and 2011-2019, respectively. The reasons are as follows: In 2017, the digital economy was written in the government work report for the first time, and the development of the digital economy opened a new chapter; this policy factor may affect research conclusions, and only the sample after the policy's release is retained for analyses. The regression results are shown in column (1). In 2020, there was a large impact of the New Crown Epidemic on both the development of the digital economy and food production, and in order to exclude the impact of this major environmental change, only the samples before 2020 were analyzed. The regression results are shown in column (2). Columns (1) and (2) show that after adjusting for the sample period separately, the digital economy still passes a positive significance test at the 10% level for food system resilience.

Table 5. Robustness test.

Variable	(1) FSR	(2) FSR	(3) FSR
Dig	0.214*（0.120）	0.127*(0.069)	— —
Dig#	— —	— —	0.181**（0.084）
Control variable	Yes	Yes	Yes
Year	Yes	Yes	Yes
Province	Yes	Yes	Yes
Sample	120	270	330
R^2	0.959	0.886	0.865

4.3.2. Replacement of core explanatory variables

The entropy value method was used in the previous section to measure the composite index, and the CRITIC and entropy value methods are both objective weighting methods. The CRITIC method avoids the problem of weighting when dealing with the comprehensive evaluation of multiple indicators, but it also takes into account the effect of correlations on the weight of indicators and eliminates the effect of correlation indicators on the weight during weighting [43]. Using this method to re-measure the level of digital economy development and define the new variable as "Dig#", the regression results are shown in column (3) of Table 5. The results show that the estimation of the digital economy with respect to the resilience of food systems is still significant after using the CRITIC method to measure the digital economy. This indicates that the development of the digital economy increases the level of resilience of the food system.

4.4. Endogeneity Test

Despite controlling for as many key variables as possible in this paper, endogeneity may still be an issue. Due to the large number of factors affecting food system resilience, it is not possible to completely prevent, for example, the occurrence of omitted variables and bidirectional causality, which could lead to biased estimation results. Since there is a possibility that the level of food system resilience in turn affects the development of the digital economy, regions with faster digital economy development have a stronger comprehensive food production capacity and risk resistance capacity per se and higher food system resilience, and it is not possible to truly identify whether the increase in food system resilience is caused by the development of the digital economy [15]. Therefore, in this study, based on the experience of Huang Qunhui et al. (2019) [44] and the availability of data, the number of landline phones at the end of 1990 (in 10,000 households) in each region is selected as the instrumental variable, and a regression test is conducted using the two-stage least squares method.

The main considerations for the choice of this instrumental variable are that, on the one hand, the core of the development of the digital economy cannot be separated from the development of the Internet, and Internet technology is a continuation of the popularization of fixed-line telephony; i.e., The popularization of fixed-line telephony is one of the important infrastructures for the development of the digital economy, which satisfies the relevance requirement [45]. On the other hand, with the rapid application of modern communication technology, the role that traditional communication tools, such as fixed-line telephony, play in the development of today's food system resilience is minimal, and this satisfies the exclusivity requirement [9]. It is worth noting that the number of landline telephones at the end of 1990 comprises cross-sectional data, while the sample in this paper comprises balanced panel data. Therefore, based on Nunn and Qian (2014) [46], the cross-multiplier term between the number of Internet access instances in the previous year and the number of landline telephones at the end of 1990 is constructed as an instrumental variable (IV) for the digital economy index of the current period. The estimation results are presented in Table 6. The results in column (1) indicate that the instrumental variable has a significant positive effect on the digital economy, and the results in column (2) show that after applying the two-stage least squares method to mitigate the endogeneity problem, the digital economy still passes the positive significance test on the resilience of the food system and is significant at the 5% level, which is basically the same as the results of the benchmark regression. In addition, the results of the weak instrumental variable test show that the F-statistic is 241.435, which is significantly greater than the critical value of 10; therefore, there is no weak instrumental variable problem. Accordingly, this indicates that the development of the digital economy determines the improvement of the resilience of the food system, which verifies hypothesis H1.

Table 6. Results of the endogeneity test.

Variable	Instrumental variable method	
	（1）Dig	（2）FSR
IV	6.38e-08***（4.12e-09）	— —
Dig	— —	0.103**（0.051）
Control variable	Yes	Yes
Year	Yes	Yes
Province	Yes	Yes
F-value		241.435
Sample	330	330
R^2	0.955	0.895

4.5. Endogeneity Test

To further test the mediating effect of food industry agglomeration on the impact of the digital economy on food system resilience, the mediating effect model constructed in the previous section is used for validation (Table 7). As observed in Table 7, in column (1), the digital economy on food industry agglomeration passed the positive significance test at the 1% level, indicating that the development of the digital economy promotes the food industry's agglomeration level. In column (2), after the introduction of the digital economy variable, both the digital economy and food industry agglomeration on food system resilience passed the positive significance test at the 1% level, indicating that food industry agglomeration enhances the level of food system resilience. Accordingly, this indicates that the digital economy can enhance the resilience level of the food system by promoting the degree of food industry agglomeration. Food industry agglomeration plays a mediating effect on the impact of the digital economy on the resilience of the food system.

Table 7. Results of the endogeneity test.

Variable	（1）Agg	（2）FSR
Dig	1.912***(0.474)	0.235***(0.066)
Agg		0.065***(0.008)
Control variable	Yes	Yes
Year	Yes	Yes
province	Yes	Yes
Sample	330	330
R2	0.955	0.895

In order to test the reliability of the above mediated effect results, the self-sampling test method (bootstrap) was used to conduct a robustness test of the above results, and the number of bootstrap replicate samples was set at 500. The results are shown in Table 8. The results show that the coefficients of the indirect and direct effects are positively significant at the 1% level, and the 95% confidence interval does not include 0, which is basically consistent with the above conclusion. The mediating effect of the agglomeration of the food industry is again verified. Hypothesis H2 is therefore confirmed.

Table 8. Tests the mediation effect based on the bootstrap method.

Influence type	Coefficient	SD	P-value	[illegible]% confidence interval
Indirect effect	0.068	0.019	0.000	0.036
Direct effect	0.232	0.036	0.003	0.174

4.6. Analysis of Spatial Spillover Effects

4.6.1. Spatial autocorrelation test

Before carrying out the spatial econometric model, this paper adopts the global Moran's index (Moran's I) to calculate the spatial correlation between digital economy and food system resilience in each region under the spatial proximity matrix, and the results are shown in Table 9. The results show that Moran's index of digital economy and food system resilience for each year passed the positive significance test, indicating that there is an obvious positive spatial correlation between the digital economy and food system resilience in our region during the study period.

[illegible]le 9. Spatial autocorrelation test.

Year	Digital economy		Food system resilience	
	**Moran's I**	**Z-value**	**Moran's I**	**Z-value**
2011	0.261**	2.488	0.463***	4.081
2012	0.300***	2.813	0.441***	3.951
2013	0.266**	2.536	0.408***	3.781
2014	0.262**	2.503	0.433***	4.072
2015	0.281***	2.660	0.299***	2.890
2016	0.272**	2.592	0.268**	2.583
2017	0.251**	2.421	0.240**	2.345
2018	0.227**	2.240	0.153*	1.695
2019	0.216**	2.160	0.269***	2.699
2020	0.217**	2.162	0.233**	2.415
2021	0.223**	2.221	0.163*	1.835

We then plotted a Moran scatterplot of the digital economy and food system resilience in 2021 using Stata16 software (Figure 2). The results show that most areas are distributed in the high–high and low–low quadrants, indicating the spatial clustering of the digital economy and food system resilience and initially validating the spillover effect between the two.

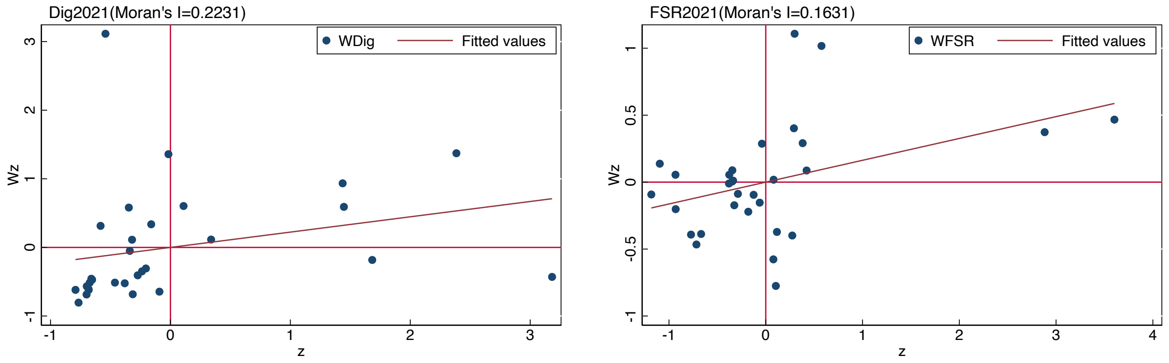

Figure 2. Moran's I scatter plot of the digital economy and food system resilience in 2021.

4.6.2. Spatial spillover effects

Before conducting empirical analyses, it is necessary to determine which spatial econometric model to select, and the results of the [illegible] LR, and Hausman tests show that the spatial Durbin model is superior to the spatial lag model and the spatial error model. In order to compare the test results, the regression results of the spatial Durbin model (SDM) and the spatial autoregressive model (SAR) were reported simultaneously, following Yi Enwen (2023) [45] (Table 10). The results show that the effect of the digital economy on local food system resilience (direct effect), the effect of the local digital economy on food system resilience in neighboring regions (indirect effect), and the total effect (the sum of direct and indirect effects) all passed the positive significance test. On the one hand, the development of the digital economy can promote the digital transformation of the local

food industry chain. It can improve the efficiency and resilience of all aspects of food production, circulation, and consumption; for example, the digital economy can help food production operators more accurately manage arable land and optimize the structure of grain cultivation, etc. Moreover, the optimization of logistics can be promoted. On the other hand, the development of the local digital economy can attract the inflow of talent and resources from neighboring regions and promote the exchange and sharing of digital technology, the construction of digital infrastructure, and the development of digital industrialization in neighboring regions, which in turn enhance the resilience of the food system in neighboring regions. Therefore, hypothesis H3 is supported.

Table 10. Test results of the spatial spillover effect.

Variable	SDM	SAR
Digital economy	0.371***(0.037)	0.381***(0.035)
W×Dig	0.060*(0.044)	
rho	0.177**(0.083)	0.211***(0.076)
sigma2_e	0.000***(0.000)	0.000***(0.000)
Direct effect	0.378***(0.039)	0.387***(0.037)
Indirect effect	0.151**(0.071)	0.103**(0.046)
Total effect	0.530***(0.058)	0.491***(0.065)
Control variable	Yes	Yes
Year	Yes	Yes
Province	Yes	Yes
Sample	330	330
Within R^2	0.366	0.458

5. Conclusions and Policy Recommendations

5.1. Conclusions

This study takes the panel data of 30 provinces in China from 2011 to 2021 as a sample; constructs an index system of China’s digital economy and food system resilience; draws a kernel density map to analyze the spatial and temporal differences in the characteristics of China’s food system resilience; uses the double fixed-effects model to test the impact effect of the digital economy on food system resilience; and applies the mediating effects model to clarify the transmission mechanisms of the digital economy on food systems through the industry. Using the mediating effects model, we clarified the transmission mechanism of agglomeration in the digital economy with respect to food system resilience and used the spatial measurement model to explore the spatial spillover effect of the digital economy on food system resilience. The results of the study show that, firstly, China’s current food system resilience level in the time series shows a stable growth trend, but with respect to the spatial difference, it is obvious that the level of food system resilience still needs to be strengthened as the region's low levels accounted for a large proportion of the narrowing of regional differences. Second, the digital economy plays an important role in promoting the resilience of the food system, and this conclusion is still valid after considering the endogeneity and robustness test. Therefore, in the process of promoting the resilience of the food system driven by the digital economy, the “digital dividend” effect plays an important role, which provides empirical evidence for ensuring China’s food security and comprehensively promoting the rural revitalization strategy. Third, promoting industrial agglomeration is an inherent mechanism of the digital economy to enhance the resilience of the food system. Driven by the digital economy, resource elements in the agglomeration area will be revitalized, and production capacities can be released to promote the digital transformation of the food industry, which in turn further enhances the resilience of the food system. Fourth, there is a positive spatial spillover effect: i.e., the development of the digital economy at the provincial and national food

system resilience levels has an important role in promoting the resilience of the food system in neighboring provinces and cities; simultaneously, there is a significant positive impact on the resilience of the food system.

5.2. Policy Recommendations

First, we recommend the following: improve infrastructure construction and promote the development of digital villages. To make up for the lack of rural digital infrastructure, focus should be placed on supporting the digital transformation of rural mobile communication base stations and terminals and accelerating the construction of convenient, high-speed, and smooth mobile communication networks, especially with respect to providing 5G networks in remote rural areas. Moreover, the application of 5G in rural areas should be further explored, which includes improving the perception, collection, screening, and transmission of data and information; creating a digital information "highway" in the grain industry; and consolidating the foundation of digital economy development on all fronts. In addition, it is necessary to enhance the digitalization awareness of grain farmers and their ability to apply information technology, and the following principle should be taken as the starting point to promote the development of intelligent agriculture for grain farmers: "promoting the development of intelligent agriculture, promoting the integration and application of information technology and agricultural machinery and agronomy". In addition, the following ideologies are recommended: the ideological awareness of intelligent agricultural development and practical food and agriculture skills should be enhanced; digital agriculture and skill training for local food and agriculture should be carried out; the high-quality development of food should be ideologically established; digital agricultural literacy should be effectively cultivated; the cultivation of new vocational digital agriculture should be accelerated.

Second, the construction of industrial agglomeration should be strengthened, and the quality and efficiency of food should be promoted. Industrial agglomeration plays an important role in promoting the resilience of the digital economy-based food system. On the one hand, it is necessary to improve market-oriented development to attract the development of upstream and downstream production and operation clusters. Simultaneously, it is also necessary to strengthen the drive for scientific and technological innovation, such as the implementation of large-scale innovation projects with respect to regional characteristics and field advantages. Moreover, investments in research and development and talent team-building efforts should be increased in order to encourage agriculture-related college students, postgraduate students, and agricultural technicians to carry out grassroots fieldwork. On the other hand, we should create a good development environment; focus on creating a market-oriented international business environment that follows the rule of law; improve financial literacy; avoid blindly attracting foreign investment; and focus on changes from "quantity" to "quality" at the agricultural enterprise scale. The quality and efficiency of industrial development will be enhanced through multiple channels and initiatives, and the resilience of the food system will be gradually improved.

Third, regionally linked development should be promoted to lay a solid foundation for food security. The results show that the resilience of the food system in each region has a wide range of linkages. Thus, each region should adapt to local conditions; formulate a food development strategy that is highly compatible with its own actual situation; and focus on promoting measures to improve the quality of specialty foods, creating local specialty food brands and realizing the development of a new business model for the specialty food industry. On the other hand, it is necessary to strengthen exchanges and cooperation in the food industry among cities, promote the construction of a unified national market, eliminate the "local" thinking of some local governments, and promote the development of the food industry in a manner that effectively connects the "hardware" and "software" across regions. The effective convergence of "hardware" and "software" is a method for promoting platform building, industrial integration, public services, talent exchange, etc., in administrative regions.

5.3. Main Limitations

This paper is dedicated to exploring the relationship between the digital economy and food system resilience, and empirical methods have been used to carry out useful explorations. However, due to datum limitations and the author's lack of experience, there is still room for improvement and exploration. Firstly, although the scientificity and completeness of the indicators for measuring the digital economy and food system resilience have been taken into account as much as possible, the complexity of food production activities and the availability of data inevitably lead to the omission of variables, which in turn imparts a certain degree of bias in the analysis's results. Secondly, although the scale of the study has a certain degree of depth and the data of the study are relatively fine, gaps still exist compared to studies carried out at the county or township scale; further in-depth research will be carried out with respect to these factors.

Author Contributions: Conceptualization, Z.Z. and Z.W.; data curation, Z.W.; formal analysis, Z.Z.; funding acquisition, Z.Z. and B.L.; methodology Z.Z., Z.W. and B.L; software, Z.W. and Z.Z.; writing—original draft, Z.Z. and B.L.; writing—review and editing, Z.Z. and Z.W.; Z.Z. and Z.W. contributed equally to this work and should be regarded as co-first authors. All authors have read and agreed to the published version of the manuscript.

Funding: This research was funded by the National Natural Science Foundation of China (grant no. 71563016), Key Research Base Project of Humanities and Social Sciences in colleges and universities of Jiangxi Province (JD21080), the Jiangxi Postgraduate Innovation Special Fund Project (YC2022-s415), and the Jiangxi Agricultural University School of Economics and Management 2022 Postgraduate Innovation Special Fund Project (grant no. JG2022008).

References

1. Wang, K. Achievements and Experiences of China's Food Security in the New Era. *Reform* **2023**, *9,* 99–109.
2. Luo, B. Seed industry revitalization and food security: A review. *J. Sou. China Agric. Uni.* **2023**, *44*, 827–836.
3. Kang, L. Food security in Central Asia under the new situation：Current situation，causes and path selection. *J. China Agric. Uni.* **2024**, *29*, 258–270.
4. Zhang, Y.; Wang, M.; Liao, Y. Rice Industrialization from the Perspective of Ensuring National Food Security: Based on Monitoring Data of 2,225 Rice Enterprises. *Reform* **2022**, *11,* 119–129.
5. Gao, W. Mechanism of Industrial Agglomeration Driving High-quality Development of Grain. *J. Sou. China Agric. Uni. (Soc. Sci. Edi.)* **2021**, *20*, 80–94.
6. Ren, S.; Li, L.; Han, Y.; Hao, Y.; Wu, H. The Emerging Driving Force of Inclusive Green Growth: Does Digital Economy Agglomeration Work? *Bus. Strateg. Environ.* **2022**, *31*, 1656–1678, doi:10.1002/bse.2975.
7. Tendall, D.M.; Joerin, J.; Kopainsky, B.; Edwards, P.; Shreck, A.; Le, Q.B.; Kruetli, P.; Grant, M.; Six, J. Food System Resilience: Defining the Concept. *Glob. Food Secur.-Agric.Policy* **2015**, *6*, 17–23, doi:10.1016/j.gfs.2015.08.001.
8. Coopmans, I.; Bijttebier, J.; Marchand, F.; Mathijs, E.; Messely, L.; Rogge, E.; Sanders, A.; Wauters, E. COVID-19 Impacts on Flemish Food Supply Chains and Lessons for Agri-Food System Resilience. *Agric. Syst.* **2021**, *190*, 103136, doi:10.1016/j.agsy.2021.103136.
9. Hao, A.; Tan, J. Impact of Digital Rural Construction On Food System Resilience. *J. Sou. China Agric. Uni. (Soc. Sci. Edi.)* **2022**, *21*, 10–24.
10. Jiang, H.; Chen, Y.; Liu, Z. Spatiotemporal Pattern and Influencing Factors of Grain Production Resilience in China. *Econo. Geogr.* **2023**, *43*, 126–134, doi:10.15957/j.cnki.jjdl.2023.06.013.
11. Hu, L.; Guo, J.; Ou, M.; Qian, H. The "Rigidity-Elasticity-Resilience" of Systematic Farmland Protection: Theoretical Framework and Implementation Path. *Planners* **2023**, *39*, 7–13.

12. Qing, P; Deng, X.; Min, S. Food Security Resilience and Risk Management Strategy in China in the Context of "Dual Circulation". *Strat. Stu. CAE* **2023**, *25*, 26–38, doi:10.15302/J-SSCAE-2023.04.002
13. Li, M.; Chen, D; Yu, G. Digital inclusive finance enhances food system resilience: from the perspective of spatial spillover effect. *Chin. J. Agric. Reso. Reg. Plan.* **2023,** *6,* 1–13.
14. Wang, Y.; Ye, J.; Cao, J. Study on the Mechanism and Effect of Digital Finance Improving the Resilience of Grain System. *Econ. Sur.* **2023**, *40*, 48–60, doi:10.15931/j.cnki.1006-1096.2023.05.003.
15. Zuo, X.; Ye, L.; Ma, J. Empirical Test on the Impact of Digital Economy on High-quality Development of Grain Industry. Stati. & Dec. **2023**, *39*, 36–41, doi:10.13546/j.cnki.tjyjc.2023.22.006.
16. Ma, J.; Li, G.; Han, D. Digital economy empowers food supply chain resilience: path of action and policy orientation. Soc. Sci. Xinjiang **2023**, *1,* 46–54, doi:10.20003/j.cnki.xjshkx.2023.01.006.
17. Qi, Y.; Wang, Y.; He, Q. Spatio-temporal Evolution and Impact Analysis of Digital Economy Development and Food Security Capacity in the Yangtze River Economic Belt. *Rural Economy* **2023**, 48–58.
18. He, P.; Tan, C. E-Commerce and Rural Economic Resilience: Empirical Evidence Based on the "Comprehensive Demonstration E-commerce into Rural Areas" Policy J. *Zhongnan Uni. Econ. Law* **2023**, 97–108, doi:10.19639/j.cnki.issn1003-5230.2023.0009.
19. Kou, D.; Zhang, C.; Zhong, W. The impact of digital economy on industrial agglomeration and its empirical analysis. *South. Aca. Rese.* **2023**, 147–157, doi:10.13658/j.cnki.sar.2023.06.009.
20. Ouyang, R. The Institutional Logic of Data Element Circulation. *Frontiers* **2023**, 13–27, doi:10.16619/j.cnki.rmltxsqy.2023.06.002.
21. Chen, M.; Zhang, X. The Scale Measurement and Productivity Analysis of China's Digital Economy. *J. Quan. Tech. Econ.* **2022**, *39*, 3–27, doi:10.13653/j.cnki.jqte.2022.01.004.
22. Su, Y.; Lu, J. Development of the Digital Economy,Job Tasks and Job Changes. *Popula. J.* 2023, 45, 69–82, doi:10.16405/j.cnki.1004-129X.2023.05.006.
23. Guo, K.; Liu, C. Platform Anti-Monopoly, Digital Economy Innovation and Industrial Structure Upgrading. *China Indus. Eco.* 2023, 61–79, doi:10.19581/j.cnki.ciejournal.2023.10.003.
24. Picard, P.M.; Zeng, D.Z. Agricultural Sector and Industrial Agglomeration. *J. Dev. Econ.* **2005**, *77*, 75–106, doi:10.1016/j.jdeveco.2004.03.002.
25. Mao, N.; Sun, W.; Yang, Y. Transportation Infrastructure Construction and Enterprise Digital Transformation—An Empirical Study on China's High-speed Railway. *J. Quan. Tech. Econ.* **2022**, *39*, 47–67, doi:10.13653/j.cnki.jqte.20220920.003.
26. Deng, Y.; Sun, H. The Effect of Industrial Agglomeration on Economic Resilience and Its Mechanism. *Soft Sci.* **2022**, *36*, 48-54+61, doi:10.13956/j.ss.1001-8409.2022.03.07.
27. He, Z.; Li, Y.; Wu, Y. Industrial Collaborative Agglomeration, Technological Innovation and Manufacturing Industrial Chain Resilience. *Stu. Sci. Sci.* **2023**, 1–16, doi:10.16192/j.cnki.1003-2053.20230515.001.
28. Chen, J.; Chen, G.; Huang, J. A Study of Productive Service Industry Agglomeration and Its Influencing Factors Under the Perspective of New Economic Geography:Empirical Evidence from 222 Cities in China. *J. Manag. Wor.* **2009**, 83–95, doi:10.19744/j.cnki.11-1235/f.2009.04.010.
29. Zhao, T.; Zhang, Z.; Liang, S. Digital Economy, Entrepreneurship, and High-Quality Economic Development: Empirical Evidence from Urban China. *J. Manag. Wor.* **2020**, *36,* 65–76, doi:10.19744/j.cnki.11-1235/f.2020.0154.
30. Zhao, W.; Xu, X. Effect and Mechanism of Digital Economy on the Resilience of Agricultural Economy. *J. Sou. China Agric. Uni. (Soc. Sci. Edi.)* **2023**, *22,* 87–96.
31. Jiang, T. Mediating Effects and Moderating Effects in Causal Inference. *China Indu. Econ.* **2022**, 100–120, doi:10.19581/j.cnki.ciejournal.2022.05.005.

32. Zurek, M.; Ingram, J.; Bellamy, A.S.; Goold, C.; Lyon, C.; Alexander, P.; Barnes, A.; Bebber, D.P.; Breeze, T.D.; Bruce, A.; et al. Food System Resilience: Concepts, Issues, and Challenges. *Annu. Rev. Environ. Resour.* **2022**, *47*, 511–534, doi:10.1146/annurev-environ-112320-050744.
33. Zhu, X.; Wei, G. Discussion on the Good Standard of Dimensionless Method in Entropy method. *Stat. Dec.* **2015**, 12–15, doi:10.13546/j.cnki.tjyjc.2015.02.003.
34. Wang, J.; Zhu, J.; Luo, Q. Development Level and Evolution Measurement of China's Digital Economy. *Resea. Quant. Tech. Econ.* **2021**, *38,* 26–42, doi:10.13653/j.cnki.jqte.2021.07.002.
35. Wan, X.; Luo, Y. Measurement of the Development Level of Digital Economy and Its Impact Effect on Total Factor Productivity. *Reform* **2022**, 101–118.
36. Wang, D.; Peng, Z.; Li, L. Measuring and Evaluating the Integrated Development Level of Digital Economy and Agriculture in China. *Chinese Rur. Econ.* **2023**, 48–71, doi:10.20077/j.cnki.11-1262/f.2023.06.004.
37. Han, H.; Yang, D. Spatial Spillover Effects of Agricultural Industrial Agglomeration on the Growth of Agricultural Green Total Factor Productivity. *J. Ari. Land Resou. Envir.* **2023**, *37,* 29–37, doi:10.13448/j.cnki.jalre.2023.134.
38. Yang, X.; Qiao, C. Spatial Spillover Effects of Agricultural Industry Agglomeration on Agricultural Carbon Productivity: Based on the Regulatory Role of Fiscal Decentralization. *China Pop.,Res. Enviro.* **2023**, *33,* 92–101.
39. Cui, N.; Liu, Z.; Dong, J. Internal Logic and Long-term Mechanism Construction of Smart Agriculture for Reducing Losses of Grain Production. *Iss. Agric. Eco.* **2023,** 116–128, doi:10.13246/j.cnki.iae.2023.10.006.
40. Rural Revitalization Research Group of Wuhan University. Promote Rural Revitalization in All Respects and Ensure Food Security. *Fina. & Tra. Eco.* **2022**, *43,* 5–18, doi:10.19795/j.cnki.cn11-1166/f.20220505.009.
41. Zhou, H.; Liu, Y.; Guo, J. Analysis On FDI in Land of Developing Countries and Its Implications. *China Sof. Scie.* **2011**, 41–54.
42. Wang, X. To Ensure Food Security, We Need to Seize the Commanding Heights of Global Seed Industry Technology. *People's Tribune* **2020,** 64–65.
43. Guo, X.; He, Q.; Zhang, D. Application of Subjective and Objective Methods in Weights Determination of Synthetic Evaluation of Science and Technology. *Scie. Tech. Mana. Res.* **2012,** *32,* 64-67+71.
44. Huang, Q.; Yu, Y.; Zhang, S. Internet Development and Productivity Growth in Manufacturing Industry:Internal Mechanism and China Experiences. *China Indu. Econ.* 2019, 5–23, doi:10.19581/j.cnki.ciejournal.2019.08.001.
45. Yi, E.; Wang, J.; Zhu, J. Digital Economy, Resource Allocation Efficiency and High- Quality Development of Agriculture. *Mod. Fin. Econ.-J.Tianjin Univ. Fina. Eco.* **2023,** 20–37, doi:10.19559/j.cnki.12-1387.2023.12.002.
46. Nunn, N.; Qian, N. US Food Aid and Civil Conflict. *Am. Econ. Rev.* **2014**, *104*, 1630–1666, doi:10.1257/aer.104.6.1630.

Click here to access/download;Manuscript;Manuscript.docx

Article

The Impact of the Digital Economy on Food System Resilience: Evidence from China

Zhu Zheng[1,2], Wang Zhen [3]*, Liu Bin [2]*

[1] Jiangxi Daily, Nanchang 330006, China; 13970864503@163.com
[2] School of Economics & Management, Jiangxi Agricultural University, Nanchang 330045, China;
[3] School of Economics, Sichuan Agricultural University, Chengdu 611130, China;
* Correspondence: 18870478773@163.com(Z.W.); liubin@jxau.edu.cn (B.L.)

Abstract: Promoting a digital economy to empower food system resilience is particularly important for food security. Based on the panel data of 30 provinces in China from 2011 to 2021, this paper empirically examines the direct effect, the mechanism of action, and the spatial spillover effect of the resilience of a digital economy-enabled food system using a double fixed-effects model, mediated-effects model, and spatial econometric model. The results show the following: (1) The resilience of China's urban food system shows obvious spatial differences, but the overall trend in improvement requires attention. (2) The development of a digital economy has a facilitating effect on the level of resilience of the food system, and industrial agglomeration induces an intermediary effect. (3) The digital economy has a significant positive spatial spillover effect on the resilience of the food system; i.e., the digital economy can improve the resilience of the food system in the region and the neighboring regions. Accordingly, policy recommendations have been put forward to improve infrastructure construction and promote the development of digital villages; strengthen the construction of industrial agglomerations and promote the enhancement of the quality and efficiency of industries; and promote the development of regional linkages and build a solid foundation for food security.

Keywords: food systems resilience; digital economy; industrial agglomeration; mediating effect; spatial spillover effect

1. Introduction

Food security is "the most important thing in the country", and its major strategic role has a bearing on economic development and social stability; it also serves as an important foundation for national security. Since 2015, China has achieved sustained growth in total grain output, reaching a new level of 650 million tons. By 2023, it is projected to reach over 690 million tons, despite possessing less than 10% of the world's arable land to feed nearly 20% of the world's population. This is a remarkable feat and provides a powerful answer to the question of "who will feed China" [1]. Ensuring China's food security remains an urgent priority despite continuous improvements in the country's comprehensive food production efficiency. This is due to multiple constraints, such as domestic shortages of arable land, water, and other resources, and international geopolitical conflicts and a global economic slowdown [2]. The "2023 State of Food Security and Nutrition in the World" report by the United Nations highlights the need to enhance resilience to tackle food crises. Similarly, "Central Document No. 1 in 2023" emphasizes the importance of strengthening agriculture as a means of building a resilient industrial sector and a strong agricultural nation. Promoting the construction of a resilient food system is a path toward resisting and quickly recovering from risky shocks, shifting to a new growth path, achieving adaptive food development, and guaranteeing a balance between food production and

supply while eliminating the root causes of food insecurity [3]. However, the food industry is often associated with low-quality and inefficient production processes, making it challenging to enhance system resilience solely through its own efforts. In recent years, the digital economy has gradually penetrated all areas of the national economy and social development through cloud computing, big data, artificial intelligence, and other core high and new technologies. Its deep integration with agriculture provides new opportunities and challenges for enhancing the resilience of the food system. Empowering the food system with the digital economy will become an important direction for the future development of agriculture [4]. Furthermore, expediting the growth of the digital economy can strengthen the resilience of the food system. It is important to note that industrial agglomeration plays a crucial role in this process [5]. By leveraging the economic scale's externality, the specialization of the division of labor, and knowledge spillover, industrial agglomeration can enhance the efficiency of food production, improve the industry's competitiveness and risk management capabilities, and ultimately bolster the resilience of food systems [6]. Therefore, conducting an in-depth investigation into the impact and internal mechanisms of the digital economy on the resilience of the food system is of great theoretical and practical significance in guaranteeing national food security and comprehensively promoting rural revitalization.

There are three main types of literature related to the research theme of this paper. The first category is the construction of a food system resilience indicator system. Early scholars believe that food system resilience is mainly the resistance ability after coping with unexpected shocks (such as natural disasters, financial, and political crises) [7]. Some scholars have also proposed that food system resilience indicates the risk adaptive capacity that can systematically anticipate and withstand pressure [8]. Hao Aimin et al. (2022), on the basis of the three dimensions of resistance, adaptive capacity, and transformative capacity, constructed an index of China's food system resilience [9]. Other scholars have also studied food production resilience [10], cropland system protection resilience [11], and food security resilience [12], providing important references for this paper. The second category focuses on the study of the digital economy and its impact on food system resilience. Some scholars argue that digital village construction [9], digital financial inclusion [13], and digital finance [14] are key factors in enhancing the resilience of the food system. Others believe that the digital economy is the core element in empowering the high-quality development of the food industry [15], improving the resilience of the food supply chain [16], and increasing food security capacity [17]. This suggests that it could be a significant opportunity for fostering the growth of the digital economy and, in turn, strengthening the resilience of the food system. The third category studies the role of industrial agglomeration paths. One view is that the digital economy can mobilize resources, break through spatial and temporal limitations, and promote the optimization and integration of the food industry chain, thereby weakening the role of industrial agglomeration [18]. Another view is that the food industry has a higher degree of external risk and relies more on the knowledge spillover effect of the digital economy and the function of the rapid exchange of information to improve risk management and decision-making efficiency. The digital economy exacerbates the "siphon effect" of the city on resources, leading to the formation of a network system to jointly protect against risks. To improve risk management and decision-making efficiency, operators in the food industry, along with technology and knowledge experts, will be gathered in a specific region to form a network system to jointly resist risks. This promotes industrial agglomeration in the digital economy [19].

The literature review shows that previous studies are a valuable reference for this paper. However, there are still some shortcomings that need to be addressed: The previous literature on food security has primarily focused on theoretical and logical analysis, with few empirical studies from the perspective of food system resilience. Additionally, while China has achieved remarkable results in the development of the digital economy, its role in supporting and empowering high-quality industry development is becoming

more prominent, and there is a lack of research on the resilience of the digital economy and the food system. Furthermore, few studies have explored the intrinsic connection between the three, including industrial agglomeration. Therefore, this paper presents a theoretical analytical framework for exploring the resilience of the current food system in the context of the digital economy, industrial agglomeration, and spatial and temporal characteristics. The framework examines the impact effects, internal mechanisms, and spatial spillover effects of the food system's resilience. By analyzing these elements and proposing countermeasures, this paper aims to provide a scientific basis for ensuring China's food security.

2. Theoretical Analysis and Research Hypothesis

2.1. Direct Effects of the Digital Economy on Food System Resilience

The theory of network economics posits that the data network is a productive tool and that market behavior, information acquisition, decision making, and trading are all closely linked to the data network. This is particularly true in the digital economy era, where the trend toward networked food production and operational activities is increasingly apparent. The integration of substitution effects enhances the resilience of the food system in the digital economy. The integration of substitution effects enhances the resilience of the food system in the digital economy. This is achieved through networking, high-permeability effects, and sustainable practices, as well as information synergy, innovation, and creation effects. (1) It is important to consider the integration of substitution effects in this context. Urbanization has led to a loss of agricultural labor, resulting in the abandonment of arable land and a double predicament comprising a "shortage of resources" and "idle resources". The digital economy, with data as the core factor of production, differs from the traditional labor force and resource factors. Unlike traditional labor and other resource factors, the digital economy takes data as the core production factor. By replacing inefficient production factors, it increases factor inputs and promotes the effective use of resources. Additionally, the deep penetration and integration of the digital economy and the food industry can effectively empower the digital transformation and upgrading of the food industry. This improves the value of food industry factors and enhances the resistance of the food system [20]. (2) Synergy of information: The digital economy is characterized by networking through the Internet to realize the wide dissemination of information and data mining. It enables the effective management of "data islands" and overcomes data barriers, eliminating information asymmetry and externality problems. This allows food production and operation to utilize their own resources, achieving cross-regional and even cross-industry synergistic configurations and thus promoting decentralization in food production, circulation, sales, and services. This comprehensive extension of the food industry's chain optimizes the allocation of resources and enhances the adaptability of the food system [21]. (3) Innovation and creation effect: The development of the digital economy has opened a new industrial revolution round, reinventing not only the old organizational structure but also giving rise to new business models. This has promoted the transformation of the employment structure, improved the ecology of the employment market, and created a large number of new jobs for the food system, resulting in a significant creation effect [22]. The digital economy will also create diverse consumer demands and encourage constant reform and innovation in production and operations to adapt to market changes. This will inject new momentum for the transformation of the food system [23]. Therefore, we formulate the following hypothesis:

H1: Digital economic development can empower the enhancement of food system resilience.

2.2. Transmission Mechanisms of the Digital Economy to Enhance Food System Resilience

The theory of industrial agglomeration suggests that sharing resources, such as labor, capital, and information, can reduce production costs and promote the formation of industrial agglomerations [24]. The digital economy facilitates universal resource sharing through modern information networks, which contributes to the development of industrial agglomerations. First of all, China's grain industry agglomeration is still facing problems such as insufficient technological innovation ability, weak market competitiveness, small-scale agglomeration, etc. In the context of the digital economy, high permeability facilitates the efficient flow and precise configuration of resource elements. This enables the effective convergence of upstream and downstream industrial chains, as well as production, supply, and marketing, leading to efficient operations. It also helps enhance the stability and competitiveness of the industrial supply chain, attracting more investment and production operators to enter the field. This provides a key driving force for the agglomeration of the grain industry [19]. Secondly, the digital transformation of infrastructure and the innovation and creation effects of digital technology can broaden the financing, information, and technology acquisition channels of producers and operators. This effectively bridges the industrial development bias caused by differences in resource endowment, environmental regulations, and the level of informatization among regions, thus promoting the development of industrial agglomeration [25].

However, theories such as MAR externality, Porter externality, and Jacob externality highlight the positive impact of industrial agglomeration on economic resilience. MAR externality emphasizes the benefits of industry-specialized agglomeration, while Porter externality and Jacob externality focus on industry-related diversification [26]. This is also applicable to the resilience of the food system. Firstly, industrial agglomeration is led by infrastructure construction, which cannot be separated from facilities such as information systems, logistics and transportation, payment and settlement systems, and a healthy and relaxed development environment [14]. Secondly, industrial agglomeration can promote the construction of an information-sharing platform. This platform can facilitate mutual exchanges and information circulation among production operators in the agglomeration area. For example, it can provide information on market trends and competitor dynamics. This can help production operators better cope with market risks and competitive pressures. Additionally, it can help them adapt to market changes more quickly, thus improving the risk-adaptive capacity of the entire industry. Thirdly, due to the agglomeration economy, operators in the same or similar food production areas become more concentrated, leading to increased competition among producers. This drives continuous self-innovation and the improvement of service quality to enhance competitiveness. Industrial agglomeration provides a specialized external environment for relevant production operators in the region to optimize the allocation of innovation resources; moreover, it stimulates the dynamics of change for the resilience of the food system through the competitive effect [27]. Therefore, the hypothesis proposed is as follows:

H2: Industrial agglomeration contributes to enhancing the resilience of the food system enabled by the digital economy.

2.3. Spatial Spillover Effects of the Digital Economy on Food System Resilience

The theory of new economic geography suggests that knowledge and technology spillovers are directional and scope-specific. As a result, economic activities will eventually be evenly distributed across space [28]. Therefore, the impact of the digital economy on the resilience of the food system is not limited to a single city or region but spreads spatially and affects neighboring regions, resulting in a spatial impact effect [29]. The digital economy relies on the high permeability and network connectivity of digital platforms, driven by information and communication technologies. It reduces information asymmetry, breaks through regional constraints, and boosts the free flow of resource elements between regions. This leads to the Pareto-optimal state of the cross-regional realization of factors such as land, capital, labor, and technology. Simultaneously, the digital

economy facilitates the production and operation of online platforms for the food industry, enabling the development of a "dual-track drive" mechanism that links online and offline operations. This reduces the difficulty and cost of accessing information for the main production and operation bodies, enhancing production efficiency and cultivating the resilience of the food system to cope with potential impacts [30]. Moreover, particularly in the field of food production, neighboring regions share similar climate, water, and resource endowments. This results in a stronger role for inter-market and product competition, as well as substitution effects. Technical exchanges and access to information are also more convenient. To achieve a higher competitive position, it is important to actively absorb good practices from neighboring regions and promote synergistic development between different regions. This will improve the quality of food and enhance the resilience of the food system. Therefore, the following hypothesis is formulated:

H3: Digital economy-enabled food system resilience has a positive spatial spillover effect.

3. Model Construction, Variable Selection, and Data Sources

3.1. Model Construction

3.1.1. Benchmark regression model

In order to verify the impact effect of digital economy-empowering food system resilience, the following benchmark regression model is constructed:

$$\mathrm{FSR_{it}} = \alpha_0 + c\mathrm{Dig_{it}} + \beta_0 \sum \mathrm{Control_{it}} + \mu_i + \pi_t + \varepsilon_{it} \tag{1}$$

Equation (1) above shows the relationship between FSR_{it} and Dig_{it}, representing the resilience of the food system and the digital economy level of province i at time t, respectively. The set of control variables is denoted as $\sum Control_{it}$, while α_0 is a constant term. The correlation coefficients of the corresponding variables are represented by c and β_0, respectively. The province and year fixed effects are denoted by μ_i and π_t, respectively, and ε_{it} represents the random error term.

3.1.2. Mediation effects model

To further explore the role mechanism played by industrial agglomeration in the resilience impact of the digital economy-enabled food system, the following mediating effect model is constructed by drawing on the relevant research of Jiang Boat (2022) [31]:

$$\mathrm{Dig_{it}} = \alpha_1 + a\mathrm{Agg_{it}} + \beta_1 \sum \mathrm{Control_{it}} + \mu_i + \pi_t + \varepsilon_{it} \tag{2}$$

$$\mathrm{FSR_{it}} = \alpha_2 + c'\mathrm{Dig_{it}} + b\mathrm{Agg_{it}} + \beta_2 \sum \mathrm{Control_{it}} + \mu_i + \pi_t + \varepsilon_{it} \tag{3}$$

Equations (2) and (3) above use the term $\mathrm{Agg_{it}}$ to represent the degree of industrial agglomeration in province i at time t. The correlation coefficients for the corresponding variables are denoted by a, c', and b. The remaining equations are consistent with equation (1).

3.1.3. Spatial measurement models

(1) Spatial autocorrelation: Spatial autocorrelation is commonly measured using the global Moran index I. This index ranges from -1 to 1, with values greater than 0 indicating a positive correlation, meaning high–high or low–low neighboring, and values close to 0 indicating no spatial autocorrelation. The formula for Moran index I is as follows:

$$I = \frac{\sum_{i=1}^{n}\sum_{j=1}^{n} W_{ij}(x_i - \overline{x})(x_j - \overline{x})}{s^2 \sum_{i=1}^{n}\sum_{j=1}^{n} W_{ij}} \tag{4}$$

In the above equation (4), $S^2 = \frac{\sum_{i=1}^{n}(x_i - \overline{x})^2}{n}$ is the sample variance, W_{ij} is the spatial weight matrix, and $\sum_{i=1}^{n}\sum_{j=1}^{n} W_{ij}$ is the sum of all spatial weights; in this paper, we utilize spatial proximity matrix; x_i and x_j denote the values of the indicators of provinces i and j, respectively.

(2) Spatial Durbin model: In order to explore the spatial spillover effects of the digital economy and the resilience of the food system, the spatial Durbin model is established as follows:

$$\mathrm{FSR_{it}} = \theta_0 + \lambda W\mathrm{FSR_{it}} + \delta_1 DIg_{it} + \theta_1 WDIg_{it} + \delta_2 \sum \mathrm{Control_{it}} + \theta_2 W \sum \mathrm{Control_{it}} + \mu_i + \pi_t + \varepsilon_{it} \quad (5)$$

In Eq. (5), λ is the spatial correlation coefficient of the resilience of the food system; $W(\cdot)$ is the spatial lag variable; δ and θ are the coefficients of each variable; the rest is consistent with Eq. (1).

3.2. Variable Selection

3.2.1. Explanatory variable: food system resilience (FSR)

Based on the theoretical analysis and relevant literature [9,13,14,32], we selected 17 indicators to construct the resilience index system for the food system. The system is constructed from three dimensions: resistance, adaptability, and changeability (refer to Table 1). To avoid measurement bias caused by inconsistent indicators, this paper uses the entropy method to standardize indicators and measure food system resilience objectively by assigning weights to each indicator. This is then integrated into a composite index, drawing on the research of Zhu Xi'an et al. (2015) [33].

Table 1. Index system of food system resilience.

Primary index	Secondary index	Index calculation method and unit	Property	Weight
Capacity for resist	Cultivated land per capita	Cultivated land area/number of persons employed in the primary sector (ha/person)	+	0.102
	Labor force level	Number of people employed in primary industry/total employment (%)	+	0.037
	Level of fixed asset investment	Investment in agricultural fixed assets / Gross regional product (%)	+	0.054
	Grain production per capita	Total grain production/total number of people in the province (kg/person)	+	0.082
	Food production price index	Price of grain production in the current/base period (%)	-	0.016
	Grain production level	Grain production/grain sown area (t/ha)	+	0.022
Capacity for capacity	Intensity of pesticide use	Pesticide use / area sown with crops (t/ha)	-	0.001
	Degree of disaster	Crop damage area / crop sown area (%)	-	0.005
	Fertilizer use intensity	Amount of agricultural fertilizer applied (pure) / area sown with crops (t/ha)	-	0.016
	Intensity of agricultural film use	Agricultural plastic film use/crop sown area (t/ha)	-	0.008
	Replanting index	Grain sown area/cultivated area (%)	+	0.028
	Agricultural value add index	Agricultural value add index (%)	+	0.005
	Per capita rural electricity consumption,	Rural electricity consumption/rural population (10 thousand* kW·h/person)	+	0.297
Capacity for change	Degree of mechanization	Total power of agricultural machinery/area sown with crops (10*kW)/ha	+	0.047
	Level of agricultural development	Gross agricultural output value / gross regional product (%)	+	0.052
	Level of Expenditure on Agricultural Research	Local fiscal expenditure on science and technology* gross agricultural output value/gross regional product (CNY billion)	+	0.088
	Level of agricultural technicians	Persons obtaining national vocational qualification certificates in the grain industry/number of employees in primary industry (%)	+	0.138

3.2.2. Core explanatory variable: level of digital economy (Dig)

The literature on constructing a digital economy index system primarily focuses on digital economy carriers, industrial digitization, and digital industrialization [34–36]. This paper selects 19 indicators for measurement (Table 2). The steps for measuring the comprehensive index of the digital economy level using the entropy value method are consistent with those for measuring the food system resilience index.

Table 2. Index system of the digital economy.

Primary index	Secondary index	Index calculation method and unit	Property	Weight
Carrier of digital economy	Cell phone penetration	Number of cell phone subscribers per 100 people (households/100 people)	+	0.011
	Internet penetration	Number of Internet users as a proportion of resident population (%)	+	0.011
	Breadth of information transmission	Density of fiber-optic cable lines (km/km2)	+	0.060
	Signal coverage breadth	Density of cell phone base stations (pcs/km2)	+	0.077
	Internet broadband infrastructure	Density of Internet broadband access ports (pcs/km2)	+	0.081
	Strength of investment in digital services	Per capita fixed asset investment in information transmission, computer services and software industry (CNY/person)	+	0.027
Digital industrialization	Development level of post and telecommunications industry	Total telecommunication business per capita (CNY/person)	+	0.050
		Total postal services per capita (CNY/person)	+	0.074
		Volume of express delivery (ten thousand pieces)	+	0.114
	Development level of electronic information manufacturing industry	Revenue of electronic information manufacturing industry (CNY)	+	0.094
		Number of enterprises in electronic information manufacturing industry (nos.)	+	0.092
	Software and information technology service industry	Software business revenue (CNY)	+	0.090
		Number of employees in information transmission, software and information technology services (ten thousand)	+	0.054
Digitalization of industries	Development level of enterprise digitization	Number of websites owned by enterprises (number)	+	0.044
		Share of enterprises with e-commerce transaction activities (%)	+	0.016
		E-commerce transaction volume (CNY billion)	+	0.069
	Development level of digital inclusive finance	Digital finance coverage breadth index	+	0.013
		Depth of digital finance usage index	+	0.012
		Digital finance digitization degree	+	0.010

3.2.3. Mediating variable: food industry agglomeration (Agg)

Drawing on Han Haibin et al. (2023) and Yang Xiuyu et al. (2023) [37,38], the degree of food industry agglomeration is measured via locational entropy, which overcomes the influence of regional scale differences and other effects, and it measures the degree of agricultural agglomeration more accurately with the following formula:

$$Agg_{it} = (A_{it} \ / \ G_{it})/(A_t \ / \ G_t \ ) \quad (4)$$

Equation (8) defines Agg_{it} as the degree of food industry agglomeration in province i at time t, which is proportional to its value. A_{it} and G_{it} represent the total output value of agriculture, forestry, livestock, and fishery, and the gross regional product of region i in year t, respectively. $A_t$and G_t represent the total output value of agriculture, forestry, livestock, and fishery and the gross domestic product of the entire country in year t, respectively.

3.2.4. Control variable

To ensure the resilience of the food system, the following control variables are primarily considered to control the impact of other variables:

1) The level of informatization is expressed by the ratio of the total amount of post and telecommunication services to GDP.
2) Employment density is represented by the ratio of the number of employees in the primary industry to the area of administrative divisions.
3) Foreign direct investment is expressed as the ratio of foreign direct investment to GDP after multiplying the current USD/CNY exchange rate.
4) The level of development in the technology market can be measured using the ratio of technology market turnover to GDP.
5) The degree of industrialization can be measured using the ratio of the industrial added value to GDP.
6) The degree of openness to the outside world can be measured using the ratio of the value of imports and exports of goods, multiplied by the USD/CNY exchange rate of the current year, to GDP.

3.3. Data sources

Considering the availability of data, this paper uses 30 provinces in China (except Tibet, Hong Kong, Macao, and Taiwan) from 2011 to 2021 as the research object, and the data are mainly obtained from the China Rural Statistical Yearbook, China Statistical Yearbook, EPS database, and statistical yearbooks and statistical bulletins of the corresponding years in each province and city. Linear interpolation is used to add some missing values. The descriptive statistics of each variable are shown in Table 3.

Table 3. Descriptive statistics of variables.

Variable	Symbol	Mean	SD	Min	Max
Resilience of food system	FSR	0.195	0.058	0.114	0.463
Digital economy	Dig	0.119	0.108	0.010	0.647
Industrial agglomeration	Agg	1.185	0.623	0.049	3.467
Informatization level	Inf	0.060	0.055	0.014	0.290
Employment density	Emp	0.026	0.039	0.000	0.217
Foreign direct investment	For	0.019	0.015	0.000	0.080
Technology market development level	Tec	0.016	0.029	0.000	0.175
Degree of industrialization	Ind	0.321	0.082	0.101	0.556
Degree of opening up	Open	0.265	0.291	0.008	1.548

4. Results Analysis and Discussion

4.1. Spatial and temporal characterization of food system resilience

This paper examines the spatial and temporal characteristics of China's food system resilience by estimating the kernel density map through plotting. Figure 1 shows that the center of kernel density moves to the right each year, indicating an improvement in the resilience of the food system and effective food security guarantee. The distribution curves of each year indicate that the resilience of the food system in most regions of China is still at a middle to lower level. The curves also show a unipolar phenomenon during the examination period. These findings suggest that the food system in China needs improvement. Additionally, the curves for each year exhibit a clear "right trailing" pattern, suggesting that the resilience of the food system during the study period is unipolar. Overall, China's food system has significantly improved in recent years. However, there are still substantial variations in the resilience levels across different regions, with a larger proportion of regions having lower resilience levels. Therefore, there is a need to strengthen the resilience level of the food system.

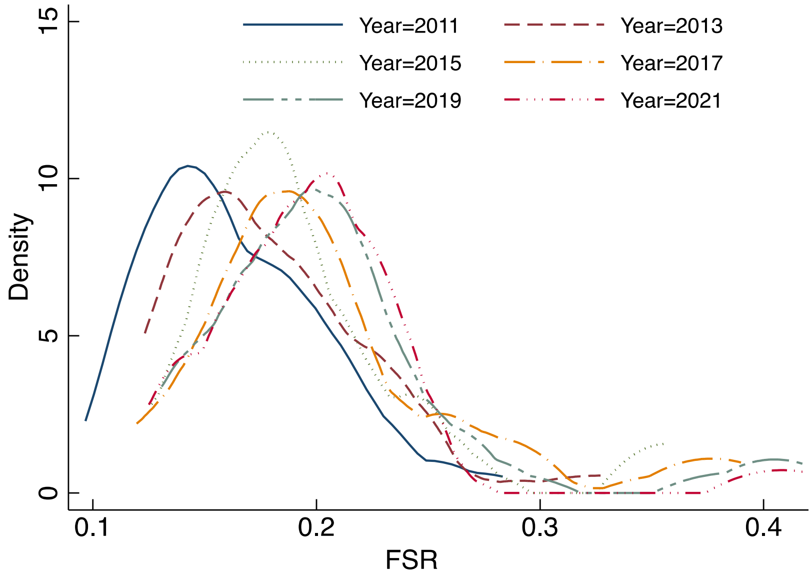

Figure 1. Spatial and temporal characteristics of the resilience of China's food system.

4.2. Analysis of baseline regression results

Prior to conducting empirical analyses, the variables were assessed for multicollinearity. The results indicate that Max-VIF is 3.39, which is significantly lower than the threshold of 10, thus indicating the absence of multicollinearity. The impact of the digital economy on the resilience of the food system was analyzed using the baseline regression model. The results are presented in Table 4, with columns (1) to (4) showing the results of no fixing, province fixing, year fixing, and province and year fixing after the introduction of the control variables, respectively. The results indicate that the regression coefficients of the digital economy are all positive and pass the significance test at the 10% level when the province and year effects are fixed. This suggests that the digital economy significantly enhances the level of food system resilience. Possible reasons for this include the use of digital technologies such as big data and intelligent control to achieve precision planting, intelligent fertilizer application, and automated irrigation. This enhances food resistance through precision and intelligent agriculture. It promotes the rational use of natural resources, such as land and water, and supports sustainable food production. Additionally, it enhances the adaptive capacity of the food system. The promotion of digital transformation in the food industry chain encourages food operating entities to accelerate technological progress, improve technological efficiency, and ultimately enhance the resilience of the food system [14].

When controlling for province and year effects, the level of informatization showed a significant positive impact on the resilience of the food system. This may be due to its ability to facilitate information sharing, reduce information asymmetry, improve coordination within the food system, enhance its ability to cope with external shocks, and ultimately increase its resilience [39]. Additionally, the level of employment density also had a significant positive impact on the resilience of the food system at the 1% level. The resilience of the food system was found to have a statistically significant positive effect at the 1% level in areas with high employment density. This may be due to the fact that such areas are conducive to the better management of food producers and operators, improve the quality and skill levels of food producers and operators, and have a better ability to cope with external shocks and risks in the food industry [40]. Foreign direct investment was found to have a statistically significant negative effect on the resilience of the food system at the 1% level. Foreign direct investment may lead to increased market competition for domestic food production operators, resulting in the use of agricultural chemicals

and other means of production to enhance market competitiveness. This may not be conducive to the stabilization of food system resilience [41]. Statistical analyses show that technological market development has a positive and significant impact on food system resilience at the 5% level [42]. This may be due to the fact that technological market development enables food production operators to quickly adopt new technologies, modes, and business models, which in turn improves production efficiency, product quality, and added value in the food industry. As a result, the resilience of the food system is enhanced.

Table 4. Descriptive statistics of variables.

**Variable**	**(1)**<br>**FSR**	**(2)**<br>**FSR**	**(3)**<br>**FSR**	**(4)**<br>**FSR**
Dig	0.164***（0.028）	0.102**(0.047)	0.121***(0.038)	0.111*(0.063)
Inf	0.003（0.056）	0.077**(0.032)	-0.147(0.115)	0.255***(0.084)
Emp	1.012***（0.108）	2.326***(0.641)	0.987***(0.119)	2.384***(0.637)
For	-0.336*(0.184)	-0.431***(0.131)	-0.362*(0.192)	-0.462***(0.140)
Tec	-0.240***（0.091）	0.444**(0.193)	-0.275***(0.092)	0.462**(0.192)
Ind	-0.081*(0.045)	-0.092**(0.044)	-0.070(0.044)	-0.068(0.050(
Open	-0.112***(0.012)	-0.022(0.024)	-0.096***(0.017)	-0.016(0.025)
Year	No	No	Yes	Yes
province	No	Yes	No	Yes
Sample	330	330	330	330
R^2	0.293	0.863	0.302	0.872

Note: Robust standard error in brackets: * * *, * *, and * indicate significant at 1%, 5%, and 10% levels, respectively. The same below.

4.3. Robustness Test

In order to verify the reliability of the findings, a robustness test was conducted (Table 5).

4.3.1. Adjustment of the sample period

The sample period is adjusted to 2018-2021 and 2011-2019, respectively. The reasons are as follows: In 2017, the digital economy was written in the government work report for the first time, and the development of the digital economy opened a new chapter; this policy factor may affect research conclusions, and only the sample after the policy's release is retained for analyses. The regression results are shown in column (1). In 2020, there was a large impact of the New Crown Epidemic on both the development of the digital economy and food production, and in order to exclude the impact of this major environmental change, only the samples before 2020 were analyzed. The regression results are shown in column (2). Columns (1) and (2) show that after adjusting for the sample period separately, the digital economy still passes a positive significance test at the 10% level for food system resilience.

Table 5. Robustness test.

**Variable**	**(1)**<br>**FSR**	**(2)**<br>**FSR**	**(3)**<br>**FSR**
Dig	0.214*（0.120）	0.127*(0.069)	— —
Dig#	— —	— —	0.181**（0.084）
Control variable	Yes	Yes	Yes
Year	Yes	Yes	Yes
Province	Yes	Yes	Yes
Sample	120	270	330
R^2	0.959	0.886	0.865

4.3.2. Replacement of core explanatory variables

The entropy value method was used in the previous section to measure the composite index, and the CRITIC and entropy value methods are both objective weighting methods. The CRITIC method avoids the problem of weighting when dealing with the comprehensive evaluation of multiple indicators, but it also takes into account the effect of correlations on the weight of indicators and eliminates the effect of correlation indicators on the weight during weighting [43]. Using this method to re-measure the level of digital economy development and define the new variable as "Dig#", the regression results are shown in column (3) of Table 5. The results show that the estimation of the digital economy with respect to the resilience of food systems is still significant after using the CRITIC method to measure the digital economy. This indicates that the development of the digital economy increases the level of resilience of the food system.

4.4. Endogeneity Test

Despite controlling for as many key variables as possible in this paper, endogeneity may still be an issue. Due to the large number of factors affecting food system resilience, it is not possible to completely prevent, for example, the occurrence of omitted variables and bidirectional causality, which could lead to biased estimation results. Since there is a possibility that the level of food system resilience in turn affects the development of the digital economy, regions with faster digital economy development have a stronger comprehensive food production capacity and risk resistance capacity per se and higher food system resilience, and it is not possible to truly identify whether the increase in food system resilience is caused by the development of the digital economy [15]. Therefore, in this study, based on the experience of Huang Qunhui et al. (2019) [44] and the availability of data, the number of landline phones at the end of 1990 (in 10,000 households) in each region is selected as the instrumental variable, and a regression test is conducted using the two-stage least squares method.

The main considerations for the choice of this instrumental variable are that, on the one hand, the core of the development of the digital economy cannot be separated from the development of the Internet, and Internet technology is a continuation of the popularization of fixed-line telephony; i.e., The popularization of fixed-line telephony is one of the important infrastructures for the development of the digital economy, which satisfies the relevance requirement [45]. On the other hand, with the rapid application of modern communication technology, the role that traditional communication tools, such as fixed-line telephony, play in the development of today's food system resilience is minimal, and this satisfies the exclusivity requirement [9]. It is worth noting that the number of landline telephones at the end of 1990 comprises cross-sectional data, while the sample in this paper comprises balanced panel data. Therefore, based on Nunn and Qian (2014) [46], the cross-multiplier term between the number of Internet access instances in the previous year and the number of landline telephones at the end of 1990 is constructed as an instrumental variable (IV) for the digital economy index of the current period. The estimation results are presented in Table 6. The results in column (1) indicate that the instrumental variable has a significant positive effect on the digital economy, and the results in column (2) show that after applying the two-stage least squares method to mitigate the endogeneity problem, the digital economy still passes the positive significance test on the resilience of the food system and is significant at the 5% level, which is basically the same as the results of the benchmark regression. In addition, the results of the weak instrumental variable test show that the F-statistic is 241.435, which is significantly greater than the critical value of 10; therefore, there is no weak instrumental variable problem. Accordingly, this indicates that the development of the digital economy determines the improvement of the resilience of the food system, which verifies hypothesis H1.

Table 6. Results of the endogeneity test.

Variable	Instrumental variable method	
	（1）Dig	（2）FSR
IV	6.38e-08***（4.12e-09）	— —
Dig	— —	0.103**（0.051）
Control variable	Yes	Yes
Year	Yes	Yes
Province	Yes	Yes
F-value		241.435
Sample	330	330
R^2	0.955	0.895

4.5. Endogeneity Test

To further test the mediating effect of food industry agglomeration on the impact of the digital economy on food system resilience, the mediating effect model constructed in the previous section is used for validation (Table 7). As observed in Table 7, in column (1), the digital economy on food industry agglomeration passed the positive significance test at the 1% level, indicating that the development of the digital economy promotes the food industry's agglomeration level. In column (2), after the introduction of the digital economy variable, both the digital economy and food industry agglomeration on food system resilience passed the positive significance test at the 1% level, indicating that food industry agglomeration enhances the level of food system resilience. Accordingly, this indicates that the digital economy can enhance the resilience level of the food system by promoting the degree of food industry agglomeration. Food industry agglomeration plays a mediating effect on the impact of the digital economy on the resilience of the food system.

Table 7. Results of the endogeneity test.

Variable	（1）Agg	（2）FSR
Dig	1.912***(0.474)	0.235***(0.066)
Agg		0.065***(0.008)
Control variable	Yes	Yes
Year	Yes	Yes
province	Yes	Yes
Sample	330	330
R2	0.955	0.895

In order to test the reliability of the above mediated effect results, the self-sampling test method (bootstrap) was used to conduct a robustness test of the above results, and the number of bootstrap replicate samples was set at 500. The results are shown in Table 8. The results show that the coefficients of the indirect and direct effects are positively significant at the 1% level, and the 95% confidence interval does not include 0, which is basically consistent with the above conclusion. The mediating effect of the agglomeration of the food industry is again verified. Hypothesis H2 is therefore confirmed.

Table 8. Tests the mediation effect based on the bootstrap method.

Influence type	Coefficient	SD	P-value	[illegible]% confidence interval
Indirect effect	0.068	0.019	0.000	0.036
Direct effect	0.232	0.036	0.003	0.174

4.6. Analysis of Spatial Spillover Effects

4.6.1. Spatial autocorrelation test

Before carrying out the spatial econometric model, this paper adopts the global Moran's index (Moran's *I*) to calculate the spatial correlation between digital economy and food system resilience in each region under the spatial proximity matrix, and the results are shown in Table 9. The results show that Moran's index of digital economy and food system resilience for each year passed the positive significance test, indicating that there is an obvious positive spatial correlation between the digital economy and food system resilience in our region during the study period.

Table 9. Spatial autocorrelation test.

Year	Digital economy		Food system resilience	
	**Moran's I**	**Z-value**	**Moran's I**	**Z-value**
2011	0.261**	2.488	0.463***	4.081
2012	0.300***	2.813	0.441***	3.951
2013	0.266**	2.536	0.408***	3.781
2014	0.262**	2.503	0.433***	4.072
2015	0.281***	2.660	0.299***	2.890
2016	0.272**	2.592	0.268**	2.583
2017	0.251**	2.421	0.240**	2.345
2018	0.227**	2.240	0.153*	1.695
2019	0.216**	2.160	0.269***	2.699
2020	0.217**	2.162	0.233**	2.415
2021	0.223**	2.221	0.163*	1.835

We then plotted a Moran scatterplot of the digital economy and food system resilience in 2021 using Stata16 software (Figure 2). The results show that most areas are distributed in the high–high and low–low quadrants, indicating the spatial clustering of the digital economy and food system resilience and initially validating the spillover effect between the two.

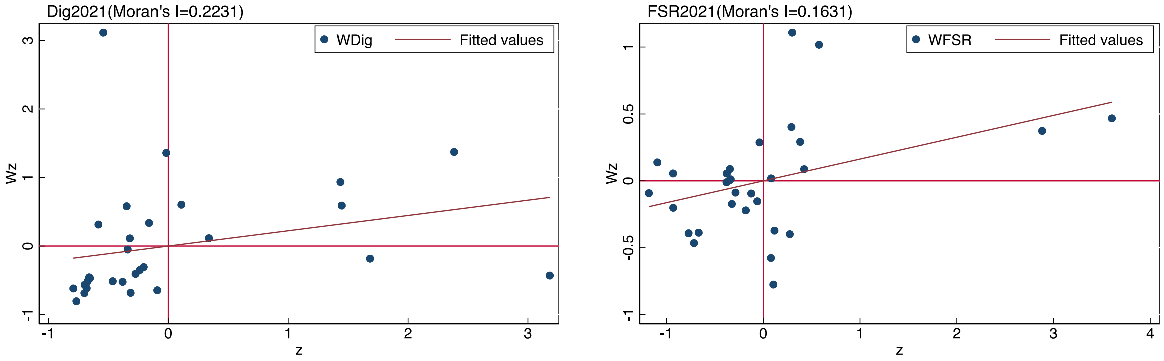

Figure 2. Moran's *I* scatter plot of the digital economy and food system resilience in 2021.

4.6.2. Spatial spillover effects

Before conducting empirical analyses, it is necessary to determine which spatial econometric model to select, and the results of the LM, LR, and Hausman tests show that the spatial Durbin model is superior to the spatial lag model and the spatial error model. In order to compare the test results, the regression results of the spatial Durbin model (SDM) and the spatial autoregressive model (SAR) were reported simultaneously, following Yi Enwen (2023) [45] (Table 10). The results show that the effect of the digital economy on local food system resilience (direct effect), the effect of the local digital economy on food system resilience in neighboring regions (indirect effect), and the total effect (the sum of direct and indirect effects) all passed the positive significance test. On the one hand, the development of the digital economy can promote the digital transformation of the local

food industry chain. It can improve the efficiency and resilience of all aspects of food production, circulation, and consumption; for example, the digital economy can help food production operators more accurately manage arable land and optimize the structure of grain cultivation, etc. Moreover, the optimization of logistics can be promoted. On the other hand, the development of the local digital economy can attract the inflow of talent and resources from neighboring regions and promote the exchange and sharing of digital technology, the construction of digital infrastructure, and the development of digital industrialization in neighboring regions, which in turn enhance the resilience of the food system in neighboring regions. Therefore, hypothesis H3 is supported.

Table 10. Test results of the spatial spillover effect.

Variable	SDM	SAR
Digital economy	0.371***(0.037)	0.381***(0.035)
W×Dig	0.060*(0.044)	
rho	0.177**(0.083)	0.211***(0.076)
sigma2_e	0.000***(0.000)	0.000***(0.000)
Direct effect	0.378***(0.039)	0.387***(0.037)
Indirect effect	0.151**(0.071)	0.103**(0.046)
Total effect	0.530***(0.058)	0.491***(0.065)
Control variable	Yes	Yes
Year	Yes	Yes
Province	Yes	Yes
Sample	330	330
Within R^2	0.366	0.458

5. Conclusions and Policy Recommendations

5.1. Conclusions

This study takes the panel data of 30 provinces in China from 2011 to 2021 as a sample; constructs an index system of China's digital economy and food system resilience; draws a kernel density map to analyze the spatial and temporal differences in the characteristics of China's food system resilience; uses the double fixed-effects model to test the impact effect of the digital economy on food system resilience; and applies the mediating effects model to clarify the transmission mechanisms of the digital economy on food systems through the industry. Using the mediating effects model, we clarified the transmission mechanism of agglomeration in the digital economy with respect to food system resilience and used the spatial measurement model to explore the spatial spillover effect of the digital economy on food system resilience. The results of the study show that, firstly, China's current food system resilience level in the time series shows a stable growth trend, but with respect to the spatial difference, it is obvious that the level of food system resilience still needs to be strengthened as the region's low levels accounted for a large proportion of the narrowing of regional differences. Second, the digital economy plays an important role in promoting the resilience of the food system, and this conclusion is still valid after considering the endogeneity and robustness test. Therefore, in the process of promoting the resilience of the food system driven by the digital economy, the "digital dividend" effect plays an important role, which provides empirical evidence for ensuring China's food security and comprehensively promoting the rural revitalization strategy. Third, promoting industrial agglomeration is an inherent mechanism of the digital economy to enhance the resilience of the food system. Driven by the digital economy, resource elements in the agglomeration area will be revitalized, and production capacities can be released to promote the digital transformation of the food industry, which in turn further enhances the resilience of the food system. Fourth, there is a positive spatial spillover ef-

fect: i.e., the development of the digital economy at the provincial and national food system resilience levels has an important role in promoting the resilience of the food system in neighboring provinces and cities; simultaneously, there is a significant positive impact on the resilience of the food system.

5.2. Policy Recommendations

First, we recommend the following: improve infrastructure construction and promote the development of digital villages. To make up for the lack of rural digital infrastructure, focus should be placed on supporting the digital transformation of rural mobile communication base stations and terminals and accelerating the construction of convenient, high-speed, and smooth mobile communication networks, especially with respect to providing 5G networks in remote rural areas. Moreover, the application of 5G in rural areas should be further explored, which includes improving the perception, collection, screening, and transmission of data and information; creating a digital information "highway" in the grain industry; and consolidating the foundation of digital economy development on all fronts. In addition, it is necessary to enhance the digitalization awareness of grain farmers and their ability to apply information technology, and the following principle should be taken as the starting point to promote the development of intelligent agriculture for grain farmers: "promoting the development of intelligent agriculture, promoting the integration and application of information technology and agricultural machinery and agronomy". In addition, the following ideologies are recommended: the ideological awareness of intelligent agricultural development and practical food and agriculture skills should be enhanced; digital agriculture and skill training for local food and agriculture should be carried out; the high-quality development of food should be ideologically established; digital agricultural literacy should be effectively cultivated; the cultivation of new vocational digital agriculture should be accelerated.

Second, the construction of industrial agglomeration should be strengthened, and the quality and efficiency of food should be promoted. Industrial agglomeration plays an important role in promoting the resilience of the digital economy-based food system. On the one hand, it is necessary to improve market-oriented development to attract the development of upstream and downstream production and operation clusters. Simultaneously, it is also necessary to strengthen the drive for scientific and technological innovation, such as the implementation of large-scale innovation projects with respect to regional characteristics and field advantages. Moreover, investments in research and development and talent team-building efforts should be increased in order to encourage agriculture-related college students, postgraduate students, and agricultural technicians to carry out grassroots fieldwork. On the other hand, we should create a good development environment; focus on creating a market-oriented international business environment that follows the rule of law; improve financial literacy; avoid blindly attracting foreign investment; and focus on changes from "quantity" to "quality" at the agricultural enterprise scale. The quality and efficiency of industrial development will be enhanced through multiple channels and initiatives, and the resilience of the food system will be gradually improved.

Third, regionally linked development should be promoted to lay a solid foundation for food security. The results show that the resilience of the food system in each region has a wide range of linkages. Thus, each region should adapt to local conditions; formulate a food development strategy that is highly compatible with its own actual situation; and focus on promoting measures to improve the quality of specialty foods, creating local specialty food brands and realizing the development of a new business model for the specialty food industry. On the other hand, it is necessary to strengthen exchanges and cooperation in the food industry among cities, promote the construction of a unified national market, eliminate the "local" thinking of some local governments, and promote the development of the food industry in a manner that effectively connects the "hardware" and "software" across regions. The effective convergence of "hardware" and "software" is a

method for promoting platform building, industrial integration, public services, talent exchange, etc., in administrative regions.

5.3. Main Limitations

This paper is dedicated to exploring the relationship between the digital economy and food system resilience, and empirical methods have been used to carry out useful explorations. However, due to datum limitations and the author's lack of experience, there is still room for improvement and exploration. Firstly, although the scientificity and completeness of the indicators for measuring the digital economy and food system resilience have been taken into account as much as possible, the complexity of food production activities and the availability of data inevitably lead to the omission of variables, which in turn imparts a certain degree of bias in the analysis's results. Secondly, although the scale of the study has a certain degree of depth and the data of the study are relatively fine, gaps still exist compared to studies carried out at the county or township scale; further in-depth research will be carried out with respect to these factors.

Author Contributions: Conceptualization, Z.Z. and Z.W.; data curation, Z.W.; formal analysis, Z.Z.; funding acquisition, Z.Z. and B.L.; methodology Z.Z., Z.W. and B.L; software, Z.W. and Z.Z.; writing—original draft, Z.Z. and B.L.; writing—review and editing, Z.Z. and Z.W.; Z.Z. and Z.W. contributed equally to this work and should be regarded as co-first authors. All authors have read and agreed to the published version of the manuscript.

Funding: This research was funded by the National Natural Science Foundation of China (grant no. 71563016), Key Research Base Project of Humanities and Social Sciences in colleges and universities of Jiangxi Province (JD21080), the Jiangxi Postgraduate Innovation Special Fund Project (YC2022-s415), and the Jiangxi Agricultural University School of Economics and Management 2022 Postgraduate Innovation Special Fund Project (grant no. JG2022008).

References

1. Wang, K. Achievements and Experiences of China's Food Security in the New Era. *Reform* **2023**, *9*, 99–109.
2. Luo, B. Seed industry revitalization and food security: A review. *J. Sou. China Agric. Uni.* **2023**, *44*, 827–836.
3. Kang, L. Food security in Central Asia under the new situation：Current situation，causes and path selection. *J. China Agric. Uni.* **2024**, *29*, 258–270.
4. Zhang, Y.; Wang, M.; Liao, Y. Rice Industrialization from the Perspective of Ensuring National Food Security: Based on Monitoring Data of 2,225 Rice Enterprises. *Reform* **2022**, *11*, 119–129.
5. Gao, W. Mechanism of Industrial Agglomeration Driving High-quality Development of Grain. *J. Sou. China Agric. Uni. (Soc. Sci. Edi.)* **2021**, *20*, 80–94.
6. Ren, S.; Li, L.; Han, Y.; Hao, Y.; Wu, H. The Emerging Driving Force of Inclusive Green Growth: Does Digital Economy Agglomeration Work? *Bus. Strateg. Environ.* **2022**, *31*, 1656–1678, doi:10.1002/bse.2975.
7. Tendall, D.M.; Joerin, J.; Kopainsky, B.; Edwards, P.; Shreck, A.; Le, Q.B.; Kruetli, P.; Grant, M.; Six, J. Food System Resilience: Defining the Concept. *Glob. Food Secur.-Agric.Policy* **2015**, *6*, 17–23, doi:10.1016/j.gfs.2015.08.001.
8. Coopmans, I.; Bijttebier, J.; Marchand, F.; Mathijs, E.; Messely, L.; Rogge, E.; Sanders, A.; Wauters, E. COVID-19 Impacts on Flemish Food Supply Chains and Lessons for Agri-Food System Resilience. *Agric. Syst.* **2021**, *190*, 103136, doi:10.1016/j.agsy.2021.103136.
9. Hao, A.; Tan, J. Impact of Digital Rural Construction On Food System Resilience. *J. Sou. China Agric. Uni. (Soc. Sci. Edi.)* **2022**, *21*, 10–24.
10. Jiang, H.; Chen, Y.; Liu, Z. Spatiotemporal Pattern and Influencing Factors of Grain Production Resilience in China. *Econo. Geogr.* **2023**, *43*, 126–134, doi:10.15957/j.cnki.jjdl.2023.06.013.

11. Hu, L.; Guo, J.; Ou, M.; Qian, H. The "Rigidity-Elasticity-Resilience" of Systematic Farmland Protection: Theoretical Framework and Implementation Path. *Planners* **2023**, *39*, 7–13.
12. Qing, P; Deng, X.; Min, S. Food Security Resilience and Risk Management Strategy in China in the Context of "Dual Circulation". *Strat. Stu. CAE* **2023**, *25*, 26–38, doi:10.15302/J-SSCAE-2023.04.002
13. Li, M.; Chen, D; Yu, G. Digital inclusive finance enhances food system resilience: from the perspective of spatial spillover effect. *Chin. J. Agric. Reso. Reg. Plan.* **2023,** *6*, 1–13.
14. Wang, Y.; Ye, J.; Cao, J. Study on the Mechanism and Effect of Digital Finance Improving the Resilience of Grain System. *Econ. Sur.* **2023**, *40*, 48–60, doi:10.15931/j.cnki.1006-1096.2023.05.003.
15. Zuo, X.; Ye, L.; Ma, J. Empirical Test on the Impact of Digital Economy on High-quality Development of Grain Industry. Stati. & Dec. **2023**, *39*, 36–41, doi:10.13546/j.cnki.tjyjc.2023.22.006.
16. Ma, J.; Li, G.; Han, D. Digital economy empowers food supply chain resilience: path of action and policy orientation. Soc. Sci. Xinjiang **2023**, *1*, 46–54, doi:10.20003/j.cnki.xjshkx.2023.01.006.
17. Qi, Y.; Wang, Y.; He, Q. Spatio-temporal Evolution and Impact Analysis of Digital Economy Development and Food Security Capacity in the Yangtze River Economic Belt. *Rural Economy* **2023**, 48–58.
18. He, P.; Tan, C. E-Commerce and Rural Economic Resilience: Empirical Evidence Based on the "Comprehensive Demonstration E-commerce into Rural Areas" Policy J. *Zhongnan Uni. Econ. Law* **2023**, 97–108, doi:10.19639/j.cnki.issn1003-5230.2023.0009.
19. Kou, D.; Zhang, C.; Zhong, W. The impact of digital economy on industrial agglomeration and its empirical analysis. *South. Aca. Rese.* **2023**, 147–157, doi:10.13658/j.cnki.sar.2023.06.009.
20. Ouyang, R. The Institutional Logic of Data Element Circulation. *Frontiers* **2023**, 13–27, doi:10.16619/j.cnki.rmltxsqy.2023.06.002.
21. Chen, M.; Zhang, X. The Scale Measurement and Productivity Analysis of China's Digital Economy. *J. Quan. Tech. Econ.* **2022**, *39*, 3–27, doi:10.13653/j.cnki.jqte.2022.01.004.
22. Su, Y.; Lu, J. Development of the Digital Economy,Job Tasks and Job Changes. *Popula. J.* 2023, 45, 69–82, doi:10.16405/j.cnki.1004-129X.2023.05.006.
23. Guo, K.; Liu, C. Platform Anti-Monopoly, Digital Economy Innovation and Industrial Structure Upgrading. *China Indus. Eco.* 2023, 61–79, doi:10.19581/j.cnki.ciejournal.2023.10.003.
24. Picard, P.M.; Zeng, D.Z. Agricultural Sector and Industrial Agglomeration. *J. Dev. Econ.* **2005**, *77*, 75–106, doi:10.1016/j.jdeveco.2004.03.002.
25. Mao, N.; Sun, W.; Yang, Y. Transportation Infrastructure Construction and Enterprise Digital Transformation—An Empirical Study on China's High-speed Railway. *J. Quan. Tech. Econ.* **2022**, *39*, 47–67, doi:10.13653/j.cnki.jqte.20220920.003.
26. Deng, Y.; Sun, H. The Effect of Industrial Agglomeration on Economic Resilience and Its Mechanism. *Soft Sci.* **2022**, *36*, 48-54+61, doi:10.13956/j.ss.1001-8409.2022.03.07.
27. He, Z.; Li, Y.; Wu, Y. Industrial Collaborative Agglomeration, Technological Innovation and Manufacturing Industrial Chain Resilience. *Stu. Sci. Sci.* **2023**, 1–16, doi:10.16192/j.cnki.1003-2053.20230515.001.
28. Chen, J.; Chen, G.; Huang, J. A Study of Productive Service Industry Agglomeration and Its Influencing Factors Under the Perspective of New Economic Geography:Empirical Evidence from 222 Cities in China. *J. Manag. Wor.* **2009**, 83–95, doi:10.19744/j.cnki.11-1235/f.2009.04.010.
29. Zhao, T.; Zhang, Z.; Liang, S. Digital Economy, Entrepreneurship, and High-Quality Economic Development: Empirical Evidence from Urban China. *J. Manag. Wor.* **2020**, *36*, 65–76, doi:10.19744/j.cnki.11-1235/f.2020.0154.
30. Zhao, W.; Xu, X. Effect and Mechanism of Digital Economy on the Resilience of Agricultural Economy. *J. Sou. China Agric. Uni. (Soc. Sci. Edi.)* **2023**, *22*, 87–96.
31. Jiang, T. Mediating Effects and Moderating Effects in Causal Inference. *China Indu. Econ.* **2022**, 100–120, doi:10.19581/j.cnki.ciejournal.2022.05.005.

32. Zurek, M.; Ingram, J.; Bellamy, A.S.; Goold, C.; Lyon, C.; Alexander, P.; Barnes, A.; Bebber, D.P.; Breeze, T.D.; Bruce, A.; et al. Food System Resilience: Concepts, Issues, and Challenges. *Annu. Rev. Environ. Resour.* **2022**, *47*, 511–534, doi:10.1146/annurev-environ-112320-050744.
33. Zhu, X.; Wei, G. Discussion on the Good Standard of Dimensionless Method in Entropy method. *Stat. Dec.* **2015**, 12–15, doi:10.13546/j.cnki.tjyjc.2015.02.003.
34. Wang, J.; Zhu, J.; Luo, Q. Development Level and Evolution Measurement of China's Digital Economy. *Resea. Quant. Tech. Econ.* **2021**, *38*, 26–42, doi:10.13653/j.cnki.jqte.2021.07.002.
35. Wan, X.; Luo, Y. Measurement of the Development Level of Digital Economy and Its Impact Effect on Total Factor Productivity. *Reform* **2022**, 101–118.
36. Wang, D.; Peng, Z.; Li, L. Measuring and Evaluating the Integrated Development Level of Digital Economy and Agriculture in China. *Chinese Rur. Econ.* **2023**, 48–71, doi:10.20077/j.cnki.11-1262/f.2023.06.004.
37. Han, H.; Yang, D. Spatial Spillover Effects of Agricultural Industrial Agglomeration on the Growth of Agricultural Green Total Factor Productivity. *J. Ari. Land Resou. Envir.* **2023**, *37*, 29–37, doi:10.13448/j.cnki.jalre.2023.134.
38. Yang, X.; Qiao, C. Spatial Spillover Effects of Agricultural Industry Agglomeration on Agricultural Carbon Productivity: Based on the Regulatory Role of Fiscal Decentralization. *China Pop.,Res. Enviro.* **2023**, *33*, 92–101.
39. Cui, N.; Liu, Z.; Dong, J. Internal Logic and Long-term Mechanism Construction of Smart Agriculture for Reducing Losses of Grain Production. *Iss. Agric. Eco.* **2023,** 116–128, doi:10.13246/j.cnki.iae.2023.10.006.
40. Rural Revitalization Research Group of Wuhan University. Promote Rural Revitalization in All Respects and Ensure Food Security. *Fina. & Tra. Eco.* **2022**, *43*, 5–18, doi:10.19795/j.cnki.cn11-1166/f.20220505.009.
41. Zhou, H.; Liu, Y.; Guo, J. Analysis On FDI in Land of Developing Countries and Its Implications. *China Sof. Scie.* **2011**, 41–54.
42. Wang, X. To Ensure Food Security, We Need to Seize the Commanding Heights of Global Seed Industry Technology. *People's Tribune* **2020,** 64–65.
43. Guo, X.; He, Q.; Zhang, D. Application of Subjective and Objective Methods in Weights Determination of Synthetic Evaluation of Science and Technology. *Scie. Tech. Mana. Res.* **2012,** *32,* 64-67+71.
44. Huang, Q.; Yu, Y.; Zhang, S. Internet Development and Productivity Growth in Manufacturing Industry:Internal Mechanism and China Experiences. *China Indu. Econ.* 2019, 5–23, doi:10.19581/j.cnki.ciejournal.2019.08.001.
45. Yi, E.; Wang, J.; Zhu, J. Digital Economy, Resource Allocation Efficiency and High- Quality Development of Agriculture. *Mod. Fin. Econ.-J.Tianjin Univ. Fina. Eco.* **2023,** 20–37, doi:10.19559/j.cnki.12-1387.2023.12.002.
46. Nunn, N.; Qian, N. US Food Aid and Civil Conflict. *Am. Econ. Rev.* **2014**, *104*, 1630–1666, doi:10.1257/aer.104.6.1630.
