# Supplementary material for: The impact of the digital economy on food system resilience: Evidence from China
Source: PLoS One. 2024 Oct 24;19(10):e0311689. doi: 10.1371/journal.pone.0311689 (PMC11500886; doi:10.1371/journal.pone.0311689)
Supplement: Attachment — Submitted filename: Response to Reviewers.docx [file pone.0311689.s004.docx]

Response to Reviewers

**Reviewer #1:**

1. **discuss the novelty of the study so we understand why worthy reading the manuscript**

**Response：**Thank you very much for your careful review and valuable comments on our article. We have highlighted the innovation of this article in the summary, so that readers can better understand the main contributions of this article.

Despite the widespread influence of the burgeoning digital economy on agricultural productivity in recent years, China's food system confronts numerous challenges. Notably, research exploring the digital economy's impact on food system resilience remains scarce, and the pivotal role of industrial agglomeration in this context remains unclear. Therefore, this article is based on……

2. **Justify why you chose only one bank -- potential of introducing bias into the study**

**Response：**Thank you very much for your careful review and valuable comments on our article. We are very sorry that we do not quite understand the meaning of this sentence. If you are talking about our research objects, whether it is the digital economy or food system resilience, they are all constructed by the indicator system, so they are representative to a certain extent.

3. **Discuss your choice of smaple (** **why concentrate only on three areas)**

**Response：**Thank you very much for your careful review and valuable comments on our article. The sample data in this article mainly come from China Rural Statistical Yearbook, China Statistical Yearbook, EPS database, and statistical yearbooks and statistical bulletins of provinces and cities in corresponding years. The research object is 30 provinces in China (except Tibet, Hong Kong, Macao and Taiwan) from 2011 to 2021. It's not only on three areas.

4. **Its important to show the actual structural equation that was estimated rather than mentioning the variables**

**Response：**Thank you very much for your careful review and valuable comments on our article. We have revised our introduction to equation.

5. **Kindly show the marginal effects and discuss their implications for the study.**

**Response：**Thank you very much for your careful review and valuable comments on our article. We revised and explained.

6. **Is Table 2 logit regression model or what?**

**Response：**Thank you very much for your careful review and valuable comments on our article. Table 2 is the index system of the digital economy.

7. **Explain clearly the method that was used to generate the IV system**

**Response：**Thank you very much for your careful review and valuable comments on our article. We explain in detail the reasons and practices of IV so that readers can understand them more clearly.

In general, regions that exhibit robust resilience within their food systems generally possess more advanced digital infrastructure, suggesting that digital economy development confers a "first-mover advantage." This implies a potential bidirectional causality between the digital economy and food system resilience, making it challenging to conclusively ascertain whether the latter's enhancement stems solely from the former's progression. Consequently, this study employs the panel instrumental variable approach to address the issue of endogeneity. Therefore, in this study, based on the experience of Huang Qunhui et al. (2019) [44] and the availability of data, the number of landline phones at the end of 1990 (in 10,000 households) in each region is selected as the instrumental variable, and a regression test is conducted using the two-stage least squares method.

The instrumental variable selection in this study is grounded on several pivotal considerations: Firstly, the ubiquitous adoption of Internet technology forms the corner-stone for digital economy growth. Tracing back, the advent of Internet technology can be attributed to the widespread use of fixed-line telephones. Regions boasting high penetration rates of fixed-line telephones also exhibit a relatively advanced digital economy, suggesting a strong correlation between digital economic progress and fixed-line penetration. Specifically, the digital economy's core momentum stems from Internet advancements, which can be viewed as an extension of fixed-line telephony's proliferation. This implies that the prevalence of fixed-line telephones constitutes a vital aspect of digital economy infrastructure, fulfilling the relevance criterion. Conversely, in the era of rapid communication technology evolution, traditional landlines contribute minimally to the resilience of contemporary food systems, thereby meeting the exclusivity requirement [45]. It is worth noting that the number of landline telephones at the end of 1990 comprises cross-sectional data, while the sample in this paper comprises balanced panel data. Therefore, based on Nunn and Qian (2014) [46], the cross-multiplier term between the number of Internet access instances in the previous year and the number of landline telephones at the end of 1990 is constructed as an instrumental variable (IV) for the digital economy index of the current period. The estimation results are presented in Table 6. The results in column (1) indicate that the instrumental variable has a significant positive effect on the digital economy, and the results in column (2) show that after applying the two-stage least squares method to mitigate the endogeneity problem, the digital economy still passes the positive significance test on the resilience of the food system and is significant at the 5% level, which is basically the same as the results of the benchmark regression. In addition, the results of the weak instrumental variable test show that the F-statistic is 241.435, which is significantly greater than the critical value of 10; therefore, there is no weak instrumental variable problem. Accordingly, this indicates that the development of the digital economy determines the improvement of the resilience of the food system, which verifies hypothesis H1.

**Reviewer #2:**

**1- Line 31-33**

**It is preferable to write an actual number instead of an expected one because we are in 2024.**

**Response：**Thank you very much for your careful review and valuable comments on our article. According to the latest China Bureau of Statistics data, China's total national grain output in 2023 was 695.41 million tons, exceeding 690 million tones. So, we have replaced the original sentence with the following:

“Since 2015, China has achieved sustained growth in total grain output, reaching a new level of 650 million tons. By 2023, it was reached over 690 million tons, despite possessing less than 10% of the world’s arable land to feed nearly 20% of the world’s population.”

**2-Add a simple definition for each of:**

a) **Line 87 "siphon effect"**
b) **line 168 "MAR externality"**
c) **Line 260 "****entropy method"**
d) **Line 386 "CRITIC"**

**Response：**Thank you very much for your careful review and valuable comments on our article. We have made the following modifications in the article:

a) The digital economy exacerbates the “siphon effect,” where a city's competitive advantages, including digital technologies and information flow, draw resources like businesses, technologies, and knowledge from surrounding areas, resulting in their concentration within the city and leading to the formation of a network system to jointly protect against risks.
b) However, theories such as Marshall-Arrow-Romer (MAR) externality, Porter externality, and Jacob externality highlight the positive impact of industrial agglomeration on economic resilience.
c) To avoid measurement bias caused by inconsistent indicators, this paper uses the “en-tropy method,” a statistical technique that standardizes indicators by assigning weights based on the information content or variability of each indicator, to standardize indicators and measure food system resilience objectively by assigning weights to each indicator.
d) The entropy value method was used in the previous section to measure the composite index, and the CRITIC and entropy value methods are both objective weighting methods. The "CRITIC" method, in simple terms, is a multi-criteria decision analysis tool that as-signs weights to indicators based on both the contrast strength (how well the indicators discriminate among evaluation units) and the conflict among criteria (how much correlation exists between indicators), thus providing an objective evaluation framework. Therefore, the CRITIC method avoids the problem of weighting when dealing with the comprehensive evaluation of multiple indicators, but it also takes into account the effect of correlations on the weight of indicators and eliminates the effect of correlation indicators on the weight during weighting [43].

**3- Line 93-101**

**The author mentioned that some studies had shortcomings in food system resilience.**

**It is preferable to refer to these studies in this paragraph.**

**Response：**Thank you very much for your careful review and valuable comments on our article. We have made the following changes in the article to highlight the shortcomings more prominently.

However, there are still some shortcomings that need to be addressed: Notably, while China has placed significant emphasis on the development of the digital economy and achieved notable successes, the supporting and enabling role of the digital economy in promoting high-quality industrial development has become increasingly evident. However, research examining the resilience of the digital economy in relation to the food system remains scarce. Additionally, there is a paucity of studies that have delved into the intrinsic linkages among the three domains, specifically including the dimension of industrial agglomeration, thereby neglecting to comprehensively explore their inter-connectedness.

**4-Equations 2 and 3:**

**Use the idea of endogeneity…. What is the model’s name?**

**It is better to add it here, not in line 404-407.**

**Response：**Thank you very much for your careful review and valuable comments on our article. The Equations 2 and 3 model’s name is “Mediation effects model”

**5- Table 1**

**It would be nice to explain some signs that may be illogical, such as fertilizer use intensity.**

**Response：**Thank you very much for your careful review and valuable comments on our article. In constructing the index system for evaluating the level of food system resilience, we divided it into three secondary indicators, namely, resistance, adaptability and changeability, according to their connotations, and selected six, seven and four tertiary indicators, respectively, for the following reasons:

1. **Cultivated land per capita:** This metric quantifies the availability of land resources for food production, forming the foundation of agricultural productivity. A larger per capita cultivated land area theoretically indicates a greater potential for enhanced food production, thereby contributing to food system resilience.
2. **Labor Force Level:** Labor is a pivotal element in food production, encompassing the quantity, quality (e.g., skill proficiency, educational attainment), and efficiency of the workforce. A skilled and efficient labor force fosters improved food production efficiency and quality, reinforcing the resilience of the food system.
3. **Level of fixed asset investment:** Comprising investments in farmland infrastructure and agricultural machinery, this metric underpins the development of modern agriculture. Heightened fixed asset investments bolster agricultural production capabilities, thereby strengthening the resilience of the food system.
4. **Grain production per capita:** A direct measure of production efficiency, this metric assesses the food system's output capacity on a per capita basis, contributing to its overall resilience.
5. **Food production price index:** Reflecting variations in food market prices, this index significantly impacts farmers' incomes and market stability, serving as a crucial economic indicator for food system resilience.
6. **Grain Production Level:** An aggregate metric, it represents the overall scale and supply capacity of food production within a country or region, a key aspect of food system resilience.
7. **Intensity of pesticide use, Fertilizer use intensity and Intensity of agricultural film use:** Though potentially controversial, constitute pivotal aspects in evaluating the sustainability of agricultural production. Excessive use of pesticides and fertilizers can lead to soil contamination, water pollution, and ecological destruction, thereby impacting long-term food production capabilities. Moreover, the issue of agricultural film residue has garnered increasing attention. Consequently, monitoring these indicators is instrumental in guiding agriculture towards a more environmentally friendly and sustainable development path.
8. **Degree of disaster:** The extent of disaster damage reflects the resilience of the food production system against natural hazards. A robust food system ought to possess robust disaster resistance, minimizing the impact of natural disasters on food production.
9. **Replanting index:** Reflecting land use optimization and agricultural planning, this index represents an effective strategy for enhancing food yields, which is essential for maintaining and improving food system resilience.
10. **Agricultural value add index and Per capita rural electricity consumption:** Respectively, these metrics indicate agricultural economic growth and the extent of rural electrification, both of which are key indicators of agricultural modernization and contribute to the resilience of the food system.
11. **Degree of mechanization:** Widespread mechanization substantially elevates agricultural productivity while mitigating labor intensity, a testament to agricultural modernity and a crucial factor in enhancing food system resilience.
12. **Level of agricultural development:** A comprehensive evaluation of the overall agricultural production status, encompassing technological advancements, organizational structures, and industrialization levels, this index provides a holistic view of the food system resilience.
13. **Level of Expenditure on Agricultural Research and Level of agricultural technicians:** These indicators underscore the importance of technological investments and human capital in fortifying the resilience of the food system. Scientific innovations and technological advancements serve as pivotal drivers of sustainable agricultural development and are essential for maintaining and enhancing food system resilience.

But, after thorough consideration, we have opted to forgo a detailed explanation of the 17 tertiary indicators in the paper. Our primary justifications are as follows:

- Commonality of Indicator Selection: Given that all 17 tertiary indicators were meticulously chosen based on existing research, their selection process is inherently conventional and widely accepted within the academic community.
- Minimizing Subjectivity and Text Length: Providing explanations solely for select indicators would inevitably introduce a degree of subjectivity and significantly lengthen the text. To maintain conciseness and objectivity, we have decided to refrain from such explanations.

By adhering to these principles, we aim to present a clear and focused paper that remains academically rigorous while maintaining readability and accessibility.

**6- The title was repeated in rows 395 and 434.**

**Response：**Thank you very much for your careful review and valuable comments on our article. We have changed 4.5 to the Mechanism of Action Test.

**7- The recommendations were long, so it would be nice to shorten them.**

**Response：**Thank you very much for your careful review and valuable comments on our article. Under the condition of retaining the core meaning and main points of the original text, we revise and reduce the “**recommendations”** part to the following contents:

Firstly, we propose enhancing infrastructure construction and fostering digital village development. To bridge the gap in rural digital infrastructure, prioritize support for the digital transformation of mobile communication base stations and terminals. Expedite the deployment of efficient, high-speed mobile networks, particularly 5G coverage in remote rural areas. Further explore 5G applications to refine data perception, collection, filtering, and transmission, establishing a digital information highway for the grain industry and solidifying the digital economy's foundation. Additionally, elevate farmers' digital literacy and IT application skills, advocating the principle of "integrating information technology with agricultural machinery and agronomy for smart agriculture advancement." Strengthen ideologies by enhancing awareness of smart agriculture and practical food production skills, conducting digital agriculture training, fostering a mindset for food quality development, cultivating digital agricultural literacy, and accelerating new vocational digital agriculture education.

Secondly, reinforce industrial agglomeration to boost food quality and efficiency. Industrial agglomeration is vital for fostering resilience in the digital economy-driven food system. Improve market-oriented development to attract upstream and downstream clusters. Foster technological innovation through large-scale, region-specific projects, and increase R&D investments and talent development to encourage agricultural professionals' grassroots engagement. Create a favorable, market-driven, and legal international business environment. Avoid blindly pursuing foreign investments, focusing instead on transitioning from agricultural quantity to quality. Multi-faceted efforts will elevate industrial development's quality and efficiency, gradually enhancing food system resilience.

Lastly, promote regionally integrated development to secure food safety. Recognizing the interconnectedness of regional food system resilience, tailor food development strategies to local conditions. Emphasize enhancing specialty food quality, building local brands, and innovating business models. Strengthen inter-city food industry cooperation, unifying the national market, and transcending local barriers. Facilitate effective "hardware" and "software" integration across regions, promoting platform development, industrial integration, public services, and talent exchanges within administrative zones.

**8- Write the references in the research, as specified in the journal.**

**Response：**Thank you very much for your careful review and valuable comments on our article. We have revised the format of the reference according to the requirements of the journal.

**Reviewer #3:**

**1- Line 31: At the end of this line, the result of a prediction in 2023 is mentioned. Now the year 2023 is over. Has it reached this number?!**

**Response：**Thank you very much for your careful review and valuable comments on our article. According to the latest China Bureau of Statistics data, China's total national grain output in 2023 was 695.41 million tons, exceeding 690 million tones. So, we have replaced the original sentence with the following:

“Since 2015, China has achieved sustained growth in total grain output, reaching a new level of 650 million tons. By 2023, it was reached over 690 million tons, despite possessing less than 10% of the world’s arable land to feed nearly 20% of the world’s population.”

**2- Lines 114 and 115: A sentence has been repeated.**

**Response：**Thank you very much for your careful review and valuable comments on our article. We have deleted one of the two sentences.

**3- Line 167: What word does MAR stand for? Introduce it for the first time!**

**Response：**Thank you very much for your careful review and valuable comments on our article. We have revised the original sentence to read: “However, theories such as Marshall-Arrow-Romer (MAR) externality, Porter externality, and Jacob externality highlight the positive impact of industrial agglomeration on economic resilience. "So that the readers can understand the meaning of MAR.

**4-Line 195: The word Impact should be deleted.**

**Response：**Thank you very much for your careful review and valuable comments on our article. We deleted the word impact.

**5-Line 224: these are not correlation coefficients but they are regression coefficients that are completely different from correlation coefficients.**

**Response：**Thank you very much for your careful review and valuable comments on our article. We have revised the description of all equations.

**6-Line 231: In equation 2, Controlit cannot be the same variables of equation 1!!!**

**Response：**Thank you very much for your careful review and valuable comments on our article. We have modified the description of the control variable.

**7-Line 232: In equation 3, variable Digit replaced by the equation 2? Why is it included in the equation 3 again?**

**Response：**Thank you very much for your careful review and valuable comments on our article. There is an error in equation 2 of the current article, we have revised it to the following equation. Equation 2 explores the effect of Dig on Agg, while equation 3 explores the combined effect of Dig and Agg and control variables on FSR.

$$\text{Agg}_{\text{it}}=\alpha_{1}+aDig_{\mathrm{it}}+\beta_{1}\sum\text{Control}_{\text{it}}\text{+}\text{μ}_{\text{i}}\text{+}{\pi_{t}+\text{ε}}_{\text{it}}$$

**8- Line 234: Correlation coefficient should be regression coefficient.**

**Response：**Thank you very much for your careful review and valuable comments on our article. We have revised the description of all equations.

**9- Line 255: Is the variable food system resilience (FSR) an explanatory variable? Usually, in statistics or econometrics, the variable on the left side of the equation is called the dependent variable, and the variables on the right side of the equation are called explanatory variables.**

**Response：**Thank you very much for your careful review and valuable comments on our article. this is indeed a typo on our part and we have corrected it to the dependent variable.

**10- Line 257: the word of system after index should be deleted.**

**Response：**Thank you very much for your careful review and valuable comments on our article. We have deleted the word of system after index.

**11- Line 263, Table 1: How is the variable value added converted into percentage?!**

**Response：**Thank you very much for your careful review and valuable comments on our article. We have revised the wording of the calculation method and unit for the value added. For the reader to better understand.

| Food production price index | Food production prices of the current/previous year (previous year=100) |
| --- | --- |
| Agricultural value add index | Agricultural value added in the current/previous year (previous year=100) |

**12- Line 277: Correct the equation number, (4) should be (6).**

**Response：**Thank you very much for your careful review and valuable comments on our article. We have made changes.

**13-Line 278: Equation (8) should be Equation (6).**

**Response：**Thank you very much for your careful review and valuable comments on our article. We have made changes.

**14-Line 283: Control variable should be control variables!!**

**Response：**Thank you very much for your careful review and valuable comments on our article. We have made changes.

**15-Line 364: The tittle of table 4 should be regression results not Descriptive statistics of variables.**

**Response：**Thank you very much for your careful review and valuable comments on our article. We have changed the tittle of Table 4 to Baseline regression result.

**16- Considering that the data used are panel, why haven't you used the Limer test?**

**Response：**Thank you very much for your careful review and valuable comments on our article. We conducted the LM test and expounded it as follows in the paper:

Given the panel data nature of the variables, we subsequently assessed the suitability of mixed regression, random effects, and fixed effects models. The F-test decisively favored fixed effects over mixed regression, with Prob > F = 0.0000. Furthermore, the Hausman test indicated a preference for the fixed effects model over the random effects model, as evidenced by Prob > chi2 = 0.0299. Consequently, this study adopts the bidirectional fixed effects model for regression analysis.

**17- Why is the unit root test not done on the variables?**

**Response：**Thank you very much for your careful review and valuable comments on our article. We have carried out a unit root test on the variables and attached the test results here, as well as the relevant descriptions in the paper. However, the results are not shown in detail in this paper. The main consideration is that neither the unit root test nor the multicollinearity test is the focus of this paper. Therefore, on the basis of ensuring the validity of the data, we only give necessary descriptions in this paper. The relevant descriptions in the article are as follows:

- To circumvent the peril of "pseudo-regression" arising from non-stationary data, an IPS unit root test was administered, ensuring that all selected variables exhibited stationarity at a significance level exceeding 10%.

| **Variable** | **IPS Z-value** |
| --- | --- |
| FSR | -4.4623 |
| Dig | -2.3810 |
| Agg | -2.7100 |
| Inf | -3.5946 |
| Emp | -2.1697 |
| For | -2.8710 |
| Tec | -1.8296 |
| Ind | -1.4340 |
| Open | -1.9432 |

**18- Line 456: is this last column confidence interval? where is interval?!! they are points!!**

**Response：**Thank you very much for your careful review and valuable comments on our article. We have supplemented the missing data in Table 8.

| **Influence type** | **Coefficient** | **SD** | **P-value** | **95% confidence interval** | |
| --- | --- | --- | --- | --- | --- |
| Indirect effect | 0.068 | 0.019 | 0.000 | 0.036 | 0.115 |
| Direct effect | 0.232 | 0.036 | 0.003 | 0.174 | 0.316 |

**19- Line 466: Table 9, Why are the fluctuations of Moran's index so high in some cases? For example, in the years 2017, 2018 and 2019, this index for the FSR variable first decreased sharply and then increased? Has something special happened in the studied areas in these years?! If nothing special happened, isn't the validity of the data questioned?**

**Response：**Thank you very much for your careful review and valuable comments on our article. We used Stata software to re-check the Moran's index of FSR and found that there was no difference with the existing results, indicating that there was no filling error in the data results in the table. In order to prove the validity of the data, we presented the display results of relevant years in the form of screenshots. However, the reasons for the large variations in values still need to be further explored. The possible reasons are:

(2017-2018) During this period, China's summer grain, led by the six major grain producing areas, suffered serious natural disasters and significantly reduced production. These disasters impacted food production to varying degrees, leading to differences in the resilience of food systems across regions. The unpredictability and regionality of natural disasters have severely impaired food production and reduced resilience in some regions, thus affecting overall spatial linkages.

(2018-2019) After the impact of natural disasters in the previous year, regions may have stepped up their efforts in post-disaster recovery and reconstruction. The government and all sectors of society will work together to help the affected areas restore food production and improve infrastructure through financial, technical and material support. Such post-disaster recovery and reconstruction efforts help improve the resilience of food systems in affected areas and promote synergistic development across regions.

**20-Line 476: Why are not the values of LM, LR and Hausman tests reported in the table?**

**Response：**Thank you very much for your careful review and valuable comments on our article. We have added a new table to report the values of LM, LR and Hausman tests.

**Table 10.** Test results of spatial econometric model.

| **Model** | **LM** | **Robust LM** | **Wald** | **LR** |
| --- | --- | --- | --- | --- |
| SLM | 5.270* | 2.992* | 7.542** | 14.687** |
| SEM | 33.397*** | 32.118*** | 8.100** | 16.212*** |
