# Supplementary material for: The impact of the digital economy on food system resilience: Evidence from China
Source: PLoS One. 2024 Oct 24;19(10):e0311689. doi: 10.1371/journal.pone.0311689 (PMC11500886; doi:10.1371/journal.pone.0311689)
Supplement: Peer review history

# Peer review: Decision Letter 0

**DOI:** 10.1371/journal.pone.0311689.r001

**Authors:** Eda Ustaoglu

12 Aug 2024

PONE-D-24-27750The Impact of the Digital Economy on Food System Resilience: Evidence from ChinaPLOS ONE

Dear Dr. Wang,

Thank you for submitting your manuscript to PLOS ONE. After careful consideration, we feel that it has merit but does not fully meet PLOS ONE’s publication criteria as it currently stands. Therefore, we invite you to submit a revised version of the manuscript that addresses the points raised during the review process.The reviewers point to minor issues that need to be revised according to their suggestions and you can view these comments in the reviewers' reports.

Please submit your revised manuscript by Sep 26 2024 11:59PM. If you will need more time than this to complete your revisions, please reply to this message or contact the journal office at plosone@plos.org. When you're ready to submit your revision, log on to https://www.editorialmanager.com/pone/ and select the 'Submissions Needing Revision' folder to locate your manuscript file.

Please include the following items when submitting your revised manuscript:

- A rebuttal letter that responds to each point raised by the academic editor and reviewer(s). You should upload this letter as a separate file labeled 'Response to Reviewers'.
- A marked-up copy of your manuscript that highlights changes made to the original version. You should upload this as a separate file labeled 'Revised Manuscript with Track Changes'.
- An unmarked version of your revised paper without tracked changes. You should upload this as a separate file labeled 'Manuscript'.

If you would like to make changes to your financial disclosure, please include your updated statement in your cover letter. Guidelines for resubmitting your figure files are available below the reviewer comments at the end of this letter.

If applicable, we recommend that you deposit your laboratory protocols in protocols.io to enhance the reproducibility of your results. Protocols.io assigns your protocol its own identifier (DOI) so that it can be cited independently in the future. For instructions see: https://journals.plos.org/plosone/s/submission-guidelines#loc-laboratory-protocols. Additionally, PLOS ONE offers an option for publishing peer-reviewed Lab Protocol articles, which describe protocols hosted on protocols.io. Read more information on sharing protocols at https://plos.org/protocols?utm_medium=editorial-email&utm_source=authorletters&utm_campaign=protocols.

We look forward to receiving your revised manuscript.

Kind regards,

Eda Ustaoglu, PhD

Academic Editor

PLOS ONE

Journal Requirements:

When submitting your revision, we need you to address these additional requirements.

1. Please ensure that your manuscript meets PLOS ONE's style requirements, including those for file naming. The PLOS ONE style templates can be found at 

https://journals.plos.org/plosone/s/file?id=wjVg/PLOSOne_formatting_sample_main_body.pdf and 

https://journals.plos.org/plosone/s/file?id=ba62/PLOSOne_formatting_sample_title_authors_affiliations.pdf

2. Please note that funding information should not appear in any section or other areas of your manuscript. We will only publish funding information present in the Funding Statement section of the online submission form. Please remove any funding-related text from the manuscript.

3. Thank you for stating the following financial disclosure: 

   "Funding: This research was funded by the National Natural Science Foundation of China (grant no. 71563016), Key Research Base Project of Humanities and Social Sciences in colleges and uni-versities of Jiangxi Province (JD21080), the Jiangxi Postgraduate Innovation Special Fund Project (YC2022-s415), and the Jiangxi Agricultural University School of Economics and Management 2022 Postgraduate Innovation Special Fund Project (grant no. JG2022008)."

Please state what role the funders took in the study.  If the funders had no role, please state: "The funders had no role in study design, data collection and analysis, decision to publish, or preparation of the manuscript." 

If this statement is not correct you must amend it as needed. 

Please include this amended Role of Funder statement in your cover letter; we will change the online submission form on your behalf.

4. In the online submission form, you indicated that "If you need any data, you can contact any of the authors to request it." 

All PLOS journals now require all data underlying the findings described in their manuscript to be freely available to other researchers, either 1. In a public repository, 2. Within the manuscript itself, or 3. Uploaded as supplementary information.

This policy applies to all data except where public deposition would breach compliance with the protocol approved by your research ethics board. If your data cannot be made publicly available for ethical or legal reasons (e.g., public availability would compromise patient privacy), please explain your reasons on resubmission and your exemption request will be escalated for approval. 

5. Please review your reference list to ensure that it is complete and correct. If you have cited papers that have been retracted, please include the rationale for doing so in the manuscript text, or remove these references and replace them with relevant current references. Any changes to the reference list should be mentioned in the rebuttal letter that accompanies your revised manuscript. If you need to cite a retracted article, indicate the article’s retracted status in the References list and also include a citation and full reference for the retraction notice.

[Note: HTML markup is below. Please do not edit.]

Reviewers' comments:

Reviewer's Responses to Questions

**Comments to the Author**

1. Is the manuscript technically sound, and do the data support the conclusions?

The manuscript must describe a technically sound piece of scientific research with data that supports the conclusions. Experiments must have been conducted rigorously, with appropriate controls, replication, and sample sizes. The conclusions must be drawn appropriately based on the data presented. 

Reviewer #1: Yes

Reviewer #2: Yes

Reviewer #3: Yes

**********

2. Has the statistical analysis been performed appropriately and rigorously? 

Reviewer #1: Yes

Reviewer #2: Yes

Reviewer #3: Yes

**********

3. Have the authors made all data underlying the findings in their manuscript fully available?

The PLOS Data policy requires authors to make all data underlying the findings described in their manuscript fully available without restriction, with rare exception (please refer to the Data Availability Statement in the manuscript PDF file). The data should be provided as part of the manuscript or its supporting information, or deposited to a public repository. For example, in addition to summary statistics, the data points behind means, medians and variance measures should be available. If there are restrictions on publicly sharing data—e.g. participant privacy or use of data from a third party—those must be specified.

Reviewer #1: No

Reviewer #2: Yes

Reviewer #3: Yes

**********

4. Is the manuscript presented in an intelligible fashion and written in standard English?

PLOS ONE does not copyedit accepted manuscripts, so the language in submitted articles must be clear, correct, and unambiguous. Any typographical or grammatical errors should be corrected at revision, so please note any specific errors here.

Reviewer #1: No

Reviewer #2: Yes

Reviewer #3: Yes

**********

5. Review Comments to the Author

Please use the space provided to explain your answers to the questions above. You may also include additional comments for the author, including concerns about dual publication, research ethics, or publication ethics. (Please upload your review as an attachment if it exceeds 20,000 characters)

Reviewer #1: 1. discuss the novelty of the study so we understand why worthy readin the manuscript

2. Justify why you chose only one bank -- potential of introducing bias into the study

3. Discuss your choice of smaple ( why concentrate only on three areas)

4. Explain clearly the choice of instruments and how they realte to the differnt variables of interest

5. Its important to show the actual structural equation that was estimated rather than mentioning the variables

6. Kindly show the marginal effects and discuss their implications for the study

7. Is Table 2 logit regression model or what?

8. Explain clearly the method that was used to generate the IV system

Reviewer #2: The author did a good job of studying the empowerment of food system resilience using the digital economy to achieve food security. The authors used panel data from 2011 to 2021. The paper used appropriate models to achieve the study objective. Therefore, I recommend that the research be accepted for publication.

However, there may be additions that may give the research more scientific strength:

1- Line 31-33

It is preferable to write an actual number instead of an expected one because we are in 2024.

2-Add a simple definition for each of:

Line 87 "siphon effect"

line 168 "MAR externality"

Line 260 "entropy method"

Line 386 "CRITIC"

3- Line 93-101

The author mentioned that some studies had shortcomings in food system resilience.

It is preferable to refer to these studies in this paragraph.

4-Equations 2 and 3:

Use the idea of endogeneity…. What is the model’s name?

It is better to add it here, not in line 404-407.

4- Table 1

It would be nice to explain some signs that may be illogical, such as fertilizer use intensity.

5- The title was repeated in rows 395 and 434.

6- The recommendations were long, so it would be nice to shorten them.

7- Write the references in the research, as specified in the journal.

Reviewer #3: This manuscript is admirable for its subject matter, research methodology and data used. The impact of the digital economy on the important issue of the food system resilience, which is somehow related to food security, has been evaluated. The author (s) has (have) been able to use the requested statistical methods well and achieve the objectives of the study. At the same time, it seems that paying attention to the following will help to improve the study:

1- Line 31: At the end of this line, the result of a prediction in 2023 is mentioned. Now the year 2023 is over. Has it reached this number?!

2- Lines 114 and 115: A sentence has been repeated.

3- Line 167: What word does MAR stand for? Introduce it for the first time!

4-Line 195: The word Impact should be deleted.

5-Line 224: these are not correlation coefficients but they are regression coefficients that are completely different from correlation coefficients.

6-Line 231: In equation 2, Controlit cannot be the same variables of equation 1!!!

7-Line 232: In equation 3, variable Digit replaced by the equation 2? Why is it included in the equation 3 again?

8- Line 234: Correlation coefficient should be regression coefficient.

9- Line 255: Is the variable food system resilience (FSR) an explanatory variable? Usually, in statistics or econometrics, the variable on the left side of the equation is called the dependent variable, and the variables on the right side of the equation are called explanatory variables.

10- Line 257: the word of system after index should be deleted.

11- Line 263, Table 1: How is the variable value added converted into percentage?!

12- Line 277: Correct the equation number, (4) should be (6).

13-Line 278: Equation (8) should be Equation (6).

14-Line 283: Control variable should be control variables!!

15-Line 364: The tittle of table 4 should be regression results not Descriptive statistics of variables.

16- Considering that the data used are panel, why haven't you used the Limer test?

17- Why is the unit root test not done on the variables?

18- Line 456: is this last column confidence interval? where is interval?!! they are points!!

19- Line 466: Table 9, Why are the fluctuations of Moran's index so high in some cases? For example, in the years 2017, 2018 and 2019, this index for the FSR variable first decreased sharply and then increased? Has something special happened in the studied areas in these years?! If nothing special happened, isn't the validity of the data questioned?

20-Line 476: Why are not the values of LM, LR and Hausman tests ​​reported in the table?

**********

6. PLOS authors have the option to publish the peer review history of their article (what does this mean?). If published, this will include your full peer review and any attached files.

If you choose “no”, your identity will remain anonymous but your review may still be made public.

**Do you want your identity to be public for this peer review?** For information about this choice, including consent withdrawal, please see our Privacy Policy.

Reviewer #1: **Yes: **Sanderson Abel

Reviewer #2: No

Reviewer #3: No

**********

[NOTE: If reviewer comments were submitted as an attachment file, they will be attached to this email and accessible via the submission site. Please log into your account, locate the manuscript record, and check for the action link "View Attachments". If this link does not appear, there are no attachment files.]

While revising your submission, please upload your figure files to the Preflight Analysis and Conversion Engine (PACE) digital diagnostic tool, https://pacev2.apexcovantage.com/. PACE helps ensure that figures meet PLOS requirements. To use PACE, you must first register as a user. Registration is free. Then, login and navigate to the UPLOAD tab, where you will find detailed instructions on how to use the tool. If you encounter any issues or have any questions when using PACE, please email PLOS at figures@plos.org. Please note that Supporting Information files do not need this step.

Attachment Submitted filename: PONE-D-24-27750.pdf

# Author comment: Author response to Decision Letter 0

**DOI:** 10.1371/journal.pone.0311689.r002

20 Aug 2024

Due to excessive content to be filled in, including Figure and tables, please refer to the Response to Reviewers file for details.

Attachment Submitted filename: Response to Reviewers.docx

# Peer review: Decision Letter 1

**DOI:** 10.1371/journal.pone.0311689.r003

**Authors:** Eda Ustaoglu

24 Sep 2024

The impact of the digital economy on food system resilience: Evidence from China

PONE-D-24-27750R1

Dear Dr. Wang,

We’re pleased to inform you that your manuscript has been judged scientifically suitable for publication and will be formally accepted for publication once it meets all outstanding technical requirements.

Within one week, you’ll receive an e-mail detailing the required amendments. When these have been addressed, you’ll receive a formal acceptance letter and your manuscript will be scheduled for publication.

An invoice will be generated when your article is formally accepted. Please note, if your institution has a publishing partnership with PLOS and your article meets the relevant criteria, all or part of your publication costs will be covered. Please make sure your user information is up-to-date by logging into Editorial Manager at Editorial Manager® and clicking the ‘Update My Information' link at the top of the page. If you have any questions relating to publication charges, please contact our Author Billing department directly at authorbilling@plos.org.

If your institution or institutions have a press office, please notify them about your upcoming paper to help maximize its impact. If they’ll be preparing press materials, please inform our press team as soon as possible -- no later than 48 hours after receiving the formal acceptance. Your manuscript will remain under strict press embargo until 2 pm Eastern Time on the date of publication. For more information, please contact onepress@plos.org.

Kind regards,

Eda Ustaoglu, PhD

Academic Editor

PLOS ONE

Additional Editor Comments (optional):

Reviewers' comments:

Reviewer's Responses to Questions

**Comments to the Author**

1. If the authors have adequately addressed your comments raised in a previous round of review and you feel that this manuscript is now acceptable for publication, you may indicate that here to bypass the “Comments to the Author” section, enter your conflict of interest statement in the “Confidential to Editor” section, and submit your "Accept" recommendation.

Reviewer #1: All comments have been addressed

**********

2. Is the manuscript technically sound, and do the data support the conclusions?

The manuscript must describe a technically sound piece of scientific research with data that supports the conclusions. Experiments must have been conducted rigorously, with appropriate controls, replication, and sample sizes. The conclusions must be drawn appropriately based on the data presented. 

Reviewer #1: Yes

**********

3. Has the statistical analysis been performed appropriately and rigorously? 

Reviewer #1: Yes

**********

4. Have the authors made all data underlying the findings in their manuscript fully available?

The PLOS Data policy requires authors to make all data underlying the findings described in their manuscript fully available without restriction, with rare exception (please refer to the Data Availability Statement in the manuscript PDF file). The data should be provided as part of the manuscript or its supporting information, or deposited to a public repository. For example, in addition to summary statistics, the data points behind means, medians and variance measures should be available. If there are restrictions on publicly sharing data—e.g. participant privacy or use of data from a third party—those must be specified.

Reviewer #1: Yes

**********

5. Is the manuscript presented in an intelligible fashion and written in standard English?

PLOS ONE does not copyedit accepted manuscripts, so the language in submitted articles must be clear, correct, and unambiguous. Any typographical or grammatical errors should be corrected at revision, so please note any specific errors here.

Reviewer #1: Yes

**********

6. Review Comments to the Author

Please use the space provided to explain your answers to the questions above. You may also include additional comments for the author, including concerns about dual publication, research ethics, or publication ethics. (Please upload your review as an attachment if it exceeds 20,000 characters)

Reviewer #1: (No Response)

**********

7. PLOS authors have the option to publish the peer review history of their article (what does this mean?). If published, this will include your full peer review and any attached files.

If you choose “no”, your identity will remain anonymous but your review may still be made public.

**Do you want your identity to be public for this peer review?** For information about this choice, including consent withdrawal, please see our Privacy Policy.

Reviewer #1: **Yes: **Sanderson Abel

**********

# Editor report: Acceptance letter

**DOI:** 10.1371/journal.pone.0311689.r004

**Authors:** Eda Ustaoglu

14 Oct 2024

PONE-D-24-27750R1 

PLOS ONE

Dear Dr. Wang, 

I'm pleased to inform you that your manuscript has been deemed suitable for publication in PLOS ONE. Congratulations! Your manuscript is now being handed over to our production team.

At this stage, our production department will prepare your paper for publication. This includes ensuring the following:

* All references, tables, and figures are properly cited

* All relevant supporting information is included in the manuscript submission,

* There are no issues that prevent the paper from being properly typeset

If revisions are needed, the production department will contact you directly to resolve them. If no revisions are needed, you will receive an email when the publication date has been set. At this time, we do not offer pre-publication proofs to authors during production of the accepted work. Please keep in mind that we are working through a large volume of accepted articles, so please give us a few weeks to review your paper and let you know the next and final steps. 

Lastly, if your institution or institutions have a press office, please let them know about your upcoming paper now to help maximize its impact. If they'll be preparing press materials, please inform our press team within the next 48 hours. Your manuscript will remain under strict press embargo until 2 pm Eastern Time on the date of publication. For more information, please contact onepress@plos.org.

If we can help with anything else, please email us at customercare@plos.org.

Thank you for submitting your work to PLOS ONE and supporting open access. 

Kind regards, 

PLOS ONE Editorial Office Staff

on behalf of

Dr. Eda Ustaoglu 

Academic Editor

PLOS ONE
